# Effectiveness of pharmacological treatments for COVID-19 due to SARS-CoV-2: a systematic literature review 

**DOI:** 10.3389/fphar.2025.1469681

**Published:** 2025-02-28

**Authors:** Carolina Garcia Vidal, Jonathan González, Carlos Lumbreras, Miguel Salavert, Antonio Castro, Darío Rubio-Rodríguez, Carlos Rubio-Terrés

**Affiliations:** ^1^ Hospital Clinic, Infectious Diseases-IDIBAPS Department, Barcelona, Spain; ^2^ Pharmacy Department, Complejo Hospitalario Universitario de Canarias, Santa Cruz de Tenerife, Spain; ^3^ Internal Medicine Department, Hospital Universitario “12 de Octubre”, Madrid, Spain; ^4^ Infectious Diseases Unit, Hospital Universitario y Politécnico “La Fe”, Valencia, Spain; ^5^ Gilead Sciences, Madrid, Spain; ^6^ Health Value, Madrid, Spain

**Keywords:** COVID-19, SARS-Cov2 treatment, efficacy, anakinra, casirivimab/imdevimab, cilgavimab/tixagevimab, nirmatrelvir/ritonavir, regdanvimab

## Abstract

**Aim:**

Since the first cases of the COVID-19 pandemic, caused by the SARS-CoV-2 virus, described in 2019, numerous drugs have been proposed for the treatment of the disease. However, studies have given contradictory or inconclusive results, making it difficult to determine which treatments are truly effective. The objective was to carry out a systematic review of the literature analyzing the effectiveness (mortality, hospitalization and clinical improvement) of COVID-19 treatments initially proposed and finally authorized in the European Union.

**Methods:**

PubMed and other electronic databases were systematically searched for meta-analyses published between January 2020 and December 2022, as well as two additional searches: one of individual clinical studies published until October 2023 and another of those drugs that were considered at the beginning and that were discarded early because the clinical results were unfavorable.

**Results:**

In the synthesis, 85 meta-analyses and 19 additional clinical studies were included (base case). All medications indicated in the treatment of COVID-19 have favorable efficacy results (mortality, hospitalization rate, clinical improvement) but these results were not confirmed in all studies carried out, being frequently contradictory (confirming or not confirming the impact of treatment on mortality). According to meta-analysis with the largest sample size, the drugs with the greatest evidence of effectiveness in reducing mortality are remdesivir (HR= 0.79; 95% CI 0.73–0.85) and tocilizumab (OR= 0.73; 95% CI 0.56–0.93). Regarding the composite of Covid-19–related hospitalization or death from any cause, the drugs with the greatest evidence of efficacy are remdesivir, nirmatrelvir/ritonavir and sotrovimab (although, currently the effectiveness of monoclonal antibodies against the new variants of the virus has not been demonstrated).

**Conclusion:**

According to this systematic review, the treatments with the greatest evidence of reducing mortality in patients with COVID-19 are remdesivir and tocilizumab.

## 1 Introduction

Since the first cases of the COVID-19 pandemic, caused by the severe acute respiratory coronavirus 2 (SARS-CoV-2), described in the Chinese city of Wuhan in December 2019 (Huang et al., 2020), numerous drugs have been proposed to treat the disease.

The Spanish Agency of Medicines and Medical Devices (AEMPS) published a document in March 2020 that listed the “available treatments for the management of SARS-CoV-2 respiratory infection” [Agencia Española de Medicamentos y Productos Sanitarios (AEMPS), 2020]. The seventeen drug treatments initially proposed were as follows: one interleukin-1 inhibitor (anakinra); two interleukin-6 inhibitors (tocilizumab, sarilumab); four monoclonal antibodies (casirivimab/imdevimab, cilgavimab/tixagevimab, sotrovimab, regdanvimab); hydroxychloroquine or chloroquine; and, finally, eight antiviral drugs (remdesivir, favipiravir, nirmatrelvir/ritonavir, darunavir/cobicistat, interferon alfa-2b, interferon beta-1b, lopinavir/ritonavir, umifenovir and ribavirin) [Agencia Española de Medicamentos y Productos Sanitarios (AEMPS), 2020]. However, clinical trials, observational studies, and meta-analyses of these have yielded conflicting or inconclusive results, making it difficult to determine which treatments are truly effective in treating the disease.

Eight drugs are currently (5 February 2024) authorized by the European Medicines Agency (EMA) for the treatment of COVID-19: cilgavimab/tixagevimab, anakinra, nirmatrelvir/ritonavir, regdanvimab, tocilizumab, casirivimab/imdevimab, remdesivir and sotrovimab (European Medicines Agency (EMA), 2025).

The present study aims to systematically review the literature analyzing the efficacy (mortality, hospitalization and clinical improvement) of the pharmacological treatments initially proposed by the AEMPS in March 2020 [Agencia Española de Medicamentos y Productos Sanitarios (AEMPS), 2020] for COVID-19. To this end, the abundant meta-analyses of efficacy published were reviewed, being the method of evidence synthesis par excellence. A systematic review of the 151 published meta-analyses is necessary, taking into account the speed with which successive clinical studies were published and the variability of the published results. This is a systematic review of meta-analysis, so what it contributes is to review the large number of published meta-analyses and clarify the role that each treatment has had in the clinical evolution of patients affected by COVID-19.

## 2 Methods

We followed the general methodology described in two published systematic reviews (Rubio-Rodríguez et al., 2017; Grau et al., 2023)*,* as well as the *Preferred Reporting Items for Systematic Reviews and Meta-Analyses* (PRISMA) *guidelines* (Liberati et al., 2009; Moher et al., 2009; Page et al., 2021) regarding the presentation of the flowchart of the bibliographic searches carried out. The protocol, preliminary and final results reports, and article final version were approved by a panel of Spanish clinical experts in COVID-19 (co-authors of the present article).

### 2.1 Search strategy

PubMed (see search strategy in Supplementary Material 1) and other electronic databases (Cochrane Library, EMA, AEMPS and Google to identify possible grey literature) were systematically searched (Supplementary Material 1) for efficacy meta-analyses published between January 2020 and December 2022. In addition, two additional searches were conducted: one for individual clinical studies published between January 2020 and October 2023 (to identify potential individual clinical studies not included in the published meta-analyses due to exclusion criteria or because they were published after the meta-analyses), and the other focused on drugs considered potential treatments at the start of the pandemic and were discarded early on. The searches were carried out without language limitations.

### 2.2 Studies inclusion and exclusion criteria

#### 2.2.1 Systematic review of meta-analyses

##### 2.2.1.1 Inclusion criteria

In the review of meta-analyses, titles and abstracts obtained from databases and other sources were reviewed by two investigators (DRR and CRT), who assessed whether the studies met the following inclusion criteria: (1) Full text of the article (conference abstracts and posters were excluded, although letters and short articles with all the necessary information could have been accepted); (2) Analyzing the efficacy of the 17 COVID-19 drug treatments listed in the Introduction (proposed in March 2020 by the AEMPS as available treatments for the management of SARS-CoV-2 respiratory infection), for one of the following criteria: (i) all-cause mortality (usually at 28 days after randomization, although in some studies it is assessed at other intervals, e.g., 30, 60 or 90 days), which is usually the primary efficacy endpoint; (ii) other efficacy criteria, secondary endpoints (e.g., hospitalization rate, progression to invasive mechanical ventilation, progression to the need for cardiovascular support, progression to renal replacement therapy, recovery time or clinical improvement) (Shankar-Hari et al., 2021);; (3) they were meta-analyses of randomized placebo- or standard treatment-controlled clinical trials or observational studies, both direct and indirect comparisons; (4) the meta-analyses were conducted according to PRISMA guidelines (Liberati et al., 2009; Moher et al., 2009; Page et al., 2021) or Cochrane methodology (Cochrane Handbook for Systematic Reviews, 2022) or WHO Covid-Clinical Management Characterization Working Group (Shankar-Hari et al., 2021).

##### 2.2.1.2 Exclusion criteria

A large number of meta-analyses on the treatment of COVID-19 have been published, with varying degrees of quality. To select those of the highest quality, the studies initially obtained were subjected to two filters, one relating to the relevance of the journals in which they were published, and the other to the quality of the meta-analyses themselves.

Firstly, in the base case, meta-analyses were excluded if they were published in journals with an impact factor of less than 1 in the SJR (Scimago Journal Rank) index, which is freely available on the website www.scimagojr.com. A journal with an SJR value > 1.0 has an above-average citation potential, and a journal with an SJR value <1.0 has a below-average citation potential [SCImago Journal Rank (SJR), 2023]. Second, low-quality meta-analyses were excluded by analyzing them using the instrument published by the National Heart, Lung and Blood Institute (NHLBI) of the National Institutes of Health (NIH) of the United States (US) (National Heart, and Lung and Blood Institute, 2020). This scale consists of eight items. One point per item was considered, with high, medium, low and very low quality assumed for 7-8, 5-6, 3-4 and 1-2 points, respectively. Therefore, meta-analyses with a score of less than 5 were excluded. Two investigators (DRR and CRT) independently selected candidate studies for inclusion in the review, with discrepancies, if any, resolved by consensus and, if not, by a third investigator. A double check was therefore carried out to confirm the coincidence in the quality scores.

### 2.3 Additional systematic reviews

The additional systematic reviews followed the same inclusion criteria as those considered for the above but referred to individual clinical studies (randomized clinical trials or observational studies) with mortality outcomes, both for the eight drugs approved by the EMA for the treatment of COVID-19 (cilgavimab/tixagevimab, anakinra, nirmatrelvir/ritonavir, regdanvimab, tocilizumab, casirivimab/imdevimab, remdesivir and sotrovimab). Moreover, for the nine drugs that were discarded early because of unfavorable or insufficient clinical results (eculizumab, danoprevir, APN01, leronlimab, thymosin alfa1, REGN3084/REG3051).

Although only meta-analyses with an impact factor greater than 1 were included in the base case, an additional review of meta-analyses with an impact factor less than 1 in the SJR index was performed.

### 2.4 Data extraction

For the systematic meta-analysis review, the data extracted from the articles to be included were as follows: 1) Year of publication; 2) First author’s surname; 3) Type of meta-analysis (random-effects model, fixed-effects model, Bayesian model, network meta-analysis (NMA), mixed treatment comparison (MTC), indirect treatment comparison (ITC), and others); 4) Design of the clinical studies included in the meta-analysis (randomized clinical trials, observational studies [cohort, case-control, prospective or retrospective, etc.); 5) Comparator in the clinical studies (standard treatment, placebo control, etc.); 6) Main efficacy endpoints (mortality, disease progression, admission to ICU with mechanical ventilation, risk of secondary infection, hospital discharge, etc.); 7) Number of studies included in the meta-analysis; 8) Number of patients included in the meta-analysis; 9) Result of the measurement of the effect of the treatments compared to placebo or standard treatment (relative risk [RR], odds ratio [OR], hazard ratio [HR], risk difference, etc.) specifying - if available - the 95% confidence intervals (95% CI) and the statistical significance of the difference (p); 10) Degree of heterogeneity in the meta-analysis (I^2^), according to the following criteria: (i) up to 25%, low heterogeneity; between >25% and <75%, moderate heterogeneity; and (iii) if ≥ 75%, high heterogeneity (Higgins et al., 2003); and finally, 11) Effect size, calculated using Cohen’s d; by convention, Cohen’s d of 0–0.4, 0.5–0.7 and ≥0.8 are considered small, medium and large effect sizes, respectively (Chen et al., 2010). Cohen’s d will be calculated using the tool available at the following URL: https://www.escal.site/.

Data extraction was carried out by one investigator (DRR) and reviewed by another investigator (CRT).

### 2.5 Meta-analysis assessment criteria

As noted above, available clinical trials, observational studies and meta-analyses have yielded conflicting or inconclusive results, making it difficult to determine which drug treatments are truly effective for the treatment of COVID-19. Discrepancies in results between meta-analyses are difficult to resolve, as meta-analyses of meta-analyses are impossible. There is no consensus on the appropriateness of mixing observational studies and randomized clinical trials in the same meta-analysis (Bosdriesz et al., 2020; Kimachi et al., 2021; Ranstam and Wagner, 2022; Shrier et al., 2007; Toews et al., 2024) as is the case in part of the COVID-19 treatment syntheses. Meta-analysis is a statistical method used to combine the results of individual studies, obtaining a larger sample size, which provides greater reliability (precision) of treatment effect estimates (Higgins and Green, 2011). On the other hand, a larger sample size reduces the risk of type II error (Bausch and Cartwright, 2021; Rubio, 1996). Consequently, meta-analyses with larger sample sizes and the latest published meta-analyses (generally with a larger sample size due to the inclusion of the most recently published studies) were analyzed in preference. The risk of bias of individual clinical studies (in aspects such as randomization process, deviations from intended interventions, missing outcome data, measurement of the outcome, selection of the reported results) was analyzed in most of the meta-analyses, but was not analyzed in the systematic review because it is a synthesis of synthesis studies. This systematic review has not been registered.

## 3 Results

### 3.1 Results of the bibliographic searches

**FIGURE 1:**
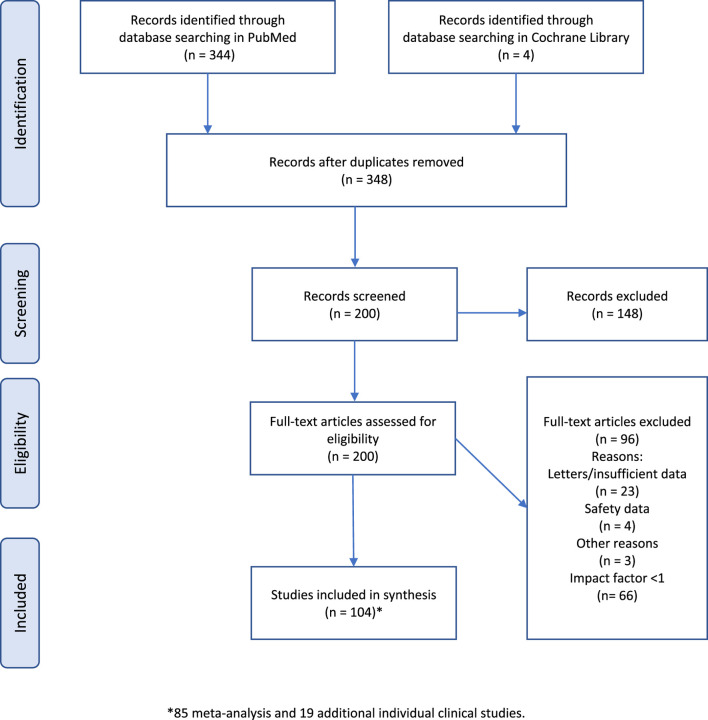
Flowchart of the bibliographic searches carried out (base case).

The three bibliographic searches yielded 348 references. Of these, 148 were excluded because they did not meet the inclusion criteria. The full articles of the remaining 200 references were analyzed for eligibility. Of these, 96 were excluded. Accordingly, 104 articles were selected for inclusion in the synthesis. Please see Figure 1 for the study selection process according to PRISMA guidelines (all articles included in the base case of the synthesis can be found in Supplementary Material 2, in alphabetical order). The 96 references finally excluded were excluded for the following reasons: (i) 23 because they were letters to the editor or articles with insufficient or contradictory data (confirming or not confirming the impact of treatment on mortality); (ii) 4 because they only analyzed adverse effects of treatments; (iii) 3 because they were a protocol, analyzed a drug combination or virological sensitivity; and finally (iv) 66 because they had an impact factor of less than 1 in the SJR index (Supplementary Material 3). No meta-analysis was excluded for having a score less than 5 according to the NHLBI instrument. All meta-analyses analyzed were above this score.

The articles on the drugs discarded early for the treatment of COVID-19 are listed in Supplementary Material 4.

### 3.2 Meta-analyses included in the synthesis (base case)

In Table 1, the number of meta-analyses selected in the systematic review is given for each drug or pharmacological group (*literature references in* Supplementary Material 2). In total, 85 meta-analyses were selected and analyzed (one meta-analysis was able to analyze several drugs). The characteristics of the meta-analyses included in the synthesis are comprehensively detailed in Supplementary Tables S1–S9.

**TABLE 1:** Number of meta-analyses obtained, according to the drug or pharmacological group.

Drug of pharmacological group	Number of meta-analysis selected	Tables Supplementary Material S5
Interleukin 1 inhibitor: Anakinra	7	S1
Interleukin 6 inhibitor: Tocilizumab	25	S2
Interleukin 6 inhibitor: Sarilumab	6	S3
Monoclonal antibodies: Casirivimab/imdevimab, Cilgavimab/tixagevimab, Sotrovimab, Regdanvimab	6	S4 (a & b)^1^
Hydroxychloroquine or Chloroquine	16	S5
Antivirals: Remdesivir	12	S6 (a & b)^2^
Antivirals: Favipiravir	4	S7
Antivirals: Nirmatrelvir/ritonavir	3	S8
Other antivirals: Darunavir/cobicistat, IFN-alpha, IFN-beta, Lopinavir/ritonavir, Umifenovir, Ribavirin	6	S9
Total	85	-

IFN: interferon. (1) Including 1 RCT, and one cohort study (Supplementary Table S4B); (2) Including four cohort studies (Supplementary Table S6B).

### 3.3 Clinical studies justifying approval in COVID-19

**FIGURE 2:**
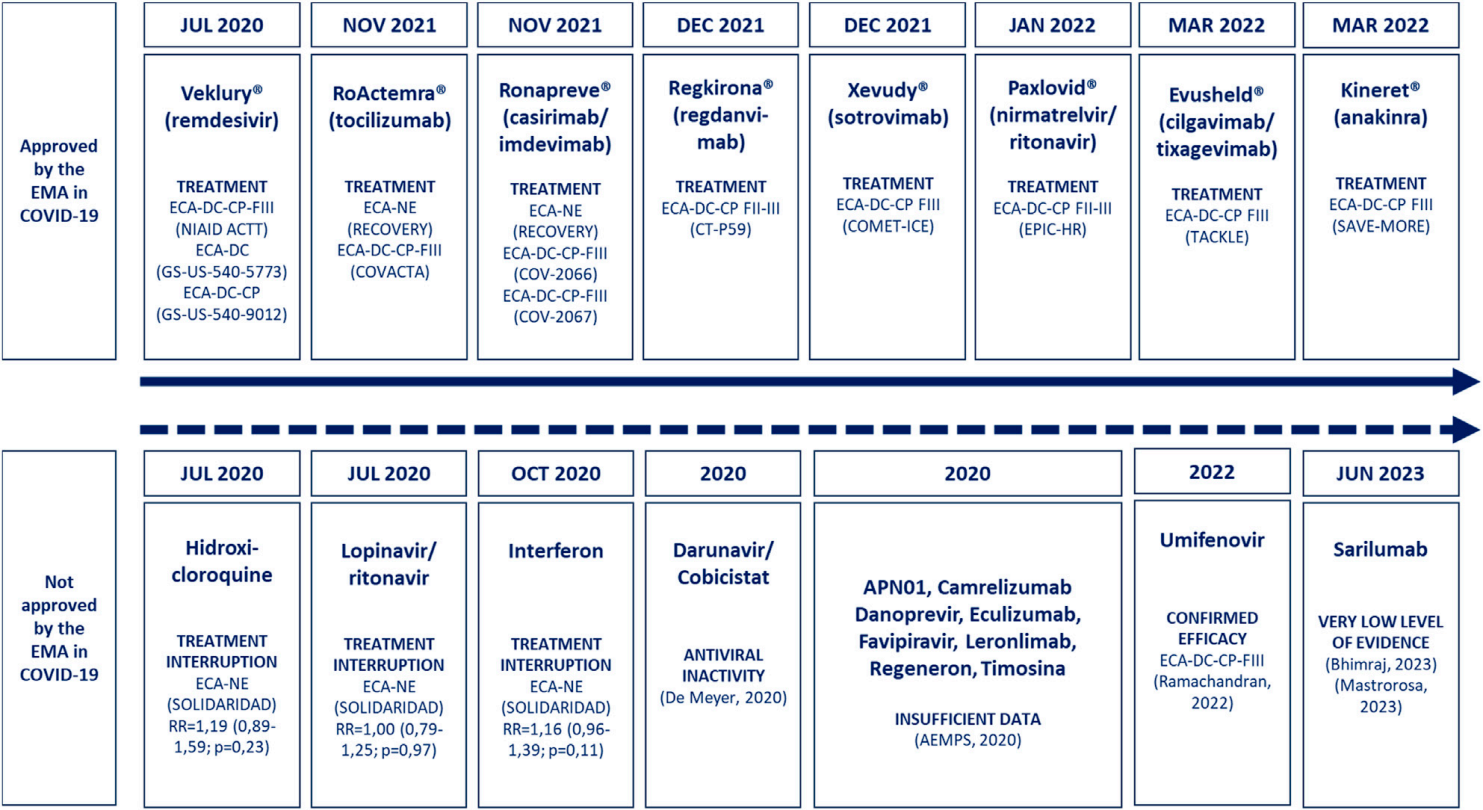
Timeline of drugs approved for the treatment of COVID-19. The dates are those of approval by the EMA and those of the drugs not approved by the EMA, but that were initially proposed by the AEMPS due to their possible usefulness in the treatment of COVID-19. Abbreviations: AEMPS, Spanish Agency for Medicines and Health Products; CP, placebo controlled; DC, double blind; RCT, randomized clinical trial; EMA, European Medicines Agency; FIII, phase III clinical trial; NE, not masked.

In Figure 2, a timeline diagram of the drugs approved or discarded for COVID-19 treatment is presented, specifying the milestones that marked the EMA’s decisions in this regard. Table 2 (bibliographic references in Supplementary Material 2) contains a summary of the characteristics and results of the clinical trials that justify approval of the indication in COVID-19 by the EMA. The following effects were found in these studies (Table 2): (i) clinical improvement, shorter recovery time, and reduction in COVID-19 hospitalization or mortality with remdesivir (Beigel et al., 2020; Goldman et al., 2020; Gottlieb et al., 2022); (ii) clinical improvement and reduced mortality with tocilizumab (RECOVERY Collaborative Group et al., 2022; Rosas et al., 2021); (iii) reduction of mean viral load and mortality with casirimab/imdevimab (Abbas et al., 2022; CEDER. Center for Drug Evaluation and Research CDER Review, 2021); (iv) reduction of hospitalization, oxygen therapy and mortality with regdanvimab (Celltrion use of regdanvimab for the treatment of COVID-19. Assessment report, 2021); (v) reduction in hospitalization or death with sotrovimab (Gupta et al., 2021); (vi) reduction in hospitalization or death with nirmatrelvir/ritonavir (Hammond et al., 2022); (vii) reduction in the incidence of severe COVID-19 or death with cilgavimab/tixagevimab (Evusheld, 2019); and, finally, (viii) reduction in WHO-CPS progression of COVID-19 with anakinra (Kyriazopoulou et al., 2021).

**TABLE 2:** Main clinical studies that justified the approval of drugs for the treatment of COVID-19.

Drugs	Year of publication	First author (study name) §	Design	Treatments	Doses	No. of patients	Efficacy endpoints	OR/RR/HR (95% CI; p)	Magnitude of effect: Cohen’s “d” *
Remdesivir	2020	Beigel (NIAID ACTT)	RCT-DB-PC	Remdesivir Placebo	200 mg (day 1), 100 mg/día (9 days) (10 days)	541 521	Recovery time (29 days) Mortality (29 days)	1.29 (1.12–1.49; <0.001) 0.73 (0.52–1.03, 0.07)	S: 0.140 No association
	2022	Goldman (GS-US-540-5773)	RCT-DB	Remdesivir Remdesivir	200 mg (day 1), 100 mg/día (4 days) 200 mg (day 1), 100 mg/día (9 days)	200 197	Clinical improvement (day 14) with the 10-day treatment	0.67 (0.46–0.98)	S: 0.221
	2022	Gottlieb (GS-US-540-9012)	RCT-DB-PC	Remdesivir Placebo	200 mg (day 1), 100 mg/día (2 days) (3 days)	279 283	COVID-19 hospitalization or mortality (28 days)	0.13 (0.03–0.59, 0.008)	L: 1.12
Tocilizumab	2021	RECOVERY (RECOVERY)	RCT-NE	Tocilizumab Usual treatment	400–800 mg single dose	2022 2094	Mortality (28 days) Hospital discharge (28 days)	0.85 (0.76–0.94, 0.0028) 1.22 (1.12–1.33; <0.0001)	S: 0.090 S: 0.110
	2021	Rosas (COVACTA)	RCT-DB-PC	Tocilizumab Placebo	8 mg/kg	294 144	Clinical improvement (28 days) Mortality (28 days)	−1.0 (−2.5; 0.0; 0.31) 0.3 (−7.6:8.2; 0.94)	No association No association
Casirimab/Imdevimab (C/I)	2022	RECOVERY (RECOVERY)	RCT-NE	C/I Usual treatment	4.000/4.000 mg	4839 4946	Mortality (28 days)	0.79 (0.69–0.91, 0.0009)	S: 0.130
	2021	CEDER (COV-2066)	RCT-DB-PC	C/I Placebo	4.000/4.000 mg	398 393	Reduction in average viral load	−0.28 log10 copies/mL/day (p = 0.0172)	NA
	2021	CEDER (COV-2067)	RCT-DB-PC	C/I Placebo	600/600 mg 1.200/1.200 mg	1347 2036 2009	Reduction in risk of 1 or more hospitalizations due to COVID-19 or mortality (29 days)	0.27/0.29 (<0.0001)	M: 0.722/0.682
Regdanvimab	2021	Celltrion, Regkirona (CT-P59 3.2.)	RCT-DB-PC	Regdanvimab Placebo	40 mg/kg	446 434	Proportion of patients with clinical symptoms requiring hospitalization, oxygen therapy, or mortality due to SARS-CoV-2 infection (28 days)	−8.0% (−11.7%; −4.5%; <0.0001)	NA
Sotrovimab	2021	Gupta, Xevudy (COMET-ICE)	RCT-DB-PC	Somotrimab Placebo	500 mg single dose	291 292	Hospitalization or death (29 days)	0.21 (0.09–0.50; <0.001)	L: 0.860
Nirmatrelvir/Ritonavir (N/R)	2022	Hammond (EPIC-HR)	RCT-DB-PC	N/R Placebo	300/100 mg	1120 1126	Hospitalization or death (28 days)	0.109 (<0.001)	L: 1.22
Cilgavimab/Tixagevimab (C/T)	2022	Evusheld (TACKLE)	RCT-DB-PC	C/T Placebo	300/300 mg single dose	3460 1737	Incidence of severe COVID-19 or death from any cause until the 29 days	0.58 (0.36–0.95; 0.028)	S: 0.300
Anakinra	2021	Kyriazopoulou (SAVE-MORE)	RCT-DB-PC	Anakinra Placebo	100 mg/day	405 189	WHO-CPS scale effectiveness (28 days)	0.40 (0.29–0.55; 0.0001)	M: 0.505

Abbreviations: C/I, Casirimab/Imdevimab; C/T, Cilgavimab/Tixagevimab; RCT-DB-PC, randomized, double-blind, placebo-controlled clinical trial; HR, hazard ratio; 95% CI, 95% confidence interval; NA, not available; NE, unmasked; OR, odds ratio; p, statistical significance; N/R, Nirmatrelvir/Ritonavir; RR, relative risk. * By convention, for Cohen’s “d” of 0–0.4; 0.5–0.7 and ≥0.8 are considered small (S), medium (M) and large (L) effect sizes respectively (*Chen, 2010*). Cohen’s d was calculated using the tool available at the following URL: https://www.escal.site/. § Full references in Supplementary Material S1.

### 3.4 Variability in the results of meta-analyses: possible role of sample size

**FIGURE 3:**
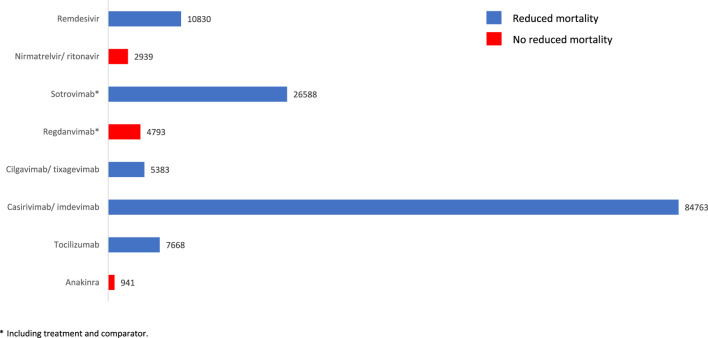
Sample size of meta-analyses or individual clinical studies of authorized treatments for COVID-19 and reduction in associated mortality. * Including treatment and comparator.

The effect on mortality of COVID-19 drug treatments was highly variable in the different published meta-analyses, ranging from no association to a small, medium or large mortality effect (Supplementary Figure S1; Supplementary Material 6). As can be seen in Figure 3, the sample size effect of individual meta-analyses or clinical studies of licensed treatments for COVID-19 was associated with the demonstration of mortality reduction. Indeed, a reduction in mortality was observed in meta-analyses with a sample size of more than 5,000 patients (remdesivir, sotrovimab, cilgavimab/tixagevimab, casirivimab/imdevimab and tocilizumab), but not in meta-analyses with fewer than 5,000 patients (nirmatrelvir/ritonavir, regdanvimab and anakinra). It should be clear that the possible role of sample size in the results obtained is a mere hypothesis, simply the description of a result.

### 3.5 Mortality in recent meta-analyses

In Table 3, the reduction in COVID-19 mortality for the 17 treatments initially proposed is summarized, considering the outcome obtained in the most recent meta-analyses of randomized clinical trials (or, if not available, also including observational studies). Looking first at meta-analyses of randomized clinical trials, mortality was significantly reduced with tocilizumab [OR = 0.78 (95% CI 0.65–0.94)] (Albuquerque et al., 2023), casirivimab/imdevimab [OR = 0.67 (95% CI 0.50–0.91)] (Deng et al., 2023), sotrovimab [OR = 0.20 (95% CI 0.08–0.48)] (Deng et al., 2023) and remdesivir [RR = 0.83 (95% CI 0.71–0.98)] (Huang et al., 2023). In meta-analyses including observational studies, mortality reduction was observed with cilgavimab/tixagevimab [OR = 0.50 (95% CI 0.39–0.64)] (Wang et al., 2023), regdanvimab [OR = 0.14 (95% CI 0.03–0.56)] (Yang et al., 2022) and nirmatrelvir/ritonavir [OR = 0.24 (95% CI 0.15–0.39)] (Cheema et al., 2023). No reduction in mortality was observed with anakinra, sarilumab, antimalarials and the antivirals darunavir/cobicistat, favipiravir, interferons alpha and beta, lopinavir/ritonavir and umifenovir. At present, it should be noted that none of the monoclonal antibodies approved or registered for clinical use maintains proven clinical efficacy against the Omicron variant and its successive evolving sub-variants due to insufficient virus-neutralizing or blocking activity (Coutant et al., 2024; CovidCAREgroup, 2022). Consequently, it can be concluded that, according to the most recent meta-analyses, the treatments with the most evidence of mortality reduction in patients with COVID-19 would be remdesivir and tocilizumab. The magnitude of the effect with both drugs was small (Cohen’s d < 0.5) (Table 3).

**TABLE 3:** Reduction in mortality from COVID-19 for the different proposed treatments, considering the result obtained in the most recent meta-analyses of RCTs (or, if they are not available, of RCTs and/or observational studies), with possible causes of heterogeneity in the included clinical studies (risk of bias, % vaccinated patients against COVI-19, concomitant treatments, severity on admission, invasive mechanical ventilation).

Drugs	Author (year)§	Period	Result RR, OR, HR (95% CI; p)	No. patients treated	Magnitude of effect: Cohen’s “d” *	Risk of bias	% Vaccinated against COVID-19	Concomitant treatments	Severity on admission	Invasive mechanical ventilation
Interleukin 1 inhibitors										
Anakinra ¶	Shang (2023)	90 days	1.01 (0.73–1.39, 0.97)	196	No association	NA	NA	NA	Mixed severity	NA
Interleukin 6 inhibitors										
Sarilumab	Albuquerque (2023)	28 days	0.91 (0.60–1.40; NA)	703	No association	Low	NA	With or W/O SoC	NA	0%–54% of patients
Tocilizumab ¶	Albuquerque (2023)	28 days	0.78 (0.65–0.94; NA)	3042	Small	Low	NA	With or W/O SoC	NA	0%–100% of patients
Monoclonal antibodies**										
Casirivimab/imdevimab ¶	Deng (2023)	30–90 days	0.67 (0.50–0.91; NA)	NA	Small	High: 10,9%	NA	With or W/O SoC	Mixed severity	With or W/O ventilation
Cilgavimab/tixagevimab ¶	Wang# (2023)	NA	0.50 (0.39–0.64; NA)	5383	Small#	NA	NA	NA	NA	NA
Regdanvimab ¶	Yang# (2022)	NA	0.14 (0.03–0.56; 0.006)	789	Large#	NA	NA	NA	NA	NA
Sotrovimab ¶	Deng (2023)	30–90 days	0.20 (0.08–0.48; NA)	ND	Large	High: 10,9%	NA	With or W/O SoC	Mixed severity	With or W/O ventilation
Antimalarials										
Hydroxychloroquine or Chloroquine	Gupta (2022)	28 days	1.08 (0.99–1.19; NA)	3788	No association	Low	NA	With or W/O SoC	NA	With or W/O ventilation
Antivirals										
Darunavir/cobicistat	Okoli (2022)	NA	1.00 (0.02–5.10; NA)	ND	No association	High/ND: 80%	NA	NA	Mixed severity	NA
Favipiravir	Özlüşen# (2021)	NA	1.11 (0.64–1.94; 0.69)	823	No association#	Moderate/High	NA	With or W/O SoC	Mixed severity	With or W/O ventilation
IFN-alpha	Buchynskyi# (2023)	NA	0.25 (0.05–1.19; 0.082)	ND	No association#	Moderate/High	NA	NA	Mixed severity	NA
IFN-beta	Okoli (2022)	NA	0.43 (0.08–1.18; NA)	ND	No association	High/ND: 80%	NA	NA	Mixed severity	NA
Lopinavir/ritonavir	Okoli (2022)	NA	0.95 (0.78–1.15; NA)	ND	No association	High/ND: 80%	NA	NA	Mixed severity	NA
Nirmatrelvir/ritonavir ¶	Cheema# (2023)	NA	0.24 (0.15–0.39; <0.00001)	ND	Median#	NA	Vaccinated/Unvaccinated	NA	Mixed severity	NA
Remdesivir ¶	Huang (2023)	28 days	0.83 (0.71–0.98; NA)	2230	Small	Moderate/High	NA	With or W/O SoC	Mixed severity	With or W/O ventilation
Umifenovir	Yu (2021)	NA	0.32 (0.10–1.09; 0.07)	84	No association	Low/ND	NA	NA	Mixed severity	NA

Abbreviations: HR, hazard ratio; IFN, interferon; NA, data not available; ND, not determined; OR, odds ratio; p, statistical significance; RCT, randomized clinical trials; RR, relative risk; SoC, standard of care (with or W/O corticosteroids); W/O, without. *By convention, for Cohen’s “d” of 0.1–0.4; 0.5–0.7 and ≥0.8 are considered small, medium, and large effect sizes, respectively (*Chen, 2010*). Cohen’s d was calculated using the tool available at the following URL: https://www.escal.site/. ** At the current time it should be taken into account that none of the monoclonal antibodies approved or registered for clinical use maintains demonstrated clinical efficacy against the Omicron variant and its successive evolutionary subvariants, due to insufficient virus neutralizing or blocking activity. ^¶^Medications authorized for the treatment of covid-19. # Meta-analysis that included observational studies or non-randomized trials: Cilgavimab/tixagevimab (*Wang, 2023*): included 3 RCTs and two cohort studies; Regdanvimab (*Yang, 2022*): included 1 RCT and six retrospective observational studies; Favipiravir (*Özlüşen, 2021*): included 3 RCTs, 7 ECs and two observational; IFN-alpha (*Buchynsky, 2023*): included one clinical trial and 10 observational studies; Nirmatrelvir/ritonavir (*Cheema, 2023*): included 2 RCTs and 10 observational studies. § Full references in Supplementary Material S2.

### 3.6 Mortality in meta-analyses with larger sample sizes

An additional analysis of the worst and best outcome in terms of mortality reduction, obtained in meta-analyses and individual clinical studies with a larger sample size, was performed (Table 4). In this sense, “probable” or “inconclusive” efficacy was considered when the result was positive (mortality reduction) or negative (no mortality reduction), respectively, in the study with the largest sample size. Thus, mortality reduction was considered likely with tocilizumab [OR = 0.73 (95% CI 0.56–0.93)] (Rubio-Rivas et al., 2021), casirivimab/imdevimab [OR = 0.21 (95% CI 0.06–0.68)] (Gao et al., 2023), cilgavimab/tixagevimab [OR = 0.50 (95%CI 0.39–0.64)] (Wang et al., 2023), sotrovimab [OR = 0.40 (95% CI 0.25–0.63)] (Amani and Amani, 2022) and remdesivir [OR = 0.79 (95% CI 0.73–0.85) (Low-flow oxygen patients)] (Mozaffari et al., 2023). The effect size was variable, being small (Cohen’s d 0.5) with tocilizumab, cilgavimab/tixagevimab and remdesivir, medium (Cohen’s d between 0.5 and 0.7) with sotrovimab, and large (Cohen’s d ≥ 0.8) with casirivimab/imdevimab. Again, due to doubts about the efficacy of monoclonal antibodies (Coutant et al., 2024; CovidCAREgroup, 2022), the treatments with the most evidence of mortality reduction in patients with COVID-19 would be remdesivir and tocilizumab, according to meta-analyses and larger studies.

**TABLE 4:** Reduction in mortality from COVID-19 for the different proposed treatments, considering the worst and best results obtained both in the meta-analyses and in individual clinical studies with a larger sample size. Only the results of the eight drugs authorized for the treatment of COVID-19 are presented. Those treatments whose worst outcome was favorable and with statistical significance are highlighted (underlined, bold).

Drugs	Worst result RR, OR, HR (95% CI)	No. patients treated	References (Supplementary Material S2)	Magnitude of effect: Cohen’s “d”**	Best result RR, OR, HR (95% CI)	No. patients treated	References (Supplementary Material S2)	Magnitude of effect: Cohen’s “d”**	Reduction in mortality from COVID-19*
Interleukin 1 or 6 inhibitors										
Anakinra	1.01 (0.73–1.39)	941	Shang, 2023	No association	0.32 (0.23–0.45)	485	Barkas, 2021	Medium	Inconclusive	
**Tocilizumab**	0.88 (0.81–0.94)	7.428	Ghosn, 2023	Small	0.73 (0.56–0.93)	7668	Rubio-Rivas, 2021	Small	Probable	
Monoclonal antibodies #										
Casirivimab/imdevimab	0.58 (0.26–1.22)	ND	Siemieniuk, 2021	No association	0.21 (0.06–0.68)	84,763	Gao, 2023	Large	Probable	
**Cilgavimab/tixagevimab**	0.70 (0.50–0.97)	710	ACTIVE-3	Small	0.50 (0.39–0.64)	5383	Wang, 2023	Small	Probable	
Regdanvimab	0.46 (0.11–1.89)	4793^¶^	Amani, 2023	No association	0.14 (0.03–0.56)	789	Yang, 2022	Large	Inconclusive	
Sotrovimab	0.36 (0.08–1.66)	1040	Ao, 2022	No association	0.40 (0.25–0.63)	26,588^¶^	Amani, 2022	Medium	Probable	
Antivirals										
Nirmatrelvir/ritonavir	Not estimable***	2939¶	Petersen, 2023	Medium	0.04 (0.00–0.68)	2224	Reis, 2022	Large	Inconclusive	
**Remdesivir**	0.88 (0.78–1.00)§	5398	Amstutz, 2023	No association	0.79 (0.73–0.85)	10,830	Mozaffari, 2023a	Small	Probable	

*Mortality on day 28 (when data is available). It is considered “probable” when the result is positive (reduction in mortality) for the study with a larger sample size. It is considered “inconclusive” when, on the contrary, there is a negative result (no reduction in mortality) for the study with the largest sample size.

**By convention, for Cohen’s “d” of 0.1–0.4; 0.5–0.7 and ≥0.8 are considered small, medium, and large effect sizes, respectively (*Chen, 2010*). Cohen’s d was calculated using the tool available at the following URL: https://www.escal.site/

***Due to the insufficiency of available data (*Petersen, 2023*).

# At the current time it should be taken into account that none of the monoclonal antibodies approved or registered for clinical use maintains demonstrated clinical efficacy against the Omicron variant and its successive evolutionary subvariants, due to insufficient virus neutralizing or blocking activity.

¶Including treatment and comparator.

§p = 0.045.

Abbreviations: HR, hazard ratio; OR, odds ratio; RR, relative risk.

### 3.7 Mortality and hospitalization rate

Considering the reduction in Covid-19-related hospitalization or death from any cause (generally up to day 28) as a combined efficacy endpoint, casirivimab/imdevimab [RR = 0.28–0.29; p < 0.0001] (Ronapreve, 2024 300 mg + 300), sotrovimab [RR = 0.21 (95% CI 0.09–0.50)] (Xevudy, 2021), nirmatrelvir/ritonavir [-5.62% (95% CI -7.21; −4.03%)] (Hammond et al., 2022) and remdesivir [RR = 0.13 (95% CI 0.03–0.59)] (Gottlieb et al., 2022) were effective. The magnitude of the effect was large (Cohen’s d ≥ 0.8) with sotrovimab and remdesivir.

### 3.8 Other clinical effects: secondary efficacy endpoints

#### 3.8.1 Anakinra

In one of the meta-analyses (Kim et al., 2020), a significant effect on progression to severe disease was observed. For other parameters, such as ICU admission requiring mechanical ventilation, the magnitude of the effect ranged from small to medium (Barkas et al., 2021; Pasin et al., 2021). The effect of treatment on the risk of secondary infection was not confirmed (Peng et al., 2022) (Supplementary Table S1).

#### 3.8.2 Tocilizumab

In most studies, no association was found between tocilizumab and other efficacy parameters, such as risk of ICU admission, need for mechanical ventilation, hospital discharge or clinical improvement (Supplementary Table S2).

#### 3.8.3 Monoclonal antibodies

With casirivimab/imdevimab and sotrovimab, there were conflicting results regarding the need for mechanical ventilation, but the outcome was clearly positive regarding the reduction of hospitalization (Supplementary Table S4).

#### 3.8.4 Nirmatrelvir/ritonavir

With nirmatrelvir/ritonavir, there was a significant effect on clinical improvement and reduction in hospitalization (Supplementary Table S8).

#### 3.8.5 Remdesivir

With remdesivir, there was a clear effect on clinical improvement and recovery, as well as on mechanical ventilation (Supplementary Table S6).

### 3.9 Potential treatments not currently authorized by the EMA

All eight medicines authorized for the treatment of COVID-19 have at least one randomized, double-blind, placebo-controlled clinical trial (Figure 2). Several drugs initially proposed but not licensed for the treatment of COVID-19 (sarilumab, hydroxychloroquine/chloroquine, darunavir/cobicistat, favipiravir, IFN-alpha/IFN-beta, lopinavir/ritonavir and umifenovir) have been discussed previously (Section 3.5; Table 3). Insufficient demonstrative efficacy data may be the reason why these drugs have not been approved for the treatment of COVID-19 (Figure 2; Supplementary Material 4). The data available for other drugs not authorized for COVID-19 are reviewed in more detail below: eculizumab, danoprevir, APN01, leronlimab, camrelizumab and thymosin α1.

Eculizumab is a recombinant humanized IgG2/4κ monoclonal antibody that binds to human complement protein C5 and inhibits terminal complement activation (Bekemv 300 mg concentrado para solución para perfusión, 2021). It has only one feasibility study that concluded it could improve survival (randomized clinical trials would be necessary to confirm this) (Annane et al., 2020), two case series (Diurno et al., 2020; Burwick et al., 2022) and one observational (cohort, retrospective) study comparing 10 eculizumab-treated patients with 65 controls, a small sample size that precluded valid conclusions (Ruggenenti et al., 2021).

Danoprevir is an NS3/4A protease inhibitor used to treat HCV genotype (GT) 1b infections (Miao et al., 2020). The results of a cohort study evaluating the efficacy of treatment with danoprevir plus ritonavir in 11 patients with COVID-19 have been published (Chen et al., 2020). The study design precludes conclusions about the efficacy of danoprevir.

APN01 is the recombinant form of human angiotensin-converting enzyme 2 (rhACE2) that may prevent SARS-CoV-2 entry into the host cell and reduce lung injury (EUnetHTA, 2021). Three clinical studies of COVID-19 treatment with APN01 are registered in the clinicaltrials.gov database, but results are only available for one of them (NCT04335136) (COVID-19, 2023). This is a randomized, double-blind, placebo-controlled clinical trial. Eighty-eight patients were treated with APN01, and 90 received a placebo. Mortality at 28 days was 10.2% and 13.3%, respectively, with no statistically significant difference (OR = 0.63, 95% CI 0.23–1.70, p = 0.3588).

Leronlimab is a C-C chemokine receptor type 5 blocking monoclonal antibody originally developed to treat human immunodeficiency virus infection (Yang et al., 2021). Five clinical studies of COVID-19 treatment with leronlimab are registered in the clinicaltrials.gov database, but results are only available for one of them (NCT04343651) (COVID-19 (APN01-COVID-19), 2024). This is a randomized, quadruple-blind, placebo-controlled clinical trial. Fifty-six patients were treated with leronlimab, and 28 received a placebo. There was no mortality at day 14 in either group. There were also no differences in symptom score, time to clinical resolution, or length of hospitalization.

Camrelizumab is a monoclonal antibody proposed as a potential treatment for COVID-19, but no clinical studies have been identified that have evaluated its efficacy in this indication.

Thymosin α1 has been used in the treatment of viral infections as an immune response modifier for many years (Liu et al., 2020). In a retrospective study, compared to the untreated group (N = 40), treatment with thymosin α1 (N = 36) significantly reduced mortality in severe COVID-19 patients (11.11% vs. 30.00%, p = 0.044) (Liu et al., 2020).

### 3.10 Additional analysis of studies excluded in the base case for being published in journals with low SJR impact factor (<1)

A total of 151 meta-analyses have been identified. This high number is due to the health and social relevance of the COVID-19 epidemic and, therefore, to the tendency of the many medical journals to publish meta-analyses that could clarify which treatments would be the most effective in this context. For this reason, the systematic review was divided into two analyses according to the supposed quality of the studies according to the impact factor of the medical journals. The full references of the initially excluded studies are attached in Supplementary Material 3. As can be seen in Supplementary Material 7, most meta-analyses of immunomodulatory drugs (anakinra, tocilizumab, sarilumab) did not show a statistically significant effect or had a small magnitude with respect to mortality. Regarding antimalarials (hydroxychloroquine, chloroquine), there was no effect on mortality in 16 of 18 meta-analyses. Finally, regarding antivirals, a small effect on mortality was found in one meta-analysis of favipiravir, in two meta-analyses of interferon and in two meta-analyses of remdesivir.

## 4 Discussion

First of all, a word of caution is in order. The intention of any systematic review - and therefore of this one - is to identify and analyze all studies that meet the previously established inclusion criteria. However, it is important to remember that no matter how extensive and detailed the literature searches are, there is no absolute certainty that all published studies suitable for this synthesis have been obtained.

At the onset of the COVID-19 pandemic, the need to find effective treatments in as short as possible became apparent. For this reason, numerous drugs were initially proposed as potential treatments based on the characteristics of the disease and their pharmacological activities [Agencia Española de Medicamentos y Productos Sanitarios (AEMPS), 2020] Of the 17 proposed drugs, only eight were finally approved by the EMA for the treatment of COVID-19 (European Medicines Agency (EMA), 2025). However, with regard to the drugs indicated for the treatment of the disease, several aspects could question the conclusions of the study. Limitations of the study are outlined below. Firstly, the considerable number of meta-analyses published is striking. Secondly, it should be noted that contradictory results confirming or denying the efficacy of different treatments on mortality abound. The repetition of meta-analyses is justified by the need to incorporate the results of new clinical studies as they are published. This would also explain why the latest meta-analyses, those with larger sample sizes, have confirmed the efficacy results, which were questioned in some initial meta-analyses, with smaller patient samples and therefore lower statistical power, probably insufficient to demonstrate real differences in efficacy (Sigman, 2011). For this reason, the results of the most recent meta-analyses and those with the largest number of patients have been highlighted. This is the main strength of this study.

The main limitation of our study is given by the heterogeneity of the individual clinical studies included in the reviewed meta-analyses. This has been highlighted by the investigators who published the meta-analyses themselves (Albuquerque et al., 2023; Wang et al., 2023). For this reason, we have performed an additional analysis of the main determinants of heterogeneity (risk of bias, COVID-19 vaccination, concomitant treatments, disease severity on admission, invasive mechanical ventilation) among the clinical studies included in the most recent meta-analyses, which is summarized in Table 3. As can be seen, the meta-analyses did not analyze these aspects separately, they jointly analyzed patients with different severity, with or without concomitant treatments, with or without mechanical ventilation, as well as studies with different risks of bias. This confirms the considerable heterogeneity of the studies included in the different meta-analyses. Although it is outside the scope of our systematic review, it would be interesting to perform a new meta-analysis in the future in which stratified analyses were performed with respect to the heterogeneity factors mentioned above.

Moreover, the conclusions of meta-analyses and clinical trials conducted with monoclonal antibodies should now be called into question, as none of the monoclonal antibodies approved or registered for clinical use maintain proven clinical efficacy against the Omicron variant and its successive evolving sub-variants, due to insufficient virus neutralizing or blocking activity (Coutant et al., 2024; CovidCAREgroup, 2022).

## 5 Conclusion

In the present synthesis, 85 meta-analyses and 19 additional clinical studies were included (in the base case). All the drugs indicated for the treatment of COVID-19 had favorable efficacy results (mortality, hospitalization rate, clinical improvement), but these results were not confirmed in all the studies conducted and were often contradictory (confirming or not confirming the impact of treatment on mortality). According to meta-analyses with larger sample sizes, the drugs with the most evidence of effectiveness in reducing mortality are remdesivir (HR = 0.79, 95% CI 0.73–0.85) in low-flow oxygen patients and tocilizumab (OR = 0.73, 95% CI 0.56–0.93). In terms of the composite of hospitalization or death from any COVID-19-related cause, the drugs with the strongest evidence of efficacy are remdesivir, nirmatrelvir/ritonavir and sotrovimab (although the efficacy of monoclonal antibodies against the new variants of the virus is currently unproven). According to this systematic review, the treatments with the most evidence of mortality reduction in patients with COVID-19 are remdesivir and tocilizumab. This is a systematic review of meta-analyses and these meta-analyses did not discriminate between vaccinated and unvaccinated patients. Therefore, nothing can be said in this regard. The magnitude of benefit observed should be calibrated in the presence of new variants and vaccination status.

Several conclusions could be drawn from the results of this systematic review. First, the sudden appearance of the COVID-19 epidemic led to a (justified) race to find effective treatments as quickly as possible. However, this research race was often erratic and lacked clear objectives. Given this situation, the ideal would have been the creation of a scientific committee (perhaps led by the World Health Organization) that would have established clear guidelines from the beginning and coordinated clinical research with pharmaceutical companies and national and international health organizations, as well as with medical societies. This would be, in our opinion, the direction that should be followed in possible future pandemics.

## Data Availability

The original contributions presented in the study are included in the article/Supplementary Material, further inquiries can be directed to the corresponding author.

## References

[B1] Abbas A. Abbas F. Abbas M. Abbasi S. Abbass H. (2022). Casirivimab and imdevimab in patients admitted to hospital with COVID-19 (RECOVERY): a randomised, controlled, open-label, platform trial. Lancet 399, 665–676. 10.1016/s0140-6736(22)00163-5 35151397 PMC8830904

[B2] Agencia Española de Medicamentos y Productos Sanitarios (AEMPS) (2022). Tratamientos disponibles para el manejo de la infección respiratoria por SARS-CoV-2. 19 de marzo de 2020. Available at: https://www.aemps.gob.es/laAEMPS/docs/medicamentos-disponibles-SARS-CoV-2-19-3-2020.pdf.

[B3] Albuquerque A. M. Eckert I. Tramujas L. Butler-Laporte G. McDonald E. G. Brophy J. M. (2023). Effect of tocilizumab, sarilumab, and baricitinib on mortality among patients hospitalized for COVID-19 treated with corticosteroids: a systematic review and meta-analysis. Clin. Microbiol. Infect. 29, 13–21. 10.1016/j.cmi.2022.07.008 35863630 PMC9293401

[B4] Amani B. Amani B. (2022). Efficacy and safety of sotrovimab in patients with COVID-19: a rapid review and meta-analysis. Rev. Med. Virol. 32, e2402. 10.1002/rmv.2402 36226323 PMC9874927

[B5] Annane D. Heming N. Grimaldi-Bensouda L. Frémeaux-Bacchi V. Vigan M. Roux A. L. (2020). Eculizumab as an emergency treatment for adult patients with severe COVID-19 in the intensive care unit: a proof-of-concept study. EClinicalMedicine 28, 100590. 10.1016/j.eclinm.2020.100590 33173853 PMC7644240

[B6] Barkas F. Filippas-Ntekouan S. Kosmidou M. Liberopoulos E. Liontos A. Milionis H. (2021). Anakinra in hospitalized non-intubated patients with coronavirus disease 2019: a Systematic review and meta-analysis. Rheumatol. Oxf. 60, 5527–5537. 10.1093/rheumatology/keab447 PMC8194671 33999135

[B7] Bausch K. Cartwright R. (2021). Evidence-based urology: when is a study or meta-analysis big enough? Eur. Urol. Focus 7, 1240–1242. 10.1016/j.euf.2021.09.021 34688587

[B8] Beigel J. H. Tomashek K. M. Dodd L. E. Mehta A. K. Zingman B. S. Kalil A. C. (2020). Remdesivir for the treatment of covid-19 - final report. N. Engl. J. Med. 383, 1813–1826. 10.1056/NEJMoa2007764 32445440 PMC7262788

[B9] Bekemv 300 mg concentrado para solución para perfusión (2021). Eculizumab. Ficha técnica. Available at: https://botplusweb.farmaceuticos.com//Documentos/AEMPS/FichasTecnicas/406524/FT_1231727001_1.pdf.

[B10] Bosdriesz J. R. Stel V. S. van Diepen M. Meuleman Y. Dekker F. W. Zoccali C. (2020). Evidence-based medicine-When observational studies are better than randomized controlled trials. Nephrol. Carlt. 25, 737–743. 10.1111/nep.13742 PMC7540602 32542836

[B11] Burwick R. M. Dellapiana G. Newman R. A. Smithson S. D. Naqvi M. Williams J. 3rd (2022). Complement blockade with eculizumab for treatment of severe Coronavirus Disease 2019 in pregnancy: a case series. Am. J. Reprod. Immunol. 88, e13559. 10.1111/aji.13559 35514201 PMC9347938

[B12] CEDER. Center for Drug Evaluation and Research (CDER) Review (2021). Emergency use authorization (EUA) for casirivimab and imdevimab. Available at: https://www.fda.gov/media/151863/download.

[B13] Celltrion use of regdanvimab for the treatment of COVID-19 (2021). Assessment report. INN/active substance: regdanvimab. Procedure number: EMEA/H/A-5(3)/1505. Committee for Medicinal Products for Human Use (CHMP). European Medicines Agency. Available at: https://www.ema.europa.eu/en/documents/referral/regdanvimab-treatment-covid-19-celltrion-covid-19-article-53-procedure-assessment-report_en.pdf.

[B14] Cheema H. A. Jafar U. Sohail A. Shahid A. Sahra S. Ehsan M. (2023). Nirmatrelvir-ritonavir for the treatment of COVID-19 patients: a systematic review and meta-analysis. J. Med. Virol. 95, e28471. 10.1002/jmv.28471 36606609

[B15] Chen H. Cohen P. Chen S. (2010). How big is a big odds ratio? Interpreting the magnitudes of odds ratios in epidemiological studies. Comm. Stat—Simul Comput. 39, 860–864. 10.1080/03610911003650383

[B16] Chen H. Zhang Z. Wang L. Huang Z. Gong F. Li X. (2020). First clinical study using HCV protease inhibitor danoprevir to treat COVID-19 patients. Med. Baltim. 99, e23357. 10.1097/MD.0000000000023357 PMC7710192 33235105

[B17] Cochrane Handbook for systematic reviews of interventions (2022). Available at: https://training.cochrane.org/handbook/current.

[B18] Coutant F. Touret F. Pin J. J. Alonzo M. Baronti C. Munier S. (2024). Neutralizing and enhancing monoclonal antibodies in SARS-CoV-2 convalescent patients: lessons from early variant infection and impact on shaping emerging variants. Emerg. Microbes Infect. 13, 2307510. 10.1080/22221751.2024.2307510 38240255 PMC10829827

[B19] CovidCAREgroup (2022). FDA monoclonal antibody guideline changes. Available at: https://www.covidcaregroup.org/blog/fda-monoclonal-antibodyguideline-changes.

[B20] Deng J. Heybati K. Ramaraju H. B. Zhou F. Rayner D. Heybati S. (2023). Differential efficacy and safety of anti-SARS-CoV-2 antibody therapies for the management of COVID-19: a systematic review and network meta-analysis. Infection 51, 21–35. 10.1007/s15010-022-01825-8 35438413 PMC9016212

[B21] Diurno F. Numis F. G. Porta G. Cirillo F. Maddaluno S. Ragozzino A. (2020). Eculizumab treatment in patients with COVID-19: preliminary results from real life ASL Napoli 2 Nord experience. Eur. Rev. Med. Pharmacol. Sci. 24, 4040–4047. 10.26355/eurrev_202004_20875 32329881

[B22] EUnetHTA (2021). “Rolling collaborative review” of covid-19 treatments. APN01 for the treatment of COVID-19- project ID: RCR09. Monitoring report. Available at: https://www.eunethta.eu/wp-content/uploads/2021/05/EUnetHTA-Covid-19_RCR09_APN01_V6.0.pdf.

[B23] European Medicines Agency (EMA) COVID-19 treatments (2025). Available at: https://www.ema.europa.eu/en/human-regulatory/overview/public-health-threats/coronavirus-disease-covid-19/treatments-vaccines/covid-19-treatments.

[B24] Evusheld (2019). Assessment report. International non-proprietary name: tixagevimab/cilgavimab. Proced. No. EMEA/H/C/005788/II/0001. Comm. Med. Prod. Hum. Use (CHMP). Available at: https://www.ema.europa.eu/en/documents/variation-report/evusheld-epar-assessment-report-variation_en.pdf.

[B25] Gao M. Ao G. Hao X. Xie B. (2023). Casirivimab-imdevimab treatment is associated with reduced rates of mortality and hospitalization in patients with COVID-19: a systematic review with meta-analysis. J. Infect. 87, 82–84. 10.1016/j.jinf.2023.04.019 37146726 PMC10155463

[B26] Goldman J. D. Lye D. C. B. Hui D. S. Marks K. M. Bruno R. Montejano R. (2020). Remdesivir for 5 or 10 Days in patients with severe covid-19. N. Engl. J. Med. 383, 1827–1837. 10.1056/NEJMoa2015301 32459919 PMC7377062

[B27] Gottlieb R. L. Vaca C. E. Paredes R. Mera J. Webb B. J. Perez G. (2022). Early remdesivir to prevent progression to severe covid-19 in outpatients. N. Engl. J. Med. 386, 305–315. 10.1056/NEJMoa2116846 34937145 PMC8757570

[B28] Grau S. Miró J. M. Olalla J. Alcalá J. C. Castro A. Rubio-Rodríguez D. (2023). Comparison of the design and methodology of Phase 3 clinical trials of bictegravir/emtricitabine/tenofovir alafenamide (BIC/FTC/TAF) and dolutegravir-based dual therapy (DTG) in HIV: a systematic review of the literature. Expert Rev. Anti Infect. Ther. 21, 65–76. 10.1080/14787210.2023.2149490 36399521

[B29] Gupta A. Gonzalez-Rojas Y. Juarez E. Crespo Casal M. Moya J. Falci D. R. (2021). Early treatment for covid-19 with SARS-CoV-2 neutralizing antibody sotrovimab. N. Engl. J. Med. 385, 1941–1950. 10.1056/NEJMoa2107934 34706189

[B30] Hammond J. Leister-Tebbe H. Gardner A. Abreu P. Bao W. Wisemandle W. (2022). Oral nirmatrelvir for high-risk, nonhospitalized adults with covid-19. N. Engl. J. Med. 386, 1397–1408. 10.1056/NEJMoa2118542 35172054 PMC8908851

[B31] Higgins J. P. T. Green S. (2011). The Cochrane collaboration. Cochrane Handb. Syst. Rev. Interventions. Available at: http://training.cochrane.org/handbook.

[B32] Higgins J. P. T. Thompson S. G. Deeks J. Altman D. G. (2003). Measuring inconsistency in meta-analyses. BMJ 327, 557–560. 10.1136/bmj.327.7414.557 12958120 PMC192859

[B33] Huang C. Lu T. L. Lin L. (2023). Remdesivir treatment lacks the effect on mortality reduction in hospitalized adult COVID-19 patients who required high-flow supplemental oxygen or invasive mechanical ventilation. Med. Kaunas. 59, 1027. 10.3390/medicina59061027 PMC10301784 37374231

[B34] Huang C. Wang Y. Li X. Ren L. Zhao J. Hu Y. (2020). Clinical features of patients infected with 2019 novel coronavirus in Wuhan, China. Lancet 395, 497–506. 10.1016/S0140-6736(20)30183-5 31986264 PMC7159299

[B35] Kim M. S. An M. H. Kim W. J. Hwang T. H. (2020). Comparative efficacy and safety of pharmacological interventions for the treatment of COVID-19: a systematic review and network meta-analysis. PLoS Med. 17, e1003501. 10.1371/journal.pmed.1003501 33378357 PMC7794037

[B36] Kimachi M. Onishi A. Tajika A. Kimachi K. Furukawa T. A. (2021). Systematic differences in effect estimates between observational studies and randomized control trials in meta-analyses in nephrology. Sci. Rep. 11, 6088. 10.1038/s41598-021-85519-5 33731727 PMC7971062

[B37] Kyriazopoulou E. Poulakou G. Milionis H. Metallidis S. Adamis G. Tsiakos K. (2021). Early treatment of COVID-19 with anakinra guided by soluble urokinase plasminogen receptor plasma levels: a double-blind, randomized controlled phase 3 trial. Nat. Med. 27, 1752–1760. 10.1038/s41591-021-01499-z 34480127 PMC8516650

[B38] Liberati A. Altman D. G. Tetzlaff J. Mulrow C. Gøtzsche P. C. Ioannidis J. P. (2009). The PRISMA statement for reporting systematic reviews and meta-analyses of studies that evaluate health care interventions: explanation and elaboration. PLoS Med. 6, e1000100. 10.1371/journal.pmed.1000100 19621070 PMC2707010

[B39] Liu W. Zhou P. Chen K. Ye Z. Liu F. Li X. (2020). Efficacy and safety of antiviral treatment for COVID-19 from evidence in studies of SARS-CoV-2 and other acute viral infections: a systematic review and meta-analysis. CMAJ 192 (27), E734-E744–E744. 10.1503/cmaj.200647 32493740 PMC7828899

[B40] Miao M. Jing X. De Clercq E. Li G. (2020). Danoprevir for the treatment of hepatitis C virus infection: design, development, and place in therapy. Drug Des. Devel Ther. 14, 2759–2774. 10.2147/DDDT.S254754 PMC7368560 32764876

[B41] Moher D. Liberati A. Tetzlaff J. Altman D. G. PRISMA Group (2009). Preferred reporting items for systematic reviews and meta-analyses: the PRISMA statement. Ann. Intern Med. 151, 264–W64. 10.7326/0003-4819-151-4-200908180-00135 19622511

[B42] Mozaffari E. Chandak A. Gottlieb R. L. Chima-Melton C. Read S. H. Lee E. (2023). Remdesivir is associated with reduced mortality in COVID-19 patients requiring supplemental oxygen including invasive mechanical ventilation across SARS-CoV-2 variants. Open Forum Infect. Dis. 10, ofad482. 10.1093/ofid/ofad482 37869410 PMC10588622

[B43] National Heart, Lung and Blood Institute (2020). NIH. Quality assessment of systematic reviews and meta-analyses. Available at: https://www.nhlbi.nih.gov/health-topics/study-quality-assessment-tools.

[B44] COVID-19 (APN01-COVID-19) (2024). Recombinant human angiotensin-converting enzyme 2 (rhACE2) as a treatment for patients with COVID-19 (APN01-COVID-19). Available at: https://clinicaltrials.gov/study/NCT04335136?intr=APN01&cond=Covid19&rank=1&tab=results.

[B45] COVID-19 (2023). Study to evaluate the efficacy and safety of leronlimab for mild to moderate COVID-19. Available at: https://clinicaltrials.gov/study/NCT04343651?cond=Covid19&intr=Leronlimab&rank=1.

[B46] Page M. J. Moher D. Bossuyt P. M. Boutron I. Hoffmann T. C. Mulrow C. D. (2021). PRISMA 2020 explanation and elaboration: updated guidance and exemplars for reporting systematic reviews. BMJ 372, n160. 10.1136/bmj.n160 33781993 PMC8005925

[B47] Pasin L. Cavalli G. Navalesi P. Sella N. Landoni G. Yavorovskiy A. G. (2021). Anakinra for patients with COVID-19: a meta-analysis of non-randomized cohort studies. Eur. J. Intern Med. 86, 34–40. 10.1016/j.ejim.2021.01.016 33581979 PMC7862887

[B48] Peng J. Fu M. Mei H. Zheng H. Liang G. She X. (2022). Efficacy and secondary infection risk of tocilizumab, sarilumab and anakinra in COVID-19 patients: a systematic review and meta-analysis. Rev. Med. Virol. 32, e2295. 10.1002/rmv.2295 34558756 PMC8646369

[B49] Ranstam J. Wagner P. (2022). Systematic reviews, meta-analyses, randomized trials, and observational studies. Acta Orthop. 93, 1–2. 10.1080/17453674.2021.1975398 34505797 PMC8788670

[B50] RECOVERY Collaborative Group (2022). Casirivimab and imdevimab in patients admitted to hospital with COVID-19 (RECOVERY): a randomised, controlled, open-label, platform trial. Lancet 399 (10325), , 665–676. 10.1016/S0140-6736(22)00163-5 35151397 PMC8830904

[B51] Ronapreve (2024). Ronapreve 300 mg + 300 mg solución inyectable y para perfusión. Available at: https://ec.europa.eu/health/documents/community-register/2021/20211112154000/anx_154000_es.pdf.

[B52] Rosas I. O. Bräu N. Waters M. Go R. C. Hunter B. D. Bhagani S. (2021). Tocilizumab in hospitalized patients with severe covid-19 pneumonia. N. Engl. J. Med. 384, 1503–1516. 10.1056/NEJMoa2028700 33631066 PMC7953459

[B53] Rubio T. C. (1996). Diseño estadístico de ensayos clínicos [Statistical design in clinical trials]. Med. Clin. Barc. 107, 303–309. 8965495

[B54] Rubio-Rivas M. Forero C. G. Mora-Luján J. M. Montero A. Formiga F. Homs N. A. (2021). Beneficial and harmful outcomes of tocilizumab in severe COVID-19: a systematic review and meta-analysis. Pharmacotherapy 41, 884–906. 10.1002/phar.2627 34558742 PMC8661749

[B55] Rubio-Rodríguez D. De Diego Blanco S. Pérez M. Rubio-Terrés C. (2017). Cost-effectiveness of drug treatments for advanced melanoma: a systematic literature review. Pharmacoeconomics 35, 879–893. 10.1007/s40273-017-0517-1 28551858

[B56] Ruggenenti P. Di Marco F. Cortinovis M. Lorini L. Sala S. Novelli L. (2021). Eculizumab in patients with severe coronavirus disease 2019 (COVID-19) requiring continuous positive airway pressure ventilator support: retrospective cohort study. PLoS One 16, e0261113. 10.1371/journal.pone.0261113 34928990 PMC8687582

[B57] SCImago Journal Rank (SJR) (2023). Research Impact and Scholarly Metrics. Understanding and using bibliometrics to evaluate journals and impact of scholarly work. Available at: https://libguides.daemen.edu/c.php?g=1239513&p=9072137#:∼:text=A%20journal%20with%20a%20SJR,has%20below%20average%20citation%20potential.

[B58] Shankar-Hari M. Marshall J. Murthy S. Diaz J. Vale C. Tierney J. (2021). On Behalf of the WHO Covid- Clinical Management Characterization Working Group. Anti-Interleukin-6 therapies for hospitalised patients with COVID-19: a protocol for a prospective meta-analysis of randomised trials. Available at: https://www.crd.york.ac.uk/PROSPEROFILES/230155_PROTOCOL_20210329.pdf (access: (access: 14/May/2023).

[B59] Shrier I. Boivin J. F. Steele R. J. Platt R. W. Furlan A. Kakuma R. (2007). Should meta-analyses of interventions include observational studies in addition to randomized controlled trials? A critical examination of underlying principles. Am. J. Epidemiol. 166, 1203–1209. 10.1093/aje/kwm189 17712019

[B60] Sigman M. (2011). A meta-analysis of meta-analyses. Fertil. Steril. 96, 11–14. 10.1016/j.fertnstert.2011.05.029 21723440

[B61] Toews I. Anglemyer A. Nyirenda J. L. Alsaid D. Balduzzi S. Grummich K. (2024). Healthcare outcomes assessed with observational study designs compared with those assessed in randomized trials: a meta-epidemiological study. Cochrane Database Syst. Rev. 1, MR000034. 10.1002/14651858.MR000034.pub3 38174786 PMC10765475

[B62] Wang Y. Zheng J. Zhu K. Xu C. Wang D. Hou M. (2023). The effect of tixagevimab-cilgavimab on clinical outcomes in patients with COVID-19: a systematic review with meta-analysis. J. Infect. 86, e15–e17. 10.1016/j.jinf.2022.08.021 36031156 PMC9420004

[B63] Xevudy (2021). International non-proprietary name: sotrovimab. CHMP assessment report. Procedure No. EMEA/H/C/005676/0000. Committee for Medicinal Products for Human Use (CHMP). European Medicines Agency. Available at: https://www.ema.europa.eu/en/documents/assessment-report/xevudy-epar-public-assessment-report_en.pdf.

[B64] Yang B. Fulcher J. A. Ahn J. Berro M. Goodman-Meza D. Dhody K. (2021). Clinical characteristics and outcomes of coronavirus disease 2019 patients who received compassionate-use leronlimab. Clin. Infect. Dis. 73, e4082–e4089. 10.1093/cid/ciaa1583 33079180 PMC7665416

[B65] Yang M. Li A. Jiang L. Wang Y. Tran C. Ao G. (2022). Regdanvimab improves disease mortality and morbidity in patients with COVID-19: a meta-analysis. J. Infect. 85, e122–e124. 10.1016/j.jinf.2022.05.044 35728643 PMC9212623

