# Supplementary material for: Effectiveness of pharmacological treatments for COVID-19 due to SARS-CoV-2: a systematic literature review
Source: Front Pharmacol. 2025 Feb 28;16:1469681. doi: 10.3389/fphar.2025.1469681 (PMC11907208; doi:10.3389/fphar.2025.1469681)
Supplement: Supplementary file 1 [file DataSheet1.pdf]

# Effectiveness of pharmacological treatments for COVID-19 due to SARS-CoV-2: a systematic literature review

## SUPPLEMENTS

## SUPPLEMENT 1A. PUBMED SEARCH STRATEGY

**ARBIDOL**

Search: arbidol covid-19 Filters: Meta-Analysis Sort by: Most Recent (("umifenovir"[Supplementary Concept] OR "umifenovir"[All Fields] OR "arbidol"[All Fields] OR "arbidole"[All Fields]) AND ("covid 19"[All Fields] OR "covid 19"[MeSH Terms] OR "covid 19 vaccines"[All Fields] OR "covid 19 vaccines"[MeSH Terms] OR "covid 19 serotherapy"[All Fields] OR "covid 19 nucleic acid testing"[All Fields] OR "covid 19 nucleic acid testing"[MeSH Terms] OR "covid 19 serological testing"[All Fields] OR "covid 19 serological testing"[MeSH Terms] OR "covid 19 testing"[All Fields] OR "covid 19 testing"[MeSH Terms] OR "sars cov 2"[All Fields] OR "sars cov 2"[MeSH Terms] OR "severe acute respiratory syndrome coronavirus 2"[All Fields] OR "ncov"[All Fields] OR "2019 ncov"[All Fields] OR (("coronavirus"[MeSH Terms] OR "coronavirus"[All Fields] OR "cov"[All Fields]) AND 2019/11/01:3000/12/31[Date - Publication]))) AND (meta-analysis[Filter])

**FAVIPIRAVIR**

Search: favipiravir covid 19 Filters: Meta-Analysis Sort by: Most Recent (("favipiravir"[Supplementary Concept] OR "favipiravir"[All Fields]) AND ("covid 19"[All Fields] OR "covid 19"[MeSH Terms] OR "covid 19 vaccines"[All Fields] OR "covid 19 vaccines"[MeSH Terms] OR "covid 19 serotherapy"[All Fields] OR "covid 19 nucleic acid testing"[All Fields] OR "covid 19 nucleic acid testing"[MeSH Terms] OR "covid 19 serological testing"[All Fields] OR "covid 19 serological testing"[MeSH Terms] OR "covid 19 testing"[All Fields] OR "covid 19 testing"[MeSH Terms] OR "sars cov 2"[All Fields] OR "sars cov 2"[MeSH Terms] OR "severe acute respiratory syndrome coronavirus 2"[All Fields] OR "ncov"[All Fields] OR "2019 ncov"[All Fields] OR (("coronavirus"[MeSH Terms] OR "coronavirus"[All Fields] OR "cov"[All Fields]) AND 2019/11/01:3000/12/31[Date - Publication]))) AND (meta-analysis[Filter])

**HIDROXICLOROQUINA-CLOROQUINA**

Search: hydroxychloroquine covid-19 Filters: Meta-Analysis Sort by: Most Recent (("hydroxychloroquine"[MeSH Terms] OR "hydroxychloroquine"[All Fields]) AND ("covid 19"[All Fields] OR "covid 19"[MeSH Terms] OR "covid 19 vaccines"[All Fields] OR "covid 19 vaccines"[MeSH Terms] OR "covid 19 serotherapy"[All Fields] OR "covid 19 nucleic acid testing"[All Fields] OR "covid 19 nucleic acid testing"[MeSH Terms] OR "covid 19 serological testing"[All Fields] OR "covid 19 serological testing"[MeSH Terms] OR "covid 19 testing"[All Fields] OR "covid 19 testing"[MeSH Terms] OR "sars cov 2"[All Fields] OR "sars cov 2"[MeSH Terms] OR "severe acute respiratory syndrome coronavirus 2"[All Fields] OR "ncov"[All Fields] OR "2019 ncov"[All Fields] OR (("coronavirus"[MeSH Terms] OR "coronavirus"[All Fields] OR "cov"[All Fields]) AND 2019/11/01:3000/12/31[Date - Publication]))) AND (meta-analysis[Filter])

**INTERFERON**

Search: interferon covid 19 Filters: Meta-Analysis Sort by: Most Recent (("interferon s"[All Fields] OR "interferone"[All Fields] OR "interferones"[All Fields] OR "interferons"[MeSH Terms] OR "interferons"[All Fields] OR "interferon"[All Fields]) AND ("covid 19"[All Fields] OR "covid 19"[MeSH Terms] OR "covid 19 vaccines"[All Fields] OR "covid 19 vaccines"[MeSH Terms] OR "covid 19 serotherapy"[All Fields] OR "covid 19 nucleic acid testing"[All Fields] OR "covid 19 nucleic acid testing"[MeSH Terms] OR "covid 19 serological testing"[All Fields] OR "covid 19 serological testing"[MeSH Terms] OR "covid 19 testing"[All Fields] OR "covid 19 testing"[MeSH Terms] OR "sars cov 2"[All Fields] OR "sars cov 2"[MeSH Terms] OR "severe acute respiratory syndrome coronavirus 2"[All Fields] OR "ncov"[All Fields] OR "2019 ncov"[All Fields] OR (("coronavirus"[MeSH Terms] OR "coronavirus"[All Fields] OR "cov"[All Fields]) AND 2019/11/01:3000/12/31[Date - Publication]))) AND (meta-analysis[Filter])

**LOPINAVIR-RITONAVIR**

Search: lopinavir ritonavir covid-19 Filters: Meta-Analysis Sort by: Most Recent (("lopinavir"[MeSH Terms] OR "lopinavir"[All Fields]) AND ("ritonavir"[MeSH Terms] OR "ritonavir"[All Fields]) AND ("covid 19"[All Fields] OR "covid 19"[MeSH Terms] OR "covid 19 vaccines"[All Fields] OR "covid 19 vaccines"[MeSH Terms] OR "covid 19 serotherapy"[All Fields] OR "covid 19 nucleic acid testing"[All Fields] OR "covid 19 nucleic acid testing"[MeSH Terms] OR "covid 19 serological testing"[All Fields] OR "covid 19 serological testing"[MeSH Terms] OR "covid 19 testing"[All Fields] OR "covid 19 testing"[MeSH Terms] OR "sars cov 2"[All Fields] OR "sars cov 2"[MeSH Terms] OR "severe acute respiratory syndrome coronavirus 2"[All Fields] OR "ncov"[All Fields] OR "2019 ncov"[All Fields] OR (("coronavirus"[MeSH Terms] OR "coronavirus"[All Fields] OR "cov"[All Fields]) AND 2019/11/01:3000/12/31[Date - Publication]))) AND (meta-analysis[Filter])

**REMDESIVIR**

Search: remdesivir covid 19 Filters: Meta-Analysis Sort by: Most Recent (("remdesivir"[Supplementary Concept] OR "remdesivir"[All Fields]) AND ("covid 19"[All Fields] OR "covid 19"[MeSH Terms] OR "covid 19 vaccines"[All Fields] OR "covid 19 vaccines"[MeSH Terms] OR "covid 19 serotherapy"[All Fields] OR "covid 19 nucleic acid testing"[All Fields] OR "covid 19 nucleic acid testing"[MeSH Terms] OR "covid 19 serological testing"[All Fields] OR "covid 19 serological testing"[MeSH Terms] OR "covid 19 testing"[All Fields] OR "covid 19 testing"[MeSH Terms] OR "sars cov 2"[All Fields] OR "sars cov 2"[MeSH Terms] OR "severe acute respiratory syndrome coronavirus 2"[All Fields] OR "ncov"[All Fields] OR "2019 ncov"[All Fields] OR (("coronavirus"[MeSH Terms] OR "coronavirus"[All Fields] OR "cov"[All Fields]) AND 2019/11/01:3000/12/31[Date - Publication]))) AND (meta-analysis[Filter])

**SARILUMAB**

Search: sarilumab covid 19 Filters: Meta-Analysis Sort by: Most Recent (("sarilumab"[Supplementary Concept] OR "sarilumab"[All Fields]) AND ("covid 19"[All Fields] OR "covid 19"[MeSH Terms] OR "covid 19 vaccines"[All Fields] OR "covid 19 vaccines"[MeSH Terms] OR "covid 19 serotherapy"[All Fields] OR

"covid 19 nucleic acid testing"[All Fields] OR "covid 19 nucleic acid testing"[MeSH Terms] OR "covid 19 serological testing"[All Fields] OR "covid 19 serological testing"[MeSH Terms] OR "covid 19 testing"[All Fields] OR "covid 19 testing"[MeSH Terms] OR "sars cov 2"[All Fields] OR "sars cov 2"[MeSH Terms] OR "severe acute respiratory syndrome coronavirus 2"[All Fields] OR "ncov"[All Fields] OR "2019 ncov"[All Fields] OR (("coronavirus"[MeSH Terms] OR "coronavirus"[All Fields] OR "cov"[All Fields]) AND 2019/11/01:3000/12/31[Date - Publication]))) AND (meta-analysis[Filter])

**TOCILIZUMAB**
Search: tocilizumab covid 19 Filters: Meta-Analysis Sort by: Most Recent (("tocilizumab"[Supplementary Concept] OR "tocilizumab"[All Fields]) AND ("covid 19"[All Fields] OR "covid 19"[MeSH Terms] OR "covid 19 vaccines"[All Fields] OR "covid 19 vaccines"[MeSH Terms] OR "covid 19 serotherapy"[All Fields] OR "covid 19 nucleic acid testing"[All Fields] OR "covid 19 nucleic acid testing"[MeSH Terms] OR "covid 19 serological testing"[All Fields] OR "covid 19 serological testing"[MeSH Terms] OR "covid 19 testing"[All Fields] OR "covid 19 testing"[MeSH Terms] OR "sars cov 2"[All Fields] OR "sars cov 2"[MeSH Terms] OR "severe acute respiratory syndrome coronavirus 2"[All Fields] OR "ncov"[All Fields] OR "2019 ncov"[All Fields] OR (("coronavirus"[MeSH Terms] OR "coronavirus"[All Fields] OR "cov"[All Fields]) AND 2019/11/01:3000/12/31[Date - Publication]))) AND (meta-analysis[Filter])

**WITHOUT RESULTS (strategies with the same structure)**
APN01
CAMRELIZUMAB
DANOPREVIR
DARUNAVIR-COLICISTAT
ECULIZUMAB
LERONLIMAB
REGN 3048
TIMOSINA ALFA 1 (TIMALFASINA)

**SUPPLEMENT 1B. COCHRANE LIBRARY STRATEGY (https://www.cochranelibrary.com/)**

Search: ARBIDOL, FAVIPIRAVIR, HIDROXICLOROQUINA-CLOROQUINA, INTERFERON, LOPINAVIR-RITONAVIR, REMDESIVIR, SARILUMAB, TOCILIZUMAB, APN01, CAMRELIZUMAB, DANOPREVIR, DARUNAVIR-COLOSTAT, ECULIZUMAB, LERONLIMAB, REGN 3048, TIMISINA ALFA 1 (TIMALFASINA).

**SUPPLEMENT 1C. EUROPEAN MEDICINES AGENCY (https://www.ema.europa.eu/en/medicines)**

Search: ARBIDOL, FAVIPIRAVIR, HIDROXICLOROQUINA-CLOROQUINA, INTERFERON, LOPINAVIR-RITONAVIR, REMDESIVIR, SARILUMAB, TOCILIZUMAB, APN01, CAMRELIZUMAB, DANOPREVIR, DARUNAVIR-COLOSTAT, ECULIZUMAB, LERONLIMAB, REGN 3048, TIMISINA ALFA 1 (TIMALFASINA).

**SUPPLEMENT 1D. AGENCIA ESPAÑOLA DE MEDICAMENTOS Y PRODUCTOS SANITARIOS (https://www.aemps.gob.es/)**

Search: ARBIDOL, FAVIPIRAVIR, HIDROXICLOROQUINA-CLOROQUINA, INTERFERON, LOPINAVIR-RITONAVIR, REMDESIVIR, SARILUMAB, TOCILIZUMAB, APN01, CAMRELIZUMAB, DANOPREVIR, DARUNAVIR-COLOSTAT, ECULIZUMAB, LERONLIMAB, REGN 3048, TIMISINA ALFA 1 (TIMALFASINA).

**SUPPLEMENT 1E. GOOGLE (https://www.google.com/)**

Search: ARBIDOL, FAVIPIRAVIR, HIDROXICLOROQUINA-CLOROQUINA, INTERFERON, LOPINAVIR-RITONAVIR, REMDESIVIR, SARILUMAB, TOCILIZUMAB, APN01, CAMRELIZUMAB, DANOPREVIR, DARUNAVIR-COLOSTAT, ECULIZUMAB, LERONLIMAB, REGN 3048, TIMISINA ALFA 1 (TIMALFASINA).

Searches in COCHRANE LIBRARY, EUROPEAN MEDICINES AGENCY, AGENCIA ESPAÑOLA DE MEDICAMENTOS Y PRODUCTOS SANITARIOS and GOOGLE, were performed individually for each drug with the corresponding name, identifying - where appropriate - the corresponding meta-analyses.

**SUPPLEMENT 2. ARTICLES INCLUDED IN THE SYNTHESIS (alphabetical order)**

ACTIV-3–Therapeutics for Inpatients with COVID-19 (TICO) Study Group. Tixagevimab-cilgavimab for treatment of patients hospitalised with COVID-19: a randomised, double-blind, phase 3 trial. Lancet Respir Med. 2022; 10: 972-84.

Albuquerque AM, Eckert I, Tramujas L, Butler-Laporte G, McDonald EG, Brophy JM, et al. Effect of tocilizumab, sarilumab, and baricitinib on mortality among patients hospitalized for COVID-19 treated with corticosteroids: a systematic review and meta-analysis. Clin Microbiol Infect. 2023; 29: 13-21.

Alkofide H, Almohaizeie A, Almuhaini S, Alotaibi B, Alkharfy KM. Tocilizumab and Systemic Corticosteroids in the Management of Patients with COVID-19: A Systematic Review and Meta-Analysis. Int J Infect Dis. 2021; 110: 320-9.

Amani B, Khanijahani A, Amani B. Hydroxychloroquine plus standard of care compared with standard of care alone in COVID-19: a meta-analysis of randomized controlled trials. Sci Rep. 2021; 11: 11974.

Amani B, Amani B. Efficacy and safety of sotrovimab in patients with COVID-19: A rapid review and meta-analysis. Rev Med Virol. 2022; 32: e2402.

Amani B, Amani B. Efficacy and safety of nirmatrelvir/ritonavir (Paxlovid) for COVID-19: A rapid review and meta-analysis. J Med Virol. 2023; 95: e28441.

Amani B, Amani B. Efficacy and safety of regdanvimab in patients with mild to moderate COVID-19: A rapid review and meta-analysis. Br J Clin Pharmacol. 2023; 89: 1282-90.

Amstutz A, Speich B, Mentré F, Rueegg CS, Belhadi D, Assoumou L, et al. Effects of remdesivir in patients hospitalised with COVID-19: a systematic review and individual patient data meta-analysis of randomised controlled trials. Lancet Respir Med. 2023; 11: 453-64.

Angamo MT, Mohammed MA, Peterson GM. Efficacy and safety of remdesivir in hospitalised COVID-19 patients: a systematic review and meta-analysis. Infection. 2022; 50: 27-41.

Ao G, Li T, Wang Y, Tran C, Qi X. Lack of efficacy for sotrovimab use in patients with COVID-19: A meta-analysis. J Infect. 2022; 85: e10-2.

Axfors C, Schmitt AM, Janiaud P, Van't Hooft J, Abd-Elsalam S, Abdo EF, et al. Mortality outcomes with hydroxychloroquine and chloroquine in COVID-19 from an international collaborative meta-analysis of randomized trials. Nat Commun. 2021; 12: 2349.

Aziz M, Haghbin H, Abu Sitta E, Nawras Y, Fatima R, Sharma S, et al. Efficacy of tocilizumab in COVID-19: A systematic review and meta-analysis. J Med Virol. 2021; 93: 1620-30.

Beigel JH, Tomashek KM, Dodd LE, Mehta AK, Zingman BS, Kalil AC, et al; ACTT-1 Study Group Members. Remdesivir for the Treatment of Covid-19 - Final Report. N Engl J Med. 2020; 383: 1813-26.

Barkas F, Filippas-Ntekouan S, Kosmidou M, Liberopoulos E, Liontos A, Milionis H. Anakinra in hospitalized non-intubated patients with coronavirus disease 2019: a Systematic review and meta-analysis. Rheumatology (Oxford). 2021; 60: 5527-37.

Beckerman R, Gori A, Jeyakumar S, Malin JJ, Paredes R, Póvoa P, et al. Remdesivir for the treatment of patients hospitalized with COVID-19 receiving supplemental oxygen: a targeted literature review and meta-analysis. Sci Rep. 2022; 12: 9622.

Berardicurti O, Ruscitti P, Ursini F, D'Andrea S, Ciaffi J, Meliconi R, et al. Mortality in tocilizumab-treated patients with COVID-19: a systematic review and meta-analysis. Clin Exp Rheumatol. 2020; 38: 1247-54.

Buchynskyi M, Kamyshna I, Lyubomirskaya K, Moshynets O, Kobyliak N, Oksenych V, et al. Efficacy of interferon alpha for the treatment of hospitalized patients with COVID-19: A meta-analysis. Front Immunol. 2023; 14: 1069894.

CEDER. Center for Drug Evaluation and Research (CDER) Review. Emergency Use Authorization (EUA) for casirivimab and imdevimab. July 30, 2021. Disponible en URL: https://www.fda.gov/media/151863/download (acceso: 17/11/2023).

Celltrion use of regdanvimab for the treatment of COVID-19. Assessment report. INN/active substance: regdanvimab. Procedure number: EMEA/H/A-5(3)/1505. Committee for Medicinal Products for Human Use (CHMP). European Medicines Agency. EMA/192245/2021. 25 March 2021. Disponible en URL: https://www.ema.europa.eu/en/documents/referral/regdanvimab-treatment-covid-19-celltrion-covid-19-article-53-procedure-assessment-report_en.pdf (acceso: 17/11/2023).

Celotto S, Veronese N, Barbagallo M, Ometto F, Smith L, Pardhan S, et al. An umbrella review of systematic reviews with meta-analyses evaluating positive and negative outcomes of Hydroxychloroquine and chloroquine therapy. Int J Infect Dis. 2021; 103: 599-606.

Cheema HA, Jafar U, Sohail A, Shahid A, Sahra S, Ehsan M, et al. Nirmatrelvir-ritonavir for the treatment of COVID-19 patients: A systematic review and meta-analysis. J Med Virol. 2023; 95: e28471.

Chen CX, Hu F, Wei J, Yuan LT, Wen TM, Gale RP, et al. Systematic review and meta-analysis of tocilizumab in persons with coronavirus disease-2019 (COVID-19). Leukemia. 2021; 35: 1661-70.

Chen WC, Hsu CK, Chen CY, Lai CC, Hung SH, Lin WT. Clinical efficacy and safety of interferon-β-containing regimens in the treatment of patients with COVID-19: a systematic review and meta-analysis of randomized controlled trials. Expert Rev Anti Infect Ther. 2022; 20: 741-7.

Chi G, Memar Montazerin S, Lee JJ, Kazmi SHA, Shojaei F, Fitzgerald C, et al. Effect of azithromycin and hydroxychloroquine in patients hospitalized with COVID-19: Network meta-analysis of randomized controlled trials. J Med Virol. 2021; 93: 6737-49.

Chivese T, Musa OAH, Hindy G, Al-Wattary N, Badran S, Soliman N, et al. Efficacy of chloroquine and hydroxychloroquine in treating COVID-19 infection: A meta-review of systematic reviews and an updated meta-analysis. Travel Med Infect Dis. 2021; 43: 102135.

Dahms K, Mikolajewska A, Ansems K, Metzendorf MI, Benstoem C, Stegemann M. Anakinra for the treatment of COVID-19 patients: a systematic review and meta-analysis. Eur J Med Res. 2023; 28: 100.

Deng J, Heybati K, Ramaraju HB, Zhou F, Rayner D, Heybati S. Differential efficacy and safety of anti-SARS-CoV-2 antibody therapies for the management of COVID-19: a systematic review and network meta-analysis. Infection. 2023; 51: 21-35.

Elavarasi A, Prasad M, Seth T, Sahoo RK, Madan K, Nischal N, et al. Chloroquine and Hydroxychloroquine for the Treatment of COVID-19: a Systematic Review and Meta-analysis. J Gen Intern Med. 2020; 35: 3308-14.

Elsawah HK, Elsokary MA, Elrazzaz MG, Elshafie AH. Hydroxychloroquine for treatment of nonsevere COVID-19 patients: Systematic review and meta-analysis of controlled clinical trials. J Med Virol. 2021; 93: 1265-75.

Evusheld. Assessment report. International non-proprietary name: tixagevimab/cilgavimab. Procedure No. EMEA/H/C/005788/II/0001. Committee for Medicinal Products for Human Use (CHMP). European Medicines Agency. EMA/834036/2022. 15 September 2022. Disponible en URL: https://www.ema.europa.eu/en/documents/variation-report/evusheld-epar-assessment-report-variation_en.pdf (acceso: 17/11/2023).

Fiolet T, Guihur A, Rebeaud ME, Mulot M, Peiffer-Smadja N, Mahamat-Saleh Y. Effect of hydroxychloroquine with or without azithromycin on the mortality of coronavirus disease 2019 (COVID-19) patients: a systematic review and meta-analysis. Clin Microbiol Infect. 2021; 27: 19-27.

Gao M, Ao G, Hao X, Xie B. Casirivimab-imdevimab treatment is associated with reduced rates of mortality and hospitalization in patients with COVID-19: A systematic review with meta-analysis. J Infect. 2023; 87: 82-4.

García-Albéniz X, Del Amo J, Polo R, Morales-Asencio JM, Hernán MA. Systematic review and meta-analysis of randomized trials of hydroxychloroquine for the prevention of COVID-19. Eur J Epidemiol. 2022; 37: 789-96.

Ghazy RM, Almaghraby A, Shaaban R, Kamal A, Beshir H, Moursi A, et al. A systematic review and meta-analysis on chloroquine and hydroxychloroquine as monotherapy or combined with azithromycin in COVID-19 treatment. Sci Rep. 2020; 10: 22139.

Ghosn L, Chaimani A, Evrenoglou T, Davidson M, Graña C, Schmucker C, et al. Interleukin-6 blocking agents for treating COVID-19: a living systematic review. Cochrane Database of Systematic Reviews 2021, Issue 3. Art. No.: CD013881. DOI: 10.1002/14651858.CD013881.

Ghosn L, Assi R, Evrenoglou T, Buckley BS, Henschke N, Probyn K, et al. Interleukin-6 blocking agents for treating COVID-19: a living systematic review. Cochrane Database Syst Rev. 2023; 6: CD013881.

Goldman JD, Lye DCB, Hui DS, Marks KM, Bruno R, Montejano R, et al; GS-US-540-5773 Investigators. Remdesivir for 5 or 10 Days in Patients with Severe Covid-19. N Engl J Med. 2020; 383: 1827-37.

Gottlieb RL, Vaca CE, Paredes R, Mera J, Webb BJ, Perez G, et al; GS-US-540-9012 (PINETREE) Investigators. Early Remdesivir to Prevent Progression to Severe Covid-19 in Outpatients. N Engl J Med. 2022; 386: 305-15.

Grundeis F, Ansems K, Dahms K, Thieme V, Metzendorf M-I, Skoetz N, et al. Remdesivir for the treatment of COVID-19. Cochrane Database of Systematic Reviews 2023, Issue 1. Art. No.: CD014962. DOI: 10.1002/14651858.CD014962.pub2.

Gupta A, Gonzalez-Rojas Y, Juarez E, Crespo Casal M, Moya J, Falci DR, et al; COMET-ICE Investigators. Early Treatment for Covid-19 with SARS-CoV-2 Neutralizing Antibody Sotrovimab. N Engl J Med. 2021; 385: 1941-50.

Gupta T, Thakkar P, Kalra B, Kannan S. Hydroxychloroquine in the treatment of coronavirus disease 2019: Rapid updated systematic review and meta-analysis. Rev Med Virol. 2022; 32: e2276.

Hammond J, Leister-Tebbe H, Gardner A, Abreu P, Bao W, Wisemandle W, et al; EPIC-HR Investigators. Oral Nirmatrelvir for High-Risk, Nonhospitalized Adults with Covid-19. N Engl J Med. 2022; 386: 1397-1408.

Hassanipour S, Arab-Zozani M, Amani B, Heidarzad F, Fathalipour M, Martinez-de-Hoyo R. The efficacy and safety of Favipiravir in treatment of COVID-19: a systematic review and meta-analysis of clinical trials. Sci Rep. 2021; 11: 11022.

Huang D, Yu H, Wang T, Yang H, Yao R, Liang Z. Efficacy and safety of umifenovir for coronavirus disease 2019 (COVID-19): A systematic review and meta-analysis. J Med Virol. 2021; 93: 481-90.

Huang C, Lu TL, Lin L. Remdesivir Treatment Lacks the Effect on Mortality Reduction in Hospitalized Adult COVID-19 Patients Who Required High-Flow Supplemental Oxygen or Invasive Mechanical Ventilation. Medicina (Kaunas). 2023; 59: 1027.

Hung DT, Ghula S, Aziz JMA, Makram AM, Tawfik GM, Abozaid AA, et al. The efficacy and adverse effects of favipiravir on patients with COVID-19: A systematic review and meta-analysis of published clinical trials and observational studies. Int J Infect Dis. 2022; 120: 217-27.

Jiang Y, Chen D, Cai D, Yi Y, Jiang S. Effectiveness of remdesivir for the treatment of hospitalized COVID-19 persons: A network meta-analysis. J Med Virol. 2021; 93: 1171-4.

Kaka AS, MacDonald R, Linskens EJ, Langsetmo L, Vela K, Duan-Porter W, et al. Major Update 2: Remdesivir for Adults With COVID-19: A Living Systematic Review and Meta-analysis for the American College of Physicians Practice Points. Ann Intern Med. 2022; 175: 701-9.

Kashour Z, Riaz M, Garbati MA, AlDosary O, Tlayjeh H, Gerberi D, et al. Efficacy of chloroquine or hydroxychloroquine in COVID-19 patients: a systematic review and meta-analysis. J Antimicrob Chemother. 2021; 76: 30-42.

Kertes J, Shapiro Ben David S, Engel-Zohar N, Rosen K, Hemo B, Kantor A, et al. Association Between AZD7442 (Tixagevimab-Cilgavimab) Administration and Severe Acute Respiratory Syndrome Coronavirus 2 (SARS-CoV-2) Infection, Hospitalization, and Mortality. Clin Infect Dis. 2023; 76: e126-32.

Khan FA, Stewart I, Fabbri L, Moss S, Robinson K, Smyth AR, et al. Systematic review and meta-analysis of anakinra, sarilumab, siltuximab and tocilizumab for COVID-19. Thorax. 2021; 76: 907-19.

Kim MS, An MH, Kim WJ, Hwang TH. Comparative efficacy and safety of pharmacological interventions for the treatment of COVID-19: A systematic review and network meta-analysis. PLoS Med. 2020; 17: e1003501.

Kyriazopoulou E, Poulakou G, Milionis H, Metallidis S, Adamis G, Tsiakos K, et al. Early treatment of COVID-19 with anakinra guided by soluble urokinase plasminogen receptor plasma levels: a double-blind, randomized controlled phase 3 trial. Nat Med. 2021; 27: 1752-60.

Kyriakopoulos C, Ntritsos G, Gogali A, Milionis H, Evangelou E, Kostikas K. Tocilizumab administration for the treatment of hospitalized patients with COVID-19: A systematic review and meta-analysis. Respirology. 2021; 26: 1027-40.

Lai CC, Chen CH, Wang CY, Chen KH, Wang YH, Hsueh PR. Clinical efficacy and safety of remdesivir in patients with COVID-19: a systematic review and network meta-analysis of randomized controlled trials. J Antimicrob Chemother. 2021; 76: 1962-8.

Lan SH, Lai CC, Huang HT, Chang SP, Lu LC, Hsueh PR. Tocilizumab for severe COVID-19: a systematic review and meta-analysis. Int J Antimicrob Agents. 2020; 56: 106103.

Lee TC, Murthy S, Del Corpo O, Senécal J, Butler-Laporte G, Sohani ZN, et al. Remdesivir for the treatment of COVID-19: a systematic review and meta-analysis. Clin Microbiol Infect. 2022; 28: 1203-10.

Lin WT, Hung SH, Lai CC, Wang CY, Chen CH. The effect of tocilizumab on COVID-19 patient mortality: A systematic review and meta-analysis of randomized controlled trials. Int Immunopharmacol. 2021; 96: 107602.

Luo L, Luo T, Du M, Mei H, Hu Y. Efficacy and safety of tocilizumab in hospitalized COVID-19 patients: A systematic review and meta-analysis. J Infect. 2022; 84: 418-67.

Marx K, Gončarova K, Fedders D, Kalbitz S, Kellner N, Fedders M, et al. Clinical outcomes of hospitalized COVID-19 patients treated with remdesivir: a retrospective analysis of a large tertiary care center in Germany. Infection. 2023; 51: 97-108.

McConnell D, Harte M, Walsh C, Murphy D, Nichol A, Barry M, et al. Comparative effectiveness of neutralising monoclonal antibodies in high risk COVID-19 patients: a Bayesian network meta-analysis. Sci Rep. 2022; 12: 17561.

Metchurtchlishvili R, Chkhartishvili N, Abutidze A, Endeladze M, Ezugbaia M, Bakradze A, et al. Effect of remdesivir on mortality and the need for mechanical ventilation among hospitalized patients with COVID-19: real-world data from a resource-limited country. Int J Infect Dis. 2023; 129: 63-9.

Million M, Roussel Y, Gautret P, Raoult D. Effect of hydroxychloroquine and azithromycin on SARS-CoV-2 clearance in COVID-19 patients, a meta-analysis. Int J Antimicrob Agents. 2021; 57: 106240.

Moosazadeh M, Mousavi T. Combination therapy of tocilizumab and steroid for COVID-19 patients: A meta-analysis. J Med Virol. 2022; 94: 1350-6.

Mozaffari E, Chandak A, Gottlieb RL, Chima-Melton C, Read SH, Lee E, et al. Remdesivir Is Associated With Reduced Mortality in COVID-19 Patients Requiring Supplemental Oxygen Including Invasive Mechanical Ventilation Across SARS-CoV-2 Variants. Open Forum Infect Dis. 2023; 10: ofad482. (a)

Mozaffari E, Chandak A, Gottlieb RL, Chima-Melton C, Read SH, Jiang H, Chiang M, Lee E, Gupta R, Berry M, Kalil AC. Remdesivir reduced mortality in immunocompromised patients hospitalized for COVID-19 across variant waves: Findings from routine clinical practice. Clin Infect Dis. 2023 Aug 9:ciad460. (b)

Okoli GN, Rabbani R, Al-Juboori A, Copstein L, Askin N, Abou-Setta AM. Antiviral drugs for coronavirus disease 2019 (COVID-19): a systematic review with network meta-analysis. Expert Rev Anti Infect Ther. 2022; 20: 267-78.

Özlüşen B, Kozan Ş, Akcan RE, Kalender M, Yaprak D, Peltek İB, et al. Effectiveness of favipiravir in COVID-19: a live systematic review. Eur J Clin Microbiol Infect Dis. 2021; 40: 2575-83.

Pasin L, Cavalli G, Navalesi P, Sella N, Landoni G, Yavorovskiy AG, et al. Anakinra for patients with COVID-19: a meta-analysis of non-randomized cohort studies. Eur J Intern Med. 2021; 86: 34-40.

Pathak DSK, Salunke DAA, Thivari DP, Pandey A, Nandy DK, Harish V K Ratna D, et al. No benefit of hydroxychloroquine in COVID-19: Results of Systematic Review and Meta-Analysis of Randomized Controlled Trials". Diabetes Metab Syndr. 2020; 14: 1673-80.

Peng J, Fu M, Mei H, Zheng H, Liang G, She X, et al. Efficacy and secondary infection risk of tocilizumab, sarilumab and anakinra in COVID-19 patients: A systematic review and meta-analysis. Rev Med Virol. 2022; 32: e2295. (a)

Peng J, She X, Mei H, Zheng H, Fu M, Liang G, et al. Association between tocilizumab treatment and clinical outcomes of COVID-19 patients: a systematic review and meta-analysis. Aging (Albany NY). 2022; 14: 557-71. (b)

Petersen JJ, Jørgensen CK, Faltermeier P, Siddiqui F, Feinberg J, Nielsen EE, et al. Drug interventions for prevention of COVID-19 progression to severe disease in outpatients: a systematic review with meta-analyses and trial sequential analyses (The LIVING Project). BMJ Open. 2023; 13: e064498.

Pinzon RT, Wijaya VO, Buana RB. Interleukin-6 (IL-6) inhibitors as therapeutic agents for coronavirus disease 2019 (COVID-19): A systematic review and meta-analysis. J Infect Public Health. 2021; 14: 1001-9.

Putman M, Chock YPE, Tam H, Kim AHJ, Sattui SE, Berenbaum F, et al; COVID-19 Global Rheumatology Alliance. Antirheumatic Disease Therapies for the Treatment of COVID-19: A Systematic Review and Meta-Analysis. Arthritis Rheumatol. 2021; 73: 36-47.

RECOVERY Collaborative Group. Tocilizumab in patients admitted to hospital with COVID-19 (RECOVERY): a randomised, controlled, open-label, platform trial. Lancet. 2021; 397: 1637-45.

RECOVERY Collaborative Group. Casirivimab and imdevimab in patients admitted to hospital with COVID-19 (RECOVERY): a randomised, controlled, open-label, platform trial. Lancet. 2022; 399: 665-76.

Regkirona 60 mg/mL concentrate for solution for infusion. Ficha Técnica. Disponible en URL: https://www.ema.europa.eu/en/documents/product-information/ronapreve-epar-product-information_es.pdf (acceso: 17/11/2023).

Reis S, Metzendorf M-I, Kuehn R, Popp M, Gagyor I, Kranke P, et al. Nirmatrelvir combined with ritonavir for preventing and treating COVID-19. Cochrane Database of Systematic Reviews 2022, Issue 9. Art. No.: CD015395. DOI: 10.1002/14651858.CD015395.pub2.

Rezaei S, Fatemi B, Karimi Majd Z, Minaei H, Peikanpour M, Anjidani N, et al. Efficacy and safety of Tocilizumab in severe and critical COVID-19: A Systematic Review and Meta-Analysis. Expert Rev Clin Immunol. 2021; 17: 499-511.

Ronapreve 300 mg + 300 mg solución inyectable y para perfusión. Ficha técnica. Disponible en URL: https://ec.europa.eu/health/documents/community-register/2021/20211112154000/ anx_154000_es.pdf (acceso: 17/11/2023).

Rosas IO, Bräu N, Waters M, Go RC, Hunter BD, Bhagani S, et al. Tocilizumab in Hospitalized Patients with Severe Covid-19 Pneumonia. N Engl J Med. 2021; 384: 1503-16.

Sarma P, Kaur H, Kumar H, Mahendru D, Avti P, Bhattacharyya A, et al. Virological and clinical cure in COVID-19 patients treated with hydroxychloroquine: A systematic review and meta-analysis. J Med Virol. 2020; 92: 776-85.

Shang W, Zhang Y, Wang G, Han D. Anakinra was not associated with lower mortality in hospitalised COVID-19 patients: A systematic review and meta-analysis of randomized controlled trials. Rev Med Virol. 2023; 33: e2418.

Shrestha DB, Budhathoki P, Khadka S, Shah PB, Pokharel N, Rashmi P. Favipiravir versus other antiviral or standard of care for COVID-19 treatment: a rapid systematic review and meta-analysis. Virol J. 2020; 17: 141.

Shrestha DB, Budhathoki P, Syed NI, Rawal E, Raut S, Khadka S. Remdesivir: A potential game-changer or just a myth? A systematic review and meta-analysis. Life Sci. 2021; 264: 118663.

Siemieniuk RA, Bartoszko JJ, Díaz Martinez JP, Kum E, Qasim A, Zeraatkar D, et al. Antibody and cellular therapies for treatment of covid-19: a living systematic review and network meta-analysis. BMJ. 2021; 374: n2231.

Singh AK, Singh A, Singh R, Misra A. Hydroxychloroquine in patients with COVID-19: A Systematic Review and meta-analysis. Diabetes Metab Syndr. 2020; 14: 589-96.

Singh B, Ryan H, Kredo T, Chaplin M, Fletcher T. Chloroquine or hydroxychloroquine for prevention and treatment of COVID-19. Cochrane Database of Systematic Reviews 2021, Issue 2. Art. No.: CD013587. DOI: 10.1002/14651858.CD013587.pub2. (a)

Singh S, Khera D, Chugh A, Khera PS, Chugh VK. Efficacy and safety of remdesivir in COVID-19 caused by SARS-CoV-2: a systematic review and meta-analysis. BMJ Open. 2021; 11: e048416. (b)

Snow TAC, Saleem N, Ambler G, Nastouli E, Singer M, Arulkumaran N. Tocilizumab in COVID-19: a meta-analysis, trial sequential analysis, and meta-regression of randomized-controlled trials. Intensive Care Med. 2021; 47: 641-52.

Tharmarajah E, Buazon A, Patel V, Hannah JR, Adas M, Allen VB, et al. IL-6 inhibition in the treatment of COVID-19: A meta-analysis and meta-regression. J Infect. 2021; 82: 178-85.

Tian F, Chen Z, Feng Q. Nirmatrelvir-ritonavir compared with other antiviral drugs for the treatment of COVID-19 patients: A systematic review and meta-analysis. J Med Virol. 2023; 95: e28732.

Tleyjeh IM, Kashour Z, Riaz M, Hassett L, Veiga VC, Kashour T. Efficacy and safety of tocilizumab in COVID-19 patients: a living systematic review and meta-analysis, first update. Clin Microbiol Infect. 2021; 27: 1076-82.

Vela D, Vela-Gaxha Z, Rexhepi M, Olloni R, Hyseni V, Nallbani R. Efficacy and safety of tocilizumab versus standard care/placebo in patients with COVID-19; a systematic review and meta-analysis of randomized clinical trials. Br J Clin Pharmacol. 2022; 88: 1955-63.

Viswanatha GL, Anjana Male CKVLSN, Shylaja H. Efficacy and safety of tocilizumab in the management of COVID-19: a systematic review and meta-analysis of observational studies. Clin Exp Rheumatol. 2022; 40: 634-46.

Wang Y, Zheng J, Zhu K, Xu C, Wang D, Hou M. The effect of tixagevimab-cilgavimab on clinical outcomes in patients with COVID-19: A systematic review with meta-analysis. J Infect. 2023; 86: e15-17.

Wei Q, Lin H, Wei RG, Chen N, He F, Zou DH, et al. Tocilizumab treatment for COVID-19 patients: a systematic review and meta-analysis. Infect Dis Poverty. 2021; 10: 71.

WHO Rapid Evidence Appraisal for COVID-19 Therapies (REACT) Working Group; Shankar-Hari M, Vale CL, Godolphin PJ, Fisher D, Higgins JPT, et al. Association Between Administration of IL-6 Antagonists and Mortality Among Patients Hospitalized for COVID-19: A Meta-analysis. JAMA. 2021; 326: 499-518.

WHO Solidarity Trial Consortium. Remdesivir and three other drugs for hospitalised patients with COVID-19: final results of the WHO Solidarity randomised trial and updated meta-analyses. Lancet. 2022; 399: 1941-53.

Wu L, Zheng Y, Liu J, Luo R, Wu D, Xu P, et al. Comprehensive evaluation of the efficacy and safety of LPV/r drugs in the treatment of SARS and MERS to provide potential treatment options for COVID-19. Aging (Albany NY). 2021; 13: 10833-52.

Xevudy. International non-proprietary name: sotrovimab. CHMP assessment report. Procedure No. EMEA/H/C/005676/0000. Committee for Medicinal Products for Human Use (CHMP). European Medicines Agency. EMA/834036/2022. 16 December 2021. Disponible en URL: https://www.ema.europa.eu/en/documents/assessment-report/xevudy-epar-public-assessment-report_en.pdf (acceso: 17/11/2023).

Yang M, Li A, Jiang L, Wang Y, Tran C, Ao G. Regdanvimab improves disease mortality and morbidity in patients with COVID-19: A meta-analysis. J Infect. 2022; 85: e122-4.

Yu SY, Koh DH, Choi M, Ryoo S, Huh K, Yeom JS, et al. Clinical efficacy and safety of interleukin-6 receptor antagonists (tocilizumab and sarilumab) in patients with COVID-19: a systematic review and meta-analysis. Emerg Microbes Infect. 2022; 11: 1154-65.

**SUPPLEMENT 3. ARTICLES EXCLUDED FROM THE SYNTHESIS**

1. Bellos I. A metaresearch study revealed susceptibility of Covid-19 treatment research to white hat bias: first, do no harm. J Clin Epidemiol. 2021 Aug; 136:55-63.
2. Diaz-Arocutipa C, Brañez-Condorena A, Hernandez AV. QTc prolongation in COVID-19 patients treated with hydroxychloroquine, chloroquine, azithromycin, or lopinavir/ritonavir: A systematic review and meta-analysis. Pharmacoepidemiol Drug Saf. 2021 Jun;30(6):694-706.
3. Izcovich A, Siemieniuk RA, Bartoszko JJ, Ge L, Zeraatkar D, Kum E, Qasim A, Khamis AM, Rochwerg B, Agoritsas T, Chu DK, McLeod SL, Mustafa RA, Vandvik P, Brignardello-Petersen R. Adverse effects of remdesivir, hydroxychloroquine and lopinavir/ritonavir when used for COVID-19: systematic review and meta-analysis of randomised trials. BMJ Open. 2022 Mar 2;12(3):e048502.
4. Liao ZM, Zhang ZM, Liu Q. Hydroxychloroquine/chloroquine and the risk of acute kidney injury in COVID-19 patients: a systematic review and meta-analysis. Ren Fail. 2022 Dec;44(1):415-425.
5. Kheirabadi D, Haddad F, Mousavi-Roknabadi RS, Rezaeisadrabadi M, Dehghan H, Fazlzadeh A. A complementary critical appraisal on systematic reviews regarding the most efficient therapeutic strategies for the current COVID-19 (SARS-CoV-2) pandemic. J Med Virol. 2021 May;93(5):2705-2721.
6. Paludan-Müller AS, Lundh A, Page MJ, Munkholm K. Protocol: Benefits and harms of remdesivir for COVID-19 in adults: A systematic review with meta-analysis. PLoS One. 2021 Nov 29;16(11):e0260544.
7. Zhao J, Cui W, Tian BP. Efficacy of tocilizumab treatment in severely ill COVID-19 patients. Crit Care. 2020 Aug 27;24(1):524.
8. Million M, Roussel Y, Gautret P, Raoult D. Effect of hydroxychloroquine and azithromycin on SARS-CoV-2 clearance in COVID-19 patients, a meta-analysis. Int J Antimicrob Agents. 2021 Jan;57(1):106240.
9. Yokoyama Y, Briasoulis A, Takagi H, Kuno T. Effect of remdesivir on patients with COVID-19: A network meta-analysis of randomized control trials. Virus Res. 2020; 288: 198137.
10. Kow CS, Aldeyab M, Hasan SS. Effect of remdesivir on mortality in patients with COVID-19: A meta-analysis of randomized control trials. J Med Virol. 2021 Apr;93(4):1860-1861.
11. Manabe T, Kambayashi D, Akatsu H, Kudo K. Favipiravir for the treatment of patients with COVID-19: a systematic review and meta-analysis. BMC Infect Dis. 2021; 21: 489.
12. Albuquerque AM, Tramujas L, Sewanan LR, Williams DR, Brophy JM. Mortality Rates Among Hospitalized Patients With COVID-19 Infection Treated With Tocilizumab and Corticosteroids: A Bayesian Reanalysis of a Previous Meta-analysis. JAMA Netw Open. 2022 Feb 1;5(2):e220548.

13. Zheng Q, Ma P, Wang M, Cheng Y, Zhou M, Ye L, Feng Z, Zhang C. Efficacy and safety of Paxlovid for COVID-19: a meta-analysis. J Infect. 2023 Jan;86(1):66-117.
14. Liu W, Zhou P, Chen K, Ye Z, Liu F, Li X, He N, Wu Z, Zhang Q, Gong X, Tang Q, Du X, Ying Y, Xu X, Zhang Y, Liu J, Li Y, Shen N, Couban RJ, Ibrahim QI, Guyatt G, Zhai S. Efficacy and safety of antiviral treatment for COVID-19 from evidence in studies of SARS-CoV-2 and other acute viral infections: a systematic review and meta-analysis. CMAJ. 2020 Jul;192(27):E734-E744.
15. Lai CC, Wang YH, Chen KH, Chen CH, Wang CY. The Clinical Efficacy and Safety of Anti-Viral Agents for Non-Hospitalized Patients with COVID-19: A Systematic Review and Network Meta-Analysis of Randomized Controlled Trials. Viruses. 2022; 14: 1706.
16. Selvarajan S, Anandaradje A, Shivabasappa S, Melepurakkal Sadanandan D, Nair NS, George M. Efficacy of pharmacological interventions in COVID-19: A network meta-analysis. Br J Clin Pharmacol. 2022 Sep;88(9):4080-4091.
17. Pitre T, Van Alstine R, Chick G, Leung G, Mikhail D, Cusano E, Khalid F, Zeraatkar D. Antiviral drug treatment for nonsevere COVID-19: a systematic review and network meta-analysis. CMAJ. 2022 Jul 25;194(28):E969-E980.
18. Cheng Q, Chen J, Jia Q, Fang Z, Zhao G. Efficacy and safety of current medications for treating severe and non-severe COVID-19 patients: an updated network meta-analysis of randomized placebo-controlled trials. Aging (Albany NY). 2021 Sep 16;13(18):21866-21902.
19. Thoguluva Chandrasekar V, Venkatesalu B, Patel HK, Spadaccini M, Manteuffel J, Ramesh M. Systematic review and meta-analysis of effectiveness of treatment options against SARS-CoV-2 infection. J Med Virol. 2021 Feb;93(2):775-785.
20. Juul S, Nielsen EE, Feinberg J, Siddiqui F, Jørgensen CK, Barot E, Nielsen N, Bentzer P, Veroniki AA, Thabane L, Bu F, Klingenberg S, Gluud C, Jakobsen JC. Interventions for treatment of COVID-19: A living systematic review with meta-analyses and trial sequential analyses (The LIVING Project). PLoS Med. 2020 Sep 17;17(9):e1003293.
21. Misra S, Nath M, Hadda V, Vibha D. Efficacy of various treatment modalities for nCOV-2019: A systematic review and meta-analysis. Eur J Clin Invest. 2020 Nov;50(11):e13383.
22. Zhong H, Wang Y, Zhang ZL, Liu YX, Le KJ, Cui M, Yu YT, Gu ZC, Gao Y, Lin HW. Efficacy and safety of current therapeutic options for COVID-19 - lessons to be learnt from SARS and MERS epidemic: A systematic review and meta-analysis. Pharmacol Res. 2020 Jul;157:104872.
23. Abeldaño Zuñiga RA, Coca SM, Abeldaño GF, González-Villoria RAM. Clinical effectiveness of drugs in hospitalized patients with COVID-19: a systematic review and meta-analysis. Ther Adv Respir Dis. 2021 Jan-Dec;15:17534666211007214.
24. Crichton ML, Goeminne PC, Tuand K, Vandendriessche T, Tonia T, Roche N, Chalmers JD; European Respiratory Society COVID-19 Task Force. The impact of therapeutics on mortality in hospitalised patients with COVID-19: systematic review and meta-analyses informing the European Respiratory Society living guideline. Eur Respir Rev. 2021 Dec 15;30(162):210171.

25. Zhang C, Jin H, Wen YF, Yin G. Efficacy of COVID-19 Treatments: A Bayesian Network Meta-Analysis of Randomized Controlled Trials. Front Public Health. 2021 Sep 28;9:729559.
26. Wen W, Chen C, Tang J, Wang C, Zhou M, Cheng Y, Zhou X, Wu Q, Zhang X, Feng Z, Wang M, Mao Q. Efficacy and safety of three new oral antiviral treatment (molnupiravir, fluvoxamine and Paxlovid) for COVID-19：a meta-analysis. Ann Med. 2022 Dec;54(1):516-523.
27. Gastine S, Pang J, Boshier FAT, Carter SJ, Lonsdale DO, Cortina-Borja M, Hung IFN, Breuer J, Kloprogge F, Standing JF. Systematic Review and Patient-Level Meta-Analysis of SARS-CoV-2 Viral Dynamics to Model Response to Antiviral Therapies. Clin Pharmacol Ther. 2021 Aug;110(2):321-333.
28. Wang Y, Zheng J, Zhu K, Xu C, Wang D, Hou M. The effect of tixagevimab-cilgavimab on clinical outcomes in patients with COVID-19: A systematic review with meta-analysis. J Infect. 2023 Jan;86(1):e15-e17.
29. Ao G, Li A, Wang Y, Tran C, Qi X. Lack of efficacy for sotrovimab use in patients with COVID-19: A meta-analysis. J Infect. 2022 Jul;85(1):e10-e12.
30. Tao K, Tzou PL, Kosakovsky Pond SL, Ioannidis JPA, Shafer RW. Susceptibility of SARS-CoV-2 Omicron Variants to Therapeutic Monoclonal Antibodies: Systematic Review and Meta-analysis. Microbiol Spectr. 2022 Aug 31;10(4):e0092622.
31. Alegre-Del-Rey EJ, Fénix-Caballero S, Salmerón-Navas FJ, Gil-Sierra MD, Sierra-Sánchez JF, Díaz-Alersi Rosety RL. Systematic review and meta-analysis of interleulin-6 inhibitors in reducing mortality for hospitalized patients with COVID-19. Farm Hosp. 2022 May 4;46(3):166-172.
32. Malık H, Bınt Abdul Jabbar H, Latıf F, Sarfraz A, Sarfraz Z, Sarfraz M. The efficacy of anakinra compared to standard care of treatment for COVID-19: a meta-analysis. Turk J Med Sci. 2022 Jun;52(3):547-553.
33. Naveed Z, Sarwar M, Ali Z, Saeed D, Choudhry K, Sarfraz A, Sarfraz Z, Felix M, Cherrez-Ojeda I. Anakinra treatment efficacy in reduction of inflammatory biomarkers in COVID-19 patients: A meta-analysis. J Clin Lab Anal. 2022 Jun;36(6):e24434.
34. Dahms K, Mikolajewska A, Ansems K, Metzendorf MI, Benstoem C, Stegemann M. Anakinra for the treatment of COVID-19 patients: a systematic review and meta-analysis. Eur J Med Res. 2023 Feb 25;28(1):100.
35. Prasad M, Elavarasi A, Madan K, Nischal N, Soneja M, Seth T, Sahoo RK, Sharma A, Garg P, Shalimar. Efficacy and safety of antivirals for Covid-19: A systematic review and meta-analysis. Natl Med J India. 2020 Jul-Aug;33(4):222-231.
36. Prakash A, Singh H, Kaur H, Semwal A, Sarma P, Bhattacharyya A, Dhibar DP, Medhi B. Systematic review and meta-analysis of effectiveness and safety of favipiravir in the management of novel coronavirus (COVID-19) patients. Indian J Pharmacol. 2020 Sep-Oct;52(5):414-421.
37. Lan SH, Lai CC, Chang SP, Lu LC, Hung SH, Lin WT. Favipiravir-based treatment for outcomes of patients with COVID-19: a systematic review and meta-analysis of randomized controlled trials. Expert Rev Clin Pharmacol. 2022 Jun;15(6):759-766.
38. Yang K, Zeng J, Dai W, Chen M, Yang F. A systematic review and Bayesian network meta-analysis for comparative safety assessment of favipiravir interventions in hospitalized COVID-19 patients. J Infect Dev Ctries. 2022 Sep 30;16(9):1406-1412.

39. Di Stefano L, Ogburn EL, Ram M, Scharfstein DO, Li T, Khanal P, Baksh SN, McBee N, Gruber J, Gildea MR, Clark MR, Goldenberg NA, Bennani Y, Brown SM, Buckel WR, Clement ME, Mulligan MJ, O'Halloran JA, Rauseo AM, Self WH, Semler MW, Seto T, Stout JE, Ulrich RJ, Victory J, Bierer BE, Hanley DF, Freilich D; Pandemic Response COVID-19 Research Collaboration Platform for HCQ/CQ Pooled Analyses. Hydroxychloroquine/chloroquine for the treatment of hospitalized patients with COVID-19: An individual participant data meta-analysis. PLoS One. 2022 Sep 29;17(9):e0273526.
40. Das S, Bhowmick S, Tiwari S, Sen S. An Updated Systematic Review of the Therapeutic Role of Hydroxychloroquine in Coronavirus Disease-19 (COVID-19). Clin Drug Investig. 2020 Jul;40(7):591-601.
41. Das RR, Behera B, Mishra B, Naik SS. Effect of chloroquine and hydroxychloroquine on COVID-19 virological outcomes: An updated meta-analysis. Indian J Med Microbiol. 2020 Jul-Dec;38(3 & 4):265-272.
42. Ayele Mega T, Feyissa TM, Dessalegn Bosho D, Kumela Goro K, Zeleke Negera G. The Outcome of Hydroxychloroquine in Patients Treated for COVID-19: Systematic Review and Meta-Analysis. Can Respir J. 2020 Oct 13;2020:4312519.
43. Shamshirian A, Hessami A, Heydari K, Navaei RA, Ebrahimzadeh MA, Yip GW, Ghasemian R, Sedaghat M, Baradaran H, Yazdi SM, Aboufazeli E, Jafarpour H, Dadgostar E, Tirandazi B, Sadeghnezhad R, Karimifar K, Eftekhari A, Shamshirian D. The Role of Hydroxychloroquine in COVID-19: A Systematic Review and Meta-Analysis. Ann Acad Med Singap. 2020 Oct;49(10):789-800.
44. Zang Y, Han X, He M, Shi J, Li Y. Hydroxychloroquine use and progression or prognosis of COVID-19: a systematic review and meta-analysis. Naunyn Schmiedebergs Arch Pharmacol. 2021 Apr;394(4):775-782.
45. Bignardi PR, Vengrus CS, Aquino BM, Cerci Neto A. Use of hydroxychloroquine and chloroquine in patients with COVID-19: a meta-analysis of randomized clinical trials. Pathog Glob Health. 2021 May;115(3):139-150.
46. Budhathoki P, Shrestha DB, Khadka S, Rawal E. Is Hydroxychloroquine with Azithromycin a Good Combination in COVID-19 Compared to Hydroxychloroquine Alone from Cardiac Perspective? A Systematic Review and Meta-Analysis. J Nepal Health Res Counc. 2021 Apr 23;19(1):1-9.
47. Chen PH, Jhou HJ, Ou-Yang LJ, Lee CH. Does hydroxychloroquine reduce mortality in patients with COVID-19? A meta-analysis with trial sequential analysis. Int J Clin Pract. 2021 Oct;75(10):e14448.
48. Choudhuri AH, Duggal S, Ahuja B, Biswas PS. The efficacy and safety of hydroxychloroquine (HCQ) in treatment of COVID19 -a systematic review and meta-analysis. Indian J Med Microbiol. 2021 Apr;39(2):159-170.
49. Di Castelnuovo A, Costanzo S, Cassone A, Cauda R, De Gaetano G, Iacoviello L. Hydroxychloroquine and mortality in COVID-19 patients: a systematic review and a meta-analysis of observational studies and randomized controlled trials. Pathog Glob Health. 2021 Oct-Dec;115(7-8):456-466.
50. Kumar J, Jain S, Meena J, Yadav A. Efficacy and safety of hydroxychloroquine/chloroquine against SARS-CoV-2 infection: A systematic review and meta-analysis. J Infect Chemother. 2021 Jun;27(6):882-889.

51. Tanni SE, Bacha HA, Naime A, Bernardo WM. Use of hydroxychloroquine to prevent SARS-CoV-2 infection and treat mild COVID-19: a systematic review and meta-analysis. J Bras Pneumol. 2021 Oct 15;47(5):e20210236.
52. Moraes VY, Marra AR, Matos LL, Serpa Neto A, Rizzo LV, Cendoroglo Neto M, Lenza M. Hydroxychloroquine for treatment of COVID-19 patients: a systematic review and meta-analysis of randomized controlled trials. Einstein (Sao Paulo). 2022 Dec 2;20:eRW0045.
53. Konwar M, Maurya M, Thatte UM, Gogtay NJ, Bose D. A Systematic Review and Meta-Analysis of the Safety of Hydroxychloroquine in a Randomized Controlled Trial and Observational Studies. Curr Rev Clin Exp Pharmacol. 2022;17(3):216-235.
54. Kumar S, Saurabh MK, Narasimha VL, Maharshi V. Efficacy of Interferon-β in Moderate-to-Severe Hospitalised Cases of COVID-19: A Systematic Review and Meta-analysis. Clin Drug Investig. 2021 Dec;41(12):1037-1046.
55. Nakhlband A, Fakhari A, Azizi H. Interferon-beta offers promising avenues to COVID-19 treatment: a systematic review and meta-analysis of clinical trial studies. Naunyn Schmiedebergs Arch Pharmacol. 2021 May;394(5):829-838.
56. Saleki K, Yaribash S, Banazadeh M, Hajihosseinlou E, Gouravani M, Saghazadeh A, Rezaei N. Interferon therapy in patients with SARS, MERS, and COVID-19: A systematic review and meta-analysis of clinical studies. Eur J Pharmacol. 2021 Sep 5;906:174248.
57. Xue C, Liu Z, Xiang H, Ye S, Ye Q. Efficacy of Interferon-Based Therapy for COVID-19: A Systematic Review and Meta-Analysis. Altern Ther Health Med. 2022 Oct;28(7):52-57.
58. Bhattacharyya A, Kumar S, Sarma P, Kaur H, Prajapat M, Shekhar N, Bansal S, Avti P, Hazarika M, Sharma S, Mahendru D, Prakash A, Medhi B. Safety and efficacy of lopinavir/ritonavir combination in COVID-19: A systematic review, meta-analysis, and meta-regression analysis. Indian J Pharmacol. 2020 Jul-Aug;52(4):313-323.
59. Verdugo-Paiva F, Izcovich A, Ragusa M, Rada G. Lopinavir-ritonavir for COVID-19: A living systematic review. Medwave. 2020 Jul 15;20(6):e7967.
60. Amani B, Khanijahani A, Amani B, Hashemi P. Lopinavir/Ritonavir for COVID-19: a Systematic Review and Meta-Analysis. J Pharm Pharm Sci. 2021;24:246-257.ç
61. Al-Abdouh A, Bizanti A, Barbarawi M, Jabri A, Kumar A, Fashanu OE, Khan SU, Zhao D, Antar AAR, Michos ED. Remdesivir for the treatment of COVID-19: A systematic review and meta-analysis of randomized controlled trials. Contemp Clin Trials. 2021 Feb;101:106272.
62. Piscoya A, Ng-Sueng LF, Parra Del Riego A, Cerna-Viacava R, Pasupuleti V, Roman YM, Thota P, White CM, Hernandez AV. Efficacy and harms of remdesivir for the treatment of COVID-19: A systematic review and meta-analysis. PLoS One. 2020 Dec 10;15(12):e0243705.
63. Verdugo-Paiva F, Acuña MP, Solá I, Rada G; COVID-19 L·OVE Working Group. Remdesivir for the treatment of COVID-19: a living systematic review. Medwave. 2020 Dec 9;20(11):e8080.
64. Enoki Y, Igarashi Y, Watabe Y, Honma K, Suzuki Y, Hayashi Y, Hiraoka K, Taguchi K, Matsumoto K. Remdesivir for the treatment of coronavirus COVID-19: A meta-analysis of randomised controlled trials. J Glob Antimicrob Resist. 2021 Mar;24:81-82.

65. Tasavon Gholamhoseini M, Yazdi-Feyzabadi V, Goudarzi R, Mehrolhassani MH. Safety and Efficacy of Remdesivir for the Treatment of COVID-19: A Systematic Review and Meta-Analysis. J Pharm Pharm Sci. 2021;24:237-245.
66. Okoli GN, Rabbani R, Copstein L, Al-Juboori A, Askin N, Abou-Setta AM. Remdesivir for coronavirus disease 2019 (COVID-19): a systematic review with meta-analysis and trial sequential analysis of randomized controlled trials. Infect Dis (Lond). 2021 Sep;53(9):691-699.
67. Rezagholizadeh A, Khiali S, Sarbakhsh P, Entezari-Maleki T. Remdesivir for treatment of COVID-19; an updated systematic review and meta-analysis. Eur J Pharmacol. 2021 Apr 15;897:173926.
68. Konwar M, Maurya M, Bose D. A Meta-Analysis of Safety of Different Regimens of Remdesivir in COVID-19 Patients. Curr Drug Saf. 2022;17(2):158-167.
69. Kotecha P, Light A, Checcucci E, Amparore D, Fiori C, Porpiglia F, Dasgupta P, Elhage O. Repurposing of drugs for COVID-19: a systematic review and meta-analysis. Panminerva Med. 2022 Mar;64(1):96-114.
70. Tanni SE, Silvinato A, Floriano I, Bacha HA, Barbosa AN, Bernardo WM. Use of remdesivir in patients with COVID-19: a systematic review and meta-analysis. J Bras Pneumol. 2022 Feb 2;48(1):e20210393.
71. Viveiros Rosa SG, Santos WC. Remdesivir: an overview of patenting trends, clinical evidence on COVID-19 treatment, pharmacology and chemistry. Pharm Pat Anal. 2022 Mar;11(2):57-73.
72. Godolphin PJ, Fisher DJ, Berry LR, Derde LPG, Diaz JV, Gordon AC, Lorenzi E, Marshall JC, Murthy S, Shankar-Hari M, Sterne JAC, Tierney JF, Vale CL. Association between tocilizumab, sarilumab and all-cause mortality at 28 days in hospitalised patients with COVID-19: A network meta-analysis. PLoS One. 2022 Jul 8;17(7):e0270668.
73. Avni T, Leibovici L, Cohen I, Atamna A, Guz D, Paul M, Gafter-Gvili A, Yahav D. Tocilizumab in the treatment of COVID-19-a meta-analysis. QJM. 2021 Nov 5;114(8):577-586.
74. Hariyanto TI, Hardyson W, Kurniawan A. Efficacy and Safety of Tocilizumab for Coronavirus Disease 2019 (Covid-19) Patients: A Systematic Review and Meta-analysis. Drug Res (Stuttg). 2021 May;71(5):265-274.
75. Kow CS, Hasan SS. The effect of tocilizumab on mortality in hospitalized patients with COVID-19: a meta-analysis of randomized controlled trials. Eur J Clin Pharmacol. 2021 Aug;77(8):1089-1094.
76. Mahroum N, Watad A, Bridgewood C, Mansour M, Nasr A, Hussein A, Khamisy-Farah R, Farah R, Gendelman O, Lidar M, Shoenfeld Y, Amital H, Kong JD, Wu J, Bragazzi NL, McGonagle D. Systematic Review and Meta-Analysis of Tocilizumab Therapy versus Standard of Care in over 15,000 COVID-19 Pneumonia Patients during the First Eight Months of the Pandemic. Int J Environ Res Public Health. 2021 Aug 30;18(17):9149.
77. Malgie J, Schoones JW, Pijls BG. Decreased Mortality in Coronavirus Disease 2019 Patients Treated With Tocilizumab: A Rapid Systematic Review and Meta-analysis of Observational Studies. Clin Infect Dis. 2021 Jun 1;72(11):e742-e749.

78. Nugroho CW, Suryantoro SD, Yuliasih Y, Rosyid AN, Asmarawati TP, Andrianto L, Setiawan HW, Mahdi BA, Windradi C, Agustin ED, Fajar JK. Optimal use of tocilizumab for severe and critical COVID-19: a systematic review and meta-analysis. F1000Res. 2021 Feb 4;10:73.
79. Sarfraz A, Sarfraz Z, Sarfraz M, Aftab H, Pervaiz Z. Tocilizumab and COVID-19: a meta-analysis of 2120 patients with severe disease and implications for clinical trial methodologies. Turk J Med Sci. 2021 Jun 28;51(3):890-897.
80. Selvaraj V, Khan MS, Bavishi C, Dapaah-Afriyie K, Finn A, Lal A, Mylonakis E. Tocilizumab in Hospitalized Patients with COVID-19: A Meta Analysis of Randomized Controlled Trials. Lung. 2021 Jun;199(3):239-248.
81. Boppana TK, Mittal S, Madan K, Mohan A, Hadda V, Guleria R. Tocilizumab for COVID-19: A systematic review and meta-analysis of randomized controlled trials. Monaldi Arch Chest Dis. 2022 Feb 4;92(4). doi: 10.4081/monaldi.2022.2136. PMID: 35130679.
82. Gupta S, Padappayil RP, Bansal A, Daouk S, Brown B. Tocilizumab in patients hospitalized with COVID-19 pneumonia: systematic review and meta-analysis of randomized controlled trials. J Investig Med. 2022 Jan;70(1):55-60.
83. Lim PC, Wong KL, Rajah R, Chong MF, Chow TS, Subramaniam S, Lee CY. Comparing the efficacy of tocilizumab with corticosteroid therapy in treating COVID-19 patients: a systematic review and meta-analysis. Daru. 2022 Jun;30(1):211-228.
84. Mutua V, Henry BM, Csefalvay CV, Cheruiyot I, Vikse J, Lippi G, Bundi B, Mong'are N. Tocilizumab in addition to standard of care in the management of COVID-19: a meta-analysis of RCTs. Acta Biomed. 2022 Mar 14;93(1):e2022014.
85. Piscoya A, Parra Del Riego A, Cerna-Viacava R, Rocco J, Roman YM, Escobedo AA, Pasupuleti V, White CM, Hernandez AV. Efficacy and harms of tocilizumab for the treatment of COVID-19 patients: A systematic review and meta-analysis. PLoS One. 2022 Jun 3;17(6):e0269368.
86. Zhang J, Chen C, Yang Y, Yang J. Effectiveness of tocilizumab in the treatment of hospitalized adults COVID-19: A systematic review and meta-analysis. Medicine (Baltimore). 2022 Mar 4;101(9):e28967.
87. Talaie H, Hosseini SM, Nazari M, Fakhri Y, Mousavizadeh A, Vatanpour H, Firoozfar A. Is there any potential management against COVID-19? A systematic review and meta-analysis. Daru. 2020 Dec;28(2):765-777.
88. Wang M, Wu T, Zuo Z, You Y, Yang X, Pan L, Hu Y, Luo X, Jiang L, Xia Z, Deng M. Evaluation of current medical approaches for COVID-19: a systematic review and meta-analysis. BMJ Support Palliat Care. 2021 Mar;11(1):45-52.
89. Juul S, Nielsen EE, Feinberg J, Siddiqui F, Jørgensen CK, Barot E, Holgersson J, Nielsen N, Bentzer P, Veroniki AA, Thabane L, Bu F, Klingenberg S, Gluud C, Jakobsen JC. Interventions for treatment of COVID-19: Second edition of a living systematic review with meta-analyses and trial sequential analyses (The LIVING Project). PLoS One. 2021 Mar 11;16(3):e0248132.
90. Qiu R, Li J, Xiao Y, Gao Z, Weng Y, Zhang Q, Wang C, Gong H, Li W. The therapeutic effect and safety of the drugs for COVID-19: A systematic review and meta-analysis. Medicine (Baltimore). 2021 Apr 23;100(16):e25532.

91. Wu SS, Zhou QX, Zeng XY, Zhang JX, Yang ZR, Yang QQ, Zhang ZL, Chen YH, Sun F, Zhan SY. Comparative effectiveness and safety of 32 pharmacological interventions recommended by guidelines for coronavirus disease 2019: a systematic review and network meta-analysis combining 66 trials. Chin Med J (Engl). 2021 Jul 27;134(16):1920-1929.
92. Cheng Q, Zhao G, Chen J, Jia Q, Fang Z. Comparative efficacy and safety of pharmacological interventions for severe COVID-19 patients: An updated network meta-analysis of 48 randomized controlled trials. Medicine (Baltimore). 2022 Oct 14;101(41):e30998.
93. Wang X, Xie P, Sun G, Zhao M, Deng Z, Zhou Y, Bao S. A systematic review and meta-analysis of the efficacy and safety of arbidol in the treatment of coronavirus disease 2019. Medicine (Baltimore). 2020 Jul 24;99(30):e21402.
94. Amani B, Amani B, Zareei S, Zareei M. Efficacy and safety of arbidol (umifenovir) in patients with COVID-19: A systematic review and meta-analysis. Immun Inflamm Dis. 2021 Dec;9(4):1197-1208.
95. Coomes EA, Haghbayan H. Interleukin-6 in Covid-19: A systematic review and meta-analysis. Rev Med Virol. 2020 Nov;30(6):1-9.
96. Mojtabavi H, Saghazadeh A, Rezaei N. Interleukin-6 and severe COVID-19: a systematic review and meta-analysis. Eur Cytokine Netw. 2020 Jun 1;31(2):44-49.

## SUPPLEMENT 4. ARTICLES ON DRUGS DISCARDED EARLY (Alphabetical order)

Annane D, Heming N, Grimaldi-Bensouda L, Frémeaux-Bacchi V, Vigan M, Roux AL, et al; COVID 19 Collaborative Group. Eculizumab as an emergency treatment for adult patients with severe COVID-19 in the intensive care unit: A proof-of-concept study. EClinicalMedicine. 2020; 28: 100590.

Burwick RM, Dellapiana G, Newman RA, Smithson SD, Naqvi M, Williams J 3rd, et al. Complement blockade with eculizumab for treatment of severe Coronavirus Disease 2019 in pregnancy: A case series. Am J Reprod Immunol. 2022; 88: e13559.

Chen H, Zhang Z, Wang L, Huang Z, Gong F, Li X, et al. First clinical study using HCV protease inhibitor danoprevir to treat COVID-19 patients. Medicine (Baltimore). 2020; 99: e23357.

Diurno F, Numis FG, Porta G, Cirillo F, Maddaluno S, Ragozzino A, et al. Eculizumab treatment in patients with COVID-19: preliminary results from real life ASL Napoli 2 Nord experience. Eur Rev Med Pharmacol Sci. 2020; 24: 4040-7.

EUnetHTA. “Rolling Collaborative Review” of Covid-19 treatments. APN01 for the treatment of COVID-19- Project ID: RCR09. Monitoring Report. Version 6.0, May 2021. Disponible en URL: https://www.eunethta.eu/wp-content/uploads/2021/05/EUnetHTA-Covid-19_RCR09_APN01__V6.0.pdf (acceso: 25/10/2023).

Miao M, Jing X, De Clercq E, Li G. Danoprevir for the Treatment of Hepatitis C Virus Infection: Design, Development, and Place in Therapy. Drug Des Devel Ther. 2020; 14: 2759-74.

NCT04335136. Recombinant Human Angiotensin-converting Enzyme 2 (rhACE2) as a Treatment for Patients With COVID-19 (APN01-COVID-19). Disponible en URL: https://clinicaltrials.gov/study/NCT04335136?intr=APN01&cond=Covid19&rank=1&tab=results (acceso: 25/10/2023).

NCT04343651. Study to Evaluate the Efficacy and Safety of Leronlimab for Mild to Moderate COVID-19. Disponible en URL: https://clinicaltrials.gov/study/NCT04343651?cond =Covid19&intr=Leronlimab&rank=1 (acceso: 25/10/2023).

Ruggenenti P, Di Marco F, Cortinovis M, Lorini L, Sala S, Novelli L, et al. Eculizumab in patients with severe coronavirus disease 2019 (COVID-19) requiring continuous positive airway pressure ventilator support: Retrospective cohort study. PLoS One. 2021; 16: e0261113.

Yang B, Fulcher JA, Ahn J, Berro M, Goodman-Meza D, Dhody K, et al. Clinical Characteristics and Outcomes of Coronavirus Disease 2019 Patients Who Received Compassionate-Use Leronlimab. Clin Infect Dis. 2021; 73: e4082-9.

## SUPPLEMENT 5. SUPPLEMENTARY TABLES

**Table S1.** Interleukin 1 (IL-1) inhibitors: Anakinra. Meta-analysis of its effectiveness in the treatment of COVID-19.

| Year | First author | Meta-analysis type | PRISMA / Cochrane / WHO § | Quality NHLBI # | Clinical trials design | Comparator | Main efficacy endpoints | No studies included | No patients included | OR/RR/HR (95% CI; p) | Hetero-geneity ($I^2$) * | Magnitude of effect: Cohen's "d" ** |
|---|---|---|---|---|---|---|---|---|---|---|---|---|
| 2020 | Kim | NMA | PRISMA | High | Observational | Standard treatment | Mortality<br>Severe disease progression | 1 | NA | 0.30 (0.11-0.82;0.019)<br>0.22 (0.09-0.56;0.002) | NA<br>NA | M: 0.664<br>L: 0.835 |
| 2021 | Barkas | REM/ FEM | PRISMA | High | RCT | Standard treatment | Mortality<br>Admission to ICU with MV | 9 | 485 | 0.32 (0.23-0.45;<0.00001)<br>0.38 (0.17-0.85;0.02) | LOW: 0%<br>MED: 67% | M: 0.628<br>M: 0.533 |
| 2021 | Khan | REM | PRISMA | High | Cohorts¶<br>Retrospectives | Standard treatment | Mortality | 4<br>3 | 196<br>29 | 0.24 (0.07-0.79; ND)<br>0.70 (0.31-1.58;0.226) | NA<br>LOW: 32.8% | M: 0.787<br>No association |
| 2021 | Pasin | REM/ FEM | PRISMA | High | Cohorts¶ | Standard treatment | Mortality<br>Admission to ICU with MV | 4 | 111 | 0.26 (0.14-0.48;0.0001)<br>0.45 (0.25-0.82;0.008) | LOW: 0%<br>LOW: 19% | M: 0.743<br>S: 0.440 |
| 2022 | Peng | REM | PRISMA | High | Case-control | Standard treatment | Mortality<br>2nd infection risk | 3 | 85 | 0.41 (0.19-0.85;0.017)<br>1.44 (0.47-4.43;0.520) | MED: 27.6%<br>NA | S: 0.492<br>No association |
| 2021 | Putman | REM | PRISMA | High | Cohorts¶ | Standard treatment | Mortality | 2 | 60 | 0.25 (0.12-0.52;0.0002) | LOW: 0% | M: 0.764 |
| 2023 | Shang | REM/ FEM | PRISMA | High | RCT | Standard treatment/PBO | Mortality (90 days) | 5 | 941 | 1.01 (0.73-1.39;0.97) | LOW: 55% | No association |

*The following criteria were used: (i) up to 25%, low heterogeneity (LOW); (ii) between > 25% and < 75%, moderate heterogeneity (MED); and, finally, (iii) if ≥ 75%, high heterogeneity (HIGH) (*Higgins, 2003*); ** By convention, for Cohen's "d" of 0-0.4; 0.5-0.7 and ≥ 0.8 are considered small (S), medium (M) and large (L) effect sizes respectively (*Chen, 2010*). Cohen's d was calculated using the tool available at the following URL: https://www.escal.site/. § References: PRISMA: *Liberati, 2009; Moher, 2009; Page, 2021; Cochrane: Cochrane, 2022; WHO: Shankar-Hari, 2021*. # Reference: *National, 2023*; ¶ Non randomized prospective cohort studies. Abbreviations: 95% CI: 95% confidence interval; FEM: fixed effects model; HIGH: high heterogeneity; HR: hazard ratio; L: large effect; LOW: low heterogeneity; M: medium effect; MED: average heterogeneity; MV: mechanical ventilation; NHLBI: National Heart, Lung and Blood Institute (*National, 2023*); NA: not available; NMA: network meta-analysis; OR: odds ratio; p: statistical significance; PBO: placebo; RCT: randomized clinical trials; REM: random effects model; RR: relative risk; S: small effect.

**Table S2.** Interleukin 6 (IL-6) inhibitors: Tocilizumab. Meta-analysis of its effectiveness in the treatment of COVID-19.

| Year | First author | Meta-analysis type | PRISMA / Cochrane / WHO § | Quality NHLBI # | Clinical trials design | Comparator | Main efficacy endpoints | No studies included | No patients included | OR/RR/HR (95% CI; p) | Hetero-geneity ($I^2$) * | Magnitude of effect: Cohen's "d" ** |
|---|---|---|---|---|---|---|---|---|---|---|---|---|
| 2020 | Aziz | REM | PRISMA | High | RCT<br>Cohorts | Standard treatment | Mortality<br><br>MV need | 23 | 1897 | Risk difference:<br>-0.06 (-0.12; --0.01;0.03)<br>-0.11 (-0.19; -0.02;0.01) | <br>HIGH: 80.9%<br>MED: 74.0% | - |
| 2020 | Berardicurti | REM | PRISMA | High | Prospectives<br>Retrospectives | Standard treatment | Mortality | 22 | 1520 | 0.47 (0.22-0.98;0.04) | MED: 68% | S: 0.416 |
| 2020 | Lan | REM/<br>FEM | PRISMA | High | Retrospectives | Standard treatment /PBO | Mortality<br>ICU admission risk | 7 | 240 | 0.62 (0.31-1.22; NA) | MED: 68% | No association |
| 2021 | Alkofide | REM | PRISMA | High | RCT<br>Retrospectives | Standard treatment | Mortality | 17 | 727 | 0.58 (0.42-0.81; NA) | MED: 71% | S: 0.300 |
| 2021 | Chen | REM/<br>FEM | Cochrane | High | RCT<br>Cohorts | NA | Mortality<br>ICU admission<br>MV<br>Secondary infection | 30<br>7<br>13<br>10 | 2651<br>663<br>705<br>1201 | 0.74 (0.59-0.93; NA)<br>1.40 (0.64-3.06; NA)<br>0.83 (0.57-1.22; NA)<br>1.30 (0.97-1.74; NA) | HIGH: 80%<br>HIGH: 88%<br>MED: 65%<br>MED: 65% | S: 0.166<br>No association<br>No association<br>No association |
| 2021 | Ghosn | REM | Cochrane | High | RCT | Standard treatment /PBO | Mortality day 28<br>Hospital discharge | 8<br>7 | 3298<br>1764 | 0.89 (0.82-0.97; NA)<br>1.06 (1.00-1.13; NA) | LOW: 0%<br>MED: 40.9% | S: 0.064<br>No association |
| 2021 | Khan | REM | PRISMA | High | Prospectives<br>Retrospectives | Standard treatment | Mortality | 20<br>40 | 6563 | 0.83 (0.72-0.96; NA)<br>0.52 (0.41-0.66; NA) | LOW: 0%<br>HIGH: 76.6% | S: 0.103<br>S: 0.361 |
| 2021 | Kyriakopoulos | REM | Cochrane | High | Observational<br><br>RCT | Standard treatment | Mortality<br>MV<br>Mortality<br>MV | 38<br>10<br>9<br>4 | 4456<br>717<br>3358<br>2568 | 0.69 (0.58-0.83; NA)<br>0.81 (0.57-1.14; NA)<br>0.89 (0.82-0.96; NA)<br>0.81 (0.71-0.93; NA) | HIGH: 84%<br>HIGH: 70.2%<br>LOW: 0.3%<br>LOW: 0% | S: 0.205<br>No association<br>S: 0.064<br>S: 0.116 |
| 2021 | Lin | REM | PRISMA<br>Cochrane<br>WHO | High | RCT | Standard treatment /PBO | Mortality<br>Clinical improvement | 8 | 3267 | 0.92 (0.66-1.28; NA) | MED: 62% | No association |
| 2021 | Moosazadeh | REM | Cochrane | High | RCT, Cohorts<br>Tocilizumab + corticosteroids | Standard treatment /PBO | Mortality VS Tocilizumab<br>Mortality VS Placebo | 5<br>5 | 460<br>567 | 0.74 (0.36-1.50; NA)<br>0.48 (0.31-0.74; NA) | MED: 72.5%<br>MED: 68.8% | No association<br>S: 0.405 |
| 2021 | Pinzón | REM | PRISMA | High | RCT<br>Case-control<br>Retrospectives | Standard treatment /PBO | Mortality<br>Hospital discharge<br>Reduct. days hospitalization | 17<br>13<br>4 | 1293<br>864<br>240 | 0.61 (0.49-0.76; NA)<br>1.04 (0.86-1.24; NA)<br>-1.96 (-4.24-0.33; NA) | MED: 58%<br>HIGH: 82%<br>MED: 41% | S: 0.273<br>S: 0.022<br>- |
| 2021 | Rezaei | REM | PRISMA,<br>Cochrane | High | RCT<br>Cohorts<br>Case-control | Standard treatment | Mortality (ECA)<br>Mortality (COH)<br>Mortality (CC)<br>Clinical improvement (RCT)<br>Idem (Cohort<br>Idem (Case-control) | 4<br>26<br>16<br>1<br>4<br>1 | 578<br>2661<br>760<br>-<br>-<br>- | 1.04 (0.73-1.48; 0,937)<br>0.82 (0.66-1.01; 0,000)<br>0.62 (0.47-0.82; NA)<br>1.19 (0.95-1.49; NA)<br>1.11 (0.87-1.43; 0,135)<br>1.45 (1.14-1.85; NA) | LOW: 0%<br>HIGH: 81.3%<br>MED: 66.5%<br>LOW: 0%<br>MED: 46.1%<br>LOW: 0% | No association<br>No association<br>S: 0.264<br>No association<br>No association<br>No association |

**Tabla S2.** Interleukin 6 (IL-6) inhibitors: Tocilizumab. Meta-analysis of its effectiveness in the treatment of COVID-19. Continuation.

| Year | First author | Meta-analysis type | PRISMA / Cochrane / WHO § | Quality NHLBI # | Clinical trials design | Comparator | Main efficacy endpoints | No studies included | No patients included | OR/RR/HR (95% CI; p) | Hetero-geneity ($I^2$) * | Magnitude of effect: Cohen's "d" ** |
|---|---|---|---|---|---|---|---|---|---|---|---|---|
| 2021 | Rubio-Rivas | REM | PRISMA | High | Observational<br>RCT | Standard treatment | Mortality | 64 | 7668 | 0.73 (0.56-0.93;<0.001) | HIGH: 82% | S: 0,174 |
| 2021 | Snow | REM | PRISMA, Cochrane | High | RCT | Standard treatment /PBO | Mortality days 28-30 | 9 | 3358 | 0.87 (0.74-1.01; 0.07) | LOW: 10% | No association |
| 2021 | Tharmarajah | REM/<br>NMA | PRISMA, Cochrane | High | RCT: Tocilizumab + Sarilumab | Standard treatment /PBO | Mortality day 28 | 9 | 3647 | 0.90 (0.81-0.99; NA) | LOW: 0% | S: 0,058 |
| 2021 | Tleyjeh | REM | Cochrane | High | RCT<br><br>Observational | Standard treatment /PBO | Mortality<br>Infection risk<br>Infection risk | 8<br>8<br>28 | 6311<br>5340<br>15484 | 0.91 (0.78-1.07; NA)<br>0.67 (0.45-0.99; NA)<br>0.53 (0.43-0.67; NA) | LOW: 25%<br>MED: 45%<br>HIGH: 76% | No association<br>S: 0,221<br>S: 0,350 |
| 2021 | Viswanatha | REM | PRISMA, Cochrane | High | Observational retrospective | Standard treatment /PBO | Mortality<br>ICU admission<br>MV | 22<br>8<br>12 | 1841<br>635<br>756 | -0.11 (-0.18; -0.04; NA)<br>-2.86 (-9.10; 3.38; NA)<br>0.00 (-0.06; 0.07; NA) | HIGH: 88%<br>HIGH: 100%<br>MED: 74% | No association<br>No association<br>No association |
| 2021 | Wei | REM | PRISMA, Cochrane | High | Observational<br>RCT | Standard treatment | Mortality | 13 | NA | 0.45 (0.24-0.81;0.01) | HIGH: 99% | S: 0,440 |
| 2021 | WHO | REM/<br>FEM | WHO | High | RCT | Standard treatment /PBO | Mortality day 28 | 19 | 4299 | 0.83 (0.74-0.92;<0.001) | ND | S: 0,103 |
| 2022 | Luo | REM | PRISMA, Cochrane | High | RCT | Standard treatment | Mortality days 28-30<br>Total Mortality<br>MV<br>Secondary infection | 9<br>9<br>6<br>8 | 3039<br>3991<br>2462<br>1336 | 0.91 (0.83-0.99; NA)<br>0.91 (0.84-0.98; NA)<br>0.81 (0.70-0.95; NA)<br>0.74 (0.50-1.08; NA) | LOW: 3%<br>LOW: 0%<br>LOW: 0%<br>MED: 42% | S: 0,052<br>S: 0,052<br>S: 0,116<br>No association |
| 2022 | Peng (a) | REM | PRISMA | High | RCT<br>Case-control | Standard treatment | Mortality<br>Secondary infection risk | 5<br>24 | 827<br>2224 | 0.74 (0.58-0.94;0.013)<br>1.12 (0.87-1.43;0.376) | MED: 61,5%<br>MED: 55,8% | S: 0,166<br>No association |
| 2022 | Peng (b) | REM/<br>FEM | PRISMA, Cochrane | High | RCT | Standard treatment | Mortality<br>Hospital discharge | 10<br>8 | 3378<br>3038 | 0.89 (0.82-0.96;0.003)<br>1.13 (1.08-1.18;<0.00001) | LOW: 28%<br>LOW: 34% | S: 0,064<br>S: 0,067 |
| 2022 | Vela | FEM | PRISMA, Cochrane | High | RCT | Standard treatment /PBO | Mortality all causes<br>MV | 10<br>9 | 3528<br>3042 | 0.88 (0.81-0.95; 0.0009)<br>0.79 (0.71-0.88; 0.0001) | LOW: 10%<br>LOW: 0% | S: 0,070<br>S: 0,130 |
| 2022 | Yu | REM | PRISMA | High | RCT | Standard treatment /PBO | Mortality day 28<br>Progression to MV | 12<br>8 | 7369<br>5392 | 0.89 (0.82-0.95; NA)<br>0.79 (0.71-0.88; NA) | LOW: 0%<br>LOW: 0% | S: 0,064<br>S: 0,130 |
| 2023 | Albuquerque | Bayesian model | PRISMA | High | RCT<br>(+ corticoids) | Standard treatment /PBO | Mortality day 28 | 16 | 3042 | 0.78 (0.65-0.94; NA) | NA | S: 0,137 |

*The following criteria were used: (i) up to 25%, low heterogeneity (LOW); (ii) between > 25% and < 75%, moderate heterogeneity (MED); and, finally, (iii) if ≥ 75%, high heterogeneity (HIGH) (*Higgins, 2003*); ** By convention, for Cohen's "d" of 0-0.4; 0.5-0.7 and ≥ 0.8 are considered small (S), medium (M) and large (L) effect sizes respectively (*Chen, 2010*). Cohen's d was calculated using the tool available at the following URL: https://www.escal.site/. § References: PRISMA: *Liberati, 2009; Moher, 2009; Page, 2021; Cochrane: Cochrane, 2022; WHO: Shankar-Hari, 2021*. # Reference: *National, 2023*; ¶ Non randomized prospective cohort studies. Abbreviations: 95% CI: 95% confidence interval; FEM: fixed effects model; HIGH: high heterogeneity; HR: hazard ratio; L: large effect; LOW: low heterogeneity; M: medium effect; MED: average heterogeneity; MV: mechanical ventilation; NHLBI: National Heart, Lung and Blood Institute (*National, 2023*); NA: not available; NMA: network meta-analysis; OR: odds ratio; p: statistical significance; PBO: placebo; RCT: randomized clinical trials; REM: random effects model; RR: relative risk; S: small effect.

**Tabla S3.** Interleukin 6 (IL-6) inhibitors: Sarilumab. Meta-analysis of its effectiveness in the treatment of COVID-19.

| Year | First author | Meta-analysis type | PRISMA / Cochrane / WHO § | Quality NHLBI # | Clinical trials design | Comparator | Main efficacy endpoints | No studies included | No patients included | OR/RR/HR (95% CI; p) | Hetero-geneity ($I^2$) * | Magnitude of effect: Cohen's "d" ** |
|---|---|---|---|---|---|---|---|---|---|---|---|---|
| 2021 | Ghosn | REM | Cochrane | High | RCT | Standard treatment/PBO | Mortality day 28 | 2 | 880 | 0.77 (0.43-1.36; NA) | MOD: 32.3% | No association |
| 2021 | Peng | REM | PRISMA | High | RCT<br>Case-control | Standard treatment | Mortality<br>Secondary infection | 1 | 28 | 0.40 (0.07-2.24; 0.297)<br>1.20 (0.33-4.39; 0.783) | NA<br>NA | No association<br>No association |
| 2021 | Tharmarajah | REM<br>NMA | PRISMA.<br>Cochrane | High | RCT: Tocilizumab + Sarilumab | Standard treatment/PBO | Mortality day 28 | 9 | 3647 | 0.90 (0.81-0.99; NA) | LOW: 0% | S: 0.058 |
| 2021 | WHO | FEM/ REM | WHO | High | RCT | Standard treatment/PBO | Mortality day 28 | 9 | 2073 | 1.08 (0.86-1.36;0.52) | LOW: 0% | No association |
| 2022 | Yu | REM | PRISMA | High | RCT | Standard treatment/PBO | Mortality day 28<br>VM progression | 6<br>1 | 1483<br>115 | 0.81 (0.59-1.10; NA)<br>1.15 (0.38-3.51; NA) | NA<br>NA | No association<br>No association |
| 2023 | Albuquerque | Bayesian | PRISMA | High | RCT<br>(+ corticoids) | Standard treatment/PBO | Mortality day 28 | 8 | 703 | 0.91 (0.60-1.40; NA) | NA | No association |

*The following criteria were used: (i) up to 25%, low heterogeneity (LOW); (ii) between > 25% and < 75%, moderate heterogeneity (MED); and, finally, (iii) if ≥ 75%, high heterogeneity (HIGH) (*Higgins, 2003*); ** By convention, for Cohen's "d" of 0-0.4; 0.5-0.7 and ≥ 0.8 are considered small (S), medium (M) and large (L) effect sizes respectively (*Chen, 2010*). Cohen's d was calculated using the tool available at the following URL: https://www.escal.site/. § References: PRISMA: *Liberati, 2009; Moher, 2009; Page, 2021; Cochrane: Cochrane, 2022; WHO: Shankar-Hari, 2021*. # Reference: *National, 2023*; ¶ Non randomized prospective cohort studies. Abbreviations: 95% CI: 95% confidence interval; FEM: fixed effects model; HIGH: high heterogeneity; HR: hazard ratio; L: large effect; LOW: low heterogeneity; M: medium effect; MED: average heterogeneity; MV: mechanical ventilation; NHLBI: National Heart, Lung and Blood Institute (*National, 2023*); NA: not available; NMA: network meta-analysis; OR: odds ratio; p: statistical significance; PBO: placebo; RCT: randomized clinical trials; REM: random effects model; RR: relative risk; S: small effect.

**Table S4 (a).** Monoclonal antibodies. Meta-analysis of its effectiveness in the treatment of COVID-19.

| Year | First author | Meta-analysis type | PRISMA / Cochrane / WHO § | Quality NHLBI # | Clinical trials design | Monoclonal antibody Comparator | COVID-19 variants results | Main efficacy endpoints | No studies included | No patients included | OR/RR/HR (95% CI; p) | Hetero-geneity (I²) * | Magnitude of effect: Cohen's "d" ** |
|---|---|---|---|---|---|---|---|---|---|---|---|---|---|
| 2021 | Siemieniuk | NMA | PRISMA, Cochrane | High | RCT | **Casirivimab-Imdevimab**<br>Standard treatment | NA | *Non-serious illness*:<br>Hospitalization risk<br>Mortality<br>MV<br>*Serious illness:*<br>Mortality | <br>4<br>4<br>4<br><br>4 | <br>NA<br>NA<br>NA<br><br>NA | <br>0.29 (0.17-0.47; NA)<br>0.58 (0.26-1.22; NA)<br>0.21 (0.02-1.20; NA)<br><br>0.94 (0.58-1.52; NA) | <br>NA<br>NA<br>NA<br><br>NA | <br>M: 0.682<br>No association<br>No association<br><br>No association |
| | | | | | | **Sotrovimab**<br>Standard treatment | | *Non-serious illness*:<br>Hospitalization risk<br>Mortality<br>MV | <br>1<br>1<br>1 | <br>NA<br>NA<br>NA | <br>0.17 (0.04-0.57; NA)<br>0.33 (0.01-10.16; NA)<br>0.20 (0.01-5.07; NA) | <br>NA<br>NA<br>NA | <br>L: 0.977<br>No association<br>No association |
| 2022 | Amani | REM<br>FEM | PRISMA | High | RCT<br>Observational | **Sotrovimab**<br>Standard treatment | Delta,<br>Omicron | Mortality<br>Hospitalization risk<br>MV | 15<br>13<br>6 | NA<br>NA<br>NA | 0.40 (0.25-0.63; 0.000)<br>0.53 (0.43-0.65; 0.000)<br>0.57 (0.33-0.96; 0.037) | NA<br>NA<br>NA | M: 0.505<br>S: 0.350<br>S: 0.310 |
| 2022 | McConnell | NMA | PRISMA, Cochrane | High | RCT | **Casirivimab-Imdevimab**<br>Placebo vs antibody | NA | Mortality<br>Hospitalization risk<br>MV | 1<br>1<br>1 | NA<br>NA<br>NA | 7.69 (1.77-40.63;<0.05)<br>3.70 (2.21-6.22;<0.05);<br>4.96 (1.10-32.38;<0.05) | NA<br>NA<br>NA | L: 1.125<br>M: 0.721<br>L: 0.083 |
| | | | | | | **Sotrovimab**<br>Placebo vs antibody | | Mortality<br>Hospitalization risk<br>MV | 2<br>2<br>2 | NA<br>NA<br>NA | 13.70 (0.75-842.5;<0.05)<br>4.95 (180-14.86;<0.05)<br>20.61 (1.36-1147;<0.05) | NA<br>NA<br>NA | L: 1.443<br>L: 0.082<br>L: 1.668 |
| 2022 | Yang*** | NA | Cochrane | High | RCT<br>Observational | **Regdanvimab** | NA | Mortality | 4 | 789 | 0.14 (0.03-0.56;0.006) | LOW: 0% | L: 1.084 |
| 2023 | Deng | NMA | PRISMA, Cochrane | High | RCT | **Casirivimab-Imdevimab**<br>Standard treatm/PBO | NA | Mortality<br>Hospitalization risk | NA<br>NA | NA<br>NA | 0.67 (0.50-0.91; NA)<br>0.29 (0.20-0.42; NA) | NA<br>NA | S: 0.221<br>M: 0.682 |
| | | | | | | **Sotrovimab**<br>Standard treatm/PBO | | Mortality | NA | NA | 0.20 (0.08-0.48; NA) | NA | L: 0.887 |

*The following criteria were used: (i) up to 25%, low heterogeneity (LOW); (ii) between > 25% and < 75%, moderate heterogeneity (MED); and, finally, (iii) if ≥ 75%, high heterogeneity (HIGH) (*Higgins, 2003*); ** By convention, for Cohen's "d" of 0-0.4; 0.5-0.7 and ≥ 0.8 are considered small (S), medium (M) and large (L) effect sizes respectively (*Chen, 2010*). Cohen's d was calculated using the tool available at the following URL: https://www.escal.site/. § References: PRISMA: *Liberati, 2009; Moher, 2009; Page, 2021; Cochrane: Cochrane, 2022; WHO: Shankar-Hari, 2021*. # Reference: *National, 2023*; ¶ Non randomized prospective cohort studies. Abbreviations: 95% CI: 95% confidence interval; FEM: fixed effects model; HIGH: high heterogeneity; HR: hazard ratio; L: large effect; LOW: low heterogeneity; M: medium effect; MED: average heterogeneity; MV: mechanical ventilation; NHLBI: National Heart, Lung and Blood Institute (*National, 2023*); NA: not available; NMA: network meta-analysis; OR: odds ratio; p: statistical significance; PBO: placebo; RCT: randomized clinical trials; REM: random effects model; RR: relative risk; S: small effect.

**Table S4 (b).** Clinical studies on mortality published in the years 2022 and 2023 (until October) of the treatment of COVID-19 with cilgavimab/tixagevimab (PubMed).

| Year | Author | Study type | Patients | Results by COVID-19 variants | Arms | N | Mortality | HR, RR (IC 95%) | P |
|---|---|---|---|---|---|---|---|---|---|
| 2022 | ACTIV-3 | RCT | COVID-19<br>Hospitalized | NA | Treated<br>Placebo | 710<br>707 | 9%<br>12% | 0.70 (0.50;0.97) | 0.032 |
| 2023 | Kertes | Cohorts | COVID-19<br>Immunocompromised | NA | Treated<br>Untreated | 825<br>4299 | 0%<br>0,9% | - | 0.005 |
| 2023 | Wang | Meta-analysis | COVID-19 | NA | Treated<br>Placebo | 5383<br>7152 | - | 0.50 (0.39;0.64) | <0.01 |

RCT: randomized clinical trials; HR: hazard ratio; N: sample size; NA: not available; RR: relative risk.

**Table S5.** Hydroxychloroquine and chloroquine. Meta-analysis of its effectiveness in the treatment of COVID-19.

| Year | First author | Meta-analysis type | PRISMA / Cochrane / WHO § | Quality NHLBI # | Clinical trials design | Comparator | Main efficacy endpoints | No studies included | No patients included | OR/RR/HR (95% CI; p) | Hetero-geneity (I²) * | Magnitude of effect: Cohen's "d" ** |
|---|---|---|---|---|---|---|---|---|---|---|---|---|
| 2020 | Elavarasi | REM | PRISMA, Cochrane | High | Observational<br>RCT | Standard treatment | Mortality<br>MV | 7<br>6 | 4295<br>2071 | 0.98 (0.66-1.46; NA)<br>0.90 (0.47-1.71; NA) | HIGH: 87%<br>HIGH: 87% | No association<br>No association |
| 2020 | Ghazy | REM | PRISMA, Cochrane | High | Observational<br>RCT | Standard treatment/PBO | Mortality | 8 | 1671 | 0.99 (0.61-1.59; 0.96) | HIGH: 82% | No association |
| 2020 | Kashour | REM | PRISMA, Cochrane | High | RCT<br>Cohorts | Standard treatment/PBO | Mortality | 13 | - | 0.93 (0.79-1.11; NA) | MED:59% | No association |
| 2020 | Pathak | REM | Cochrane | High | RCT | Standard treatment | Ni Mortality ni VM | 7 | 1721 | 1.11 (0.72-1.69; NA) | MED: 32% | No association |
| 2020 | Sarma | REM | PRISMA, Cochrane | High | Observational<br>RCT | Standard treatment | Virological cure<br>Mortality/deterioration | 2<br>3 | 29<br>66 | 2.37 (0.13-44.53; NA)<br>1.37 (0.09-21.97; NA) | MED: 72%<br>MED: 59% | No association<br>No association |
| 2020 | Singh | REM | PRISMA, Cochrane | High | Observational<br>RCT | Standard treatment/PBO | Negative PCR<br>Mortality | 3<br>3 | 210<br>474 | 1.05 (0.79-1.38; 0.74)<br>2.17 (1.32-3.56; 0.002) | MED: 61.7%<br>LOW: 0% | No association<br>S: 0.427 |
| 2021 | Amani | REM | PRISMA, Cochrane | High | RCT | Standard treatment/PBO | Negative PCR<br>Mortality<br>MV<br>Disease progression | 4<br>4<br>4<br>4 | 460<br>2764<br>2503<br>465 | 0.99 (0.90-1.08; NA)<br>1.09 (1.00-1.20; NA)<br>1.12 (0.95-1.32; NA)<br>0.82 (0.37-1.85; NA) | LOW: 0%<br>LOW: 0%<br>LOW: 0%<br>LOW: 11% | No association<br>No association<br>No association<br>No association |
| 2021 | Axfors | REM | PRISMA, Cochrane WHO | High | RCT | Standard treatment/PBO | Dead Hydroxychloroquine<br>Dead Chloroquine | 14<br>5 | 3809<br>160 | 1.12 (1.08-1.16; NA)<br>1.77 (0.15-21.13; NA) | LOW: 0%<br>LOW: 0% | No association<br>No association |
| 2021 | Celotto | REM | PRISMA | High | Metaanálisis | PBO | Mortality | 6 | NA | Higher risk of death | NA | - |
| 2021 | Chi | NMA | Cochrane | High | RCT | Standard treatment/PBO | Mortality<br>MV | NA<br>NA | NA<br>NA | 1.12 (0.99-1.26; NA)<br>1.12 (0.94-1.34; NA) | NA<br>NA | No association<br>No association |
| 2021 | Chivese | REM | PRISMA, Cochrane | High | Metaanálisis | Standard treatment/PBO | Mortality | NA | NA | 1.1 (1.0-1.3; NA) | LOW: 0% | No association |
| 2021 | Elsawah | REM/ FEM | PRISMA, Cochrane | High | RCT<br>Observational | Standard treatment | Mortality 5 días | 4 | 249 | 0.01 (-0.01;0.03; NA) | LOW: 0% | No association |
| 2021 | Fiolet | REM | PRISMA, Cochrane | High | RCT<br>Observational | Standard treatment | Mortality | 17 | NA | 0.83 (0.65-1.06; NA) | HIGH: 83% | No association |
| 2021 | García-Albéniz | REM/ FEM | PRISMA, Cochrane | High | RCT | Standard treatment/PBO | Pre-exposure prophylaxis<br>Post-exposure prophylaxis | 7<br>4 | NA<br>NA | 0.72 (0.58-0.90; NA)<br>0.91 (0.72-1.16; NA) | LOW: 0%<br>LOW: 0% | S: 0.181<br>No association |

**Table S5.** Hydroxychloroquine and chloroquine. Meta-analysis of its effectiveness in the treatment of COVID-19. Continuation.

| Year | First author | Meta-analysis type | PRISMA / Cochrane / WHO § | Quality NHLBI # | Clinical trials design | Comparator | Main efficacy endpoints | No studies included | No patients included | OR/RR/HR (95% CI; p) | Hetero-geneity ($I^2$) * | Magnitude of effect: Cohen's "d" ** |
|---|---|---|---|---|---|---|---|---|---|---|---|---|
| 2021 | Singh (a) | REM | Cochrane | High | RCT | Standard treatment/PBO | Mortality<br>MV | 9<br>3 | 8208<br>4521 | 1.09 (0.99-1.19; ND)<br>1.11 (0.91-1.37; ND) | LOW: 0%<br>LOW: 0% | No association<br>No association |
| 2022 | Gupta | REM | PRISMA, Cochrane | High | RCT | Standard treatment/PBO | Clinical improvement day 28<br>Mortality day 28 | 9<br>14 | 1606<br>3788 | 1.00 (0.96-1.03; ND)<br>1.08 (0.99-1.19; ND) | MED: 51%<br>LOW: 0% | No association<br>No association |

*The following criteria were used: (i) up to 25%, low heterogeneity (LOW); (ii) between > 25% and < 75%, moderate heterogeneity (MED); and, finally, (iii) if ≥ 75%, high heterogeneity (HIGH) (*Higgins, 2003*); ** By convention, for Cohen's "d" of 0-0.4; 0.5-0.7 and ≥ 0.8 are considered small (S), medium (M) and large (L) effect sizes respectively (*Chen, 2010*). Cohen's d was calculated using the tool available at the following URL: https://www.escal.site/. § References: PRISMA: *Liberati, 2009; Moher, 2009; Page, 2021; Cochrane: Cochrane, 2022; WHO: Shankar-Hari, 2021*. # Reference: *National, 2023*; ¶ Non randomized prospective cohort studies. Abbreviations: 95% CI: 95% confidence interval; FEM: fixed effects model; HIGH: high heterogeneity; HR: hazard ratio; L: large effect; LOW: low heterogeneity; M: medium effect; MED: average heterogeneity; MV: mechanical ventilation; NHLBI: National Heart, Lung and Blood Institute (*National, 2023*); NA: not available; NMA: network meta-analysis; OR: odds ratio; p: statistical significance; PBO: placebo; RCT: randomized clinical trials; REM: random effects model; RR: relative risk; S: small effect.

**Table S6 (a).** Antivirals: Remdesivir. Meta-analysis of its effectiveness in the treatment of COVID-19.

| Year | First author | Meta-analysis type | PRISMA / Cochrane / WHO § | Quality NHLBI # | Clinical trials design | Comparator | Main efficacy endpoints | No studies included | No patients included | OR/RR/HR (95% CI; p) | Hetero-geneity ($I^2$) * | Magnitude of effect: Cohen's "d" ** |
|---|---|---|---|---|---|---|---|---|---|---|---|---|
| 2021 | Jiang | NMA | Cochrane | High | RCT | 10 days vs PBO<br>5 days vs PBO | Clinical improvement day 10-15***<br>Clinical recovery follow-up | 3<br>1 | 779<br>191 | 1.35 (1.09-1.67; NA)<br>1.81 (1.32-2.45; NA) | NA<br>NA | S: 0.165<br>S: 0.327 |
| 2021 | Lai | NMA | PRISMA,<br>Cochrane | High | RCT | 5 days vs PBO/TE<br><br>10 days vs PBO/TE | Clinical improvement<br>Mortality<br>Clinical improvement<br>Mortality | 3<br>4<br>3<br>4 | ND<br>ND<br>ND<br>ND | 1.68 (1.18-2.40; NA)<br>0.50 (0.24-1.04; NA)<br>1.23 (0.90-1.68; NA)<br>0.74 (0.54-1.03; NA) | LOW: 0%<br>LOW: 0%<br>LOW: 0%<br>LOW: 0% | S: 0.286<br>No association<br>No association<br>No association |
| 2021 | Shrestha | REM<br>FEM | PRISMA | High | RCT | Standard treatment/PBO | Clinical improvement day 14<br>Clinical improvement day 28<br>Mortality day 14<br>Mortality day 28<br>Clinical recovery day 14<br>Clinical recovery day 28 | 2<br>2<br>3<br>2<br>2<br>1 | 351<br>351<br>889<br>351<br>487<br>178 | 1.45 (1.00-2.08; 0.05)<br>1.59 (1.06-2.39; 0.02)<br>0.61 (0.41-0.91; 0.01)<br>1.02 (0.50-2.06; 0.96)<br>1.48 (1.19-1.84; 0.00)<br>2.09 (1.09-4.03; 0.03) | LOW: 0%<br>LOW: 0%<br>LOW: 0%<br>LOW: 0%<br>LOW: 0%<br>- | No association<br>S: 0.256<br>S: 0.273<br>No association<br>S: 0.216<br>L: 0.406 |
| 2021 | Singh (b) | REM<br>FEM | PRISMA,<br>Cochrane | High | RCT | Standard treatment | Clinical improvement<br>Mortality<br>Time to clinical improvement | 3<br>1<br>2 | 1080<br>3818<br>ND | 1.52 (1.24-1.87;<0.0001)<br>0.92 (0.79-1.07;0.3)<br>1.28 (1.12-1.46;0.0002) | LOW: 0%<br>LOW: 0%<br>LOW: 0% | S: 0.231<br>No association<br>S: 0.136 |
| 2022 | Angamo | FEM | PRISMA,<br>Cochrane | High | RCT<br>Observational | Standard treatment/PBO | Clinical recovery day 7<br>Clinical recovery day 14<br>Clinical recovery day 28 | 4<br>5<br>4 | 1080<br>1392<br>1083 | 1.21 (1.08-1.35;0.0008)<br>1.29 (1.22-1.37;0.00001)<br>1.09 (1.04-1.14;0.0002) | MED: 29%<br>HIGH: 76%<br>LOW: 0% | S: 0.105<br>S: O.140<br>S: 0.048 |
| 2022 | Beckerman | NMA | PRISMA | High | RCT | Standard treatment/PBO | Early mortality<br>Late mortality<br>Early clinical recovery<br>Late clinical recovery | 6<br>6<br>6<br>6 | 924<br>2791<br>2752<br>2783 | 0.52 (0.34-0.79; NA)<br>0.81 (0.69-0.95; NA)<br>1.01 (0.97-1.06; NA)<br>1.03 (1.00-1.06; NA) | NA<br>NA<br>NA<br>NA | S: 0.361<br>S: 0.116<br>No association<br>No association |
| 2022 | Kaka | FEM | Cochrane | High | RCT | 10 días vs PBO/TE<br>10 días vs PBO/TE | Mortality<br>MV | 4<br>3 | 3635<br>887 | 0.93 (0.82-1.06; NA)<br>0.71 (0.56-0.90; NA) | LOW: 6%<br>MED: 38% | No association<br>S: 0.189 |
| 2022 | Lee | REM | PRISMA,<br>Cochrane | High | RCT | Standard treatment/PBO | Mortality with MV<br>Mortality w/o MV<br>Mortality w/o oxygen | 4<br>7<br>5 | 504<br>3689<br>1339 | 1.08 (0.88-1.31;0.353)<br>0.89 (0.79-0.99;0.363)<br>0.77 (0.50-1.19;0.420) | LOW: 8%<br>LOW: 8.6%<br>LOW: 2.9% | No association<br>No association<br>No association |
| 2022 | WHO**** | RCT | ND | ND | RCT | Standard treatment | Mortality | ND | 4146 | 0.91 (0.82-1.02;0.12) | NA | No association |

**Table S6 (a).** Antivirals: Remdesivir. Meta-analysis of its effectiveness in the treatment of COVID-19. Continuation.

| Year | First author | Meta-analysis type | PRISMA / Cochrane / WHO § | Quality NHLBI # | Clinical trials design | Comparator | Main efficacy endpoints | No studies included | No patients included | OR/RR/HR (95% CI; p) | Hetero-geneity ($I^2$) * | Magnitude of effect: Cohen's "d" ** |
|---|---|---|---|---|---|---|---|---|---|---|---|---|
| 2023 | Grundeis | REM | Cochrane | High | RCT | Standard treatment/ PBO | Mortality day 28<br>Mortality day 60<br>In-hospital mortality day 150Clinical improvement day 28<br>Clinical worsening day 28 | 4<br>1<br>1<br>4<br>2 | 7142<br>1281<br>8275<br>2514<br>1734 | 0.93 (0.81-1.06; 0.28)<br>0.85 (0.69-1.05; 0.13)<br>0.93 (0.84-1.03; 0.18)<br>1.11 (1.06-1.17;0.0001)<br>0.67 (0.54-0.82;<0.0001) | LOW: 0%<br>-<br>-<br>LOW: 0%<br>LOW: 0% | No association<br>No association<br>No association<br>No association<br>S: 0.221 |

*The following criteria were used: (i) up to 25%, low heterogeneity (LOW); (ii) between > 25% and < 75%, moderate heterogeneity (MED); and, finally, (iii) if ≥ 75%, high heterogeneity (HIGH) (*Higgins, 2003*); ** By convention, for Cohen's "d" of 0-0.4; 0.5-0.7 and ≥ 0.8 are considered small (S), medium (M) and large (L) effect sizes respectively (*Chen, 2010*). Cohen's d was calculated using the tool available at the following URL: https://www.escal.site/. § References: PRISMA: *Liberati, 2009; Moher, 2009; Page, 2021; Cochrane: Cochrane, 2022; WHO: Shankar-Hari, 2021*. # Reference: *National, 2023*; ¶ Non randomized prospective cohort studies. Abbreviations: 95% CI: 95% confidence interval; FEM: fixed effects model; HIGH: high heterogeneity; HR: hazard ratio; L: large effect; LOW: low heterogeneity; M: medium effect; MED: average heterogeneity; MV: mechanical ventilation; NHLBI: National Heart, Lung and Blood Institute (*National, 2023*); NA: not available; NMA: network meta-analysis; OR: odds ratio; p: statistical significance; PBO: placebo; RCT: randomized clinical trials; REM: random effects model; RR: relative risk; S: small effect.

**Table S6 (b).** Mortality clinical studies published in 2023 (through October) of COVID-19 treatment with remdesivir (PubMed).

| Year | Author | Study type | Patients | Treatments | N | Mortality | OR, HR, RR (95% CI) | P |
|---|---|---|---|---|---|---|---|---|
| 2023 | Amstutz | RCT meta-analysis | COVID-19 Hospitalized | Remdesivir<br>Placebo/Standard | 5398<br>5082 | All causes day 28<br>All causes day 60 | OR=0.88 (0.78;1.00)<br>OR=0.91 (0.81;1.02) | 0.045<br>0.116 |
| 2023 | Huang | RCT meta-analysis | COVID-19 (w/o $O_2$ supplement) | Remdesivir<br>Placebo/ Standard | 2230<br>2151 | All causes day 28 | RR= 0.83 (0.71;0.98) | 0.02 |
| 2023 | Marx | Retrospective cohorts | COVID-19 Hospitalized | Remdesivir + corticoids<br>Only corticoids | 260<br>260 | All causes day 28 | HR= 0.60 (0.39;0.95) | 0.03 |
| 2023 | Metchurt-chlishvili | Prospective cohorts | COVID-19 Hospitalized | Remdesivir<br>Placebo/Standard | 346<br>346 | All causes | 2.3%<br>5.2% | 0.046 |
| 2023 | Mozaffari (a) | Retrospective cohorts | COVID-19 Hospitalized | Remdesivir (LOF)<br>Remdesivir (NIV)<br>Remdesivir (IV)<br>No remdesivir (LOF)<br>No remdesivir (NIV)<br>No remdesivir (IV) | 67582<br>34857<br>4164<br>10830<br>10189<br>1880 | All causes day 14<br>All causes day 14<br>All causes day 14<br>All causes day 28<br>All causes day 28<br>All causes day 28 | HR= 0.72 (0.66;0.79)<br>HR= 0.83 (0.77;0.89)<br>HR= 0.73 (0.65;0.82)<br>HR= 0.79 (0.73;0.85)<br>HR= 0.88 (0.82;0.93)<br>HR= 0.74 (0.67;0.82) | - |
| 2023 | Mozaffari (b) | Retrospective cohorts | COVID-19 Immunocompromised | Remdesivir<br>No remdesivir | 19184<br>11213 | All causes day 14<br>All causes day 28 | HR= 0.70 (0.62;0.78)<br>HR= 0.75 (0.68;0.83) | - |

RCT: randomized clinical trials; HR: hazard ratio; IV: invasive ventilation; LOF: low oxygen flow; N: sample size; NIV: non-invasive ventilation; RR: relative risk.

**Table S7.** Antivirals: Favipiravir. Meta-analysis of its effectiveness in the treatment of COVID-19.

| Year | First author | Meta-analysis type | PRISMA / Cochrane / WHO § | Quality NHLBI # | Clinical trials design | Comparator | Main efficacy endpoints | No studies included | No patients included | OR/RR/HR (95% CI; p) | Hetero-geneity ($I^2$) * | Magnitude of effect: Cohen's "d" ** |
|---|---|---|---|---|---|---|---|---|---|---|---|---|
| 2020 | Shrestha | REM<br>FEM | PRISMA, Cochrane | High | RCT<br>Observational | Other antivirals<br>Standard treatment | Viral elimination day 7<br>Viral elimination day 14<br>Clinical improvement day 7<br>Clinical improvement day 14 | 3<br>3<br>3<br>3 | 84<br>84<br>160<br>84 | 1.13 (0.55-2.33; 0.73)<br>1.06 (0.84-1.33; 0.65)<br>1.25 (1.01-1.53; 0.04)<br>1.29 (1.08-1.54; 0.005) | HIGH: 84%<br>MED: 67%<br>LOW: 0%<br>LOW: 16% | No association<br>No association<br>S: 0.123<br>S: 0.140 |
| 2021 | Hassanipour | REM | PRISMA | High | RCT | Standard treatment | Viral elimination<br>Clinical improvement | 6<br>8 | ND<br>ND | 1.06 (0.98-1.14; ND)<br>1.17 (1.07-1.28; ND) | ND<br>ND | No association<br>No association |
| 2021 | Özlüşen | REM | PRISMA, Cochrane | High | RCT<br>Observational | Standard treatment | Mortality<br>MV | 12<br>8 | 823<br>619 | 1.11 (0.64-1.94;0.69)<br>0.50 (0.13-1.95;<0.01) | LOW: 0%<br>HIGH: 75% | No association<br>S: 0.382 |
| 2022 | Hung | FEM | PRISMA, Cochrane | High | RCT<br>Observational | Standard treatment | Viral elimination day 5<br>Viral elimination day 10<br>Clinical improvement day 10 | 7<br>7<br>8 | 246<br>310<br>881 | 1.75 (1.11-2.77;0.02)<br>1.19 (1.06-1.34;<0.01)<br>1.27 (0.88-1.85;0.17) | MED: 54%<br>MED: 41%<br>HIGH: 73% | S: 0.309<br>S: 0.096<br>No association |

*The following criteria were used: (i) up to 25%, low heterogeneity (LOW); (ii) between > 25% and < 75%, moderate heterogeneity (MED); and, finally, (iii) if ≥ 75%, high heterogeneity (HIGH) (*Higgins, 2003*); ** By convention, for Cohen's "d" of 0-0.4; 0.5-0.7 and ≥ 0.8 are considered small (S), medium (M) and large (L) effect sizes respectively (*Chen, 2010*). Cohen's d was calculated using the tool available at the following URL: https://www.escal.site/. § References: PRISMA: *Liberati, 2009; Moher, 2009; Page, 2021; Cochrane: Cochrane, 2022; WHO: Shankar-Hari, 2021*. # Reference: *National, 2023*; ¶ Non randomized prospective cohort studies. Abbreviations: 95% CI: 95% confidence interval; FEM: fixed effects model; HIGH: high heterogeneity; HR: hazard ratio; L: large effect; LOW: low heterogeneity; M: medium effect; MED: average heterogeneity; MV: mechanical ventilation; NHLBI: National Heart, Lung and Blood Institute (*National, 2023*); NA: not available; NMA: network meta-analysis; OR: odds ratio; p: statistical significance; PBO: placebo; RCT: randomized clinical trials; REM: random effects model; RR: relative risk; S: small effect.

**Table S8.** Antivirals: Nirmatrelvir-Ritonavir. Meta-analysis of its effectiveness in the treatment of COVID-19.

| Year | First author | Meta-analysis type | PRISMA / Cochrane / WHO § | Quality NHLBI # | Clinical trials design | Comparator | Main efficacy endpoints | No studies included | No patients included | OR/RR/HR (95% CI; p) | Hetero-geneity ($I^2$) * | Magnitude of effect: Cohen's "d" ** |
|---|---|---|---|---|---|---|---|---|---|---|---|---|
| 2022 | Reis | REM | Cochrane | High | RCT | Standard treatment/PBO | Mortality day 28<br>Deterioration day 28 | 1<br>1 | 2224<br>1109 | 0.04 (0.00-0.68;0.03)<br>0.13 (0.07-0.27;<0.00001) | -<br>- | L: 1.775<br>L: 1.125 |
| 2023 | Amani | REM<br>FEM | PRISMA, Cochrane | High | RCT<br>Observational | Standard treatment/PBO | Mortality<br>Hospitalization | 13<br>11 | NA<br>NA | 0.25 (0.14-0.45; 0.000)<br>0.41 (0.24-0.69; 0.001) | NA<br>NA | M: 0.787<br>M: 0.492 |
| 2023 | Cheema | REM | Cochrane | High | RCT<br>Observational | No treatment/ PBO | Mortality<br>Hospitalization | 10<br>11 | NA<br>NA | 0.24 (0.15-0.39;<0.00001)<br>0.41 (0.29-0.59;<0.00001) | MED: 48%<br>HIGH: 90% | M: 0.787<br>M: 0.492 |

*The following criteria were used: (i) up to 25%, low heterogeneity (LOW); (ii) between > 25% and < 75%, moderate heterogeneity (MED); and, finally, (iii) if ≥ 75%, high heterogeneity (HIGH) (*Higgins, 2003*); ** By convention, for Cohen's "d" of 0-0.4; 0.5-0.7 and ≥ 0.8 are considered small (S), medium (M) and large (L) effect sizes respectively (*Chen, 2010*). Cohen's d was calculated using the tool available at the following URL: https://www.escal.site/. § References: PRISMA: *Liberati, 2009; Moher, 2009; Page, 2021; Cochrane: Cochrane, 2022; WHO: Shankar-Hari, 2021*. # Reference: *National, 2023*; ¶ Non randomized prospective cohort studies. Abbreviations: 95% CI: 95% confidence interval; FEM: fixed effects model; HIGH: high heterogeneity; HR: hazard ratio; L: large effect; LOW: low heterogeneity; M: medium effect; MED: average heterogeneity; MV: mechanical ventilation; NHLBI: National Heart, Lung and Blood Institute (*National, 2023*); NA: not available; NMA: network meta-analysis; OR: odds ratio; p: statistical significance; PBO: placebo; RCT: randomized clinical trials; REM: random effects model; RR: relative risk; S: small effect.

**Table S9.** Other antivirals. Meta-analysis of its effectiveness in the treatment of COVID-19.

| Year | First author | Meta-analysis type | PRISMA / Cochrane / WHO § | Quality NHLBI # | Clinical trials design | Comparator | Main efficacy endpoints | No studies included | No patients included | OR/RR/HR (95% CI; p) | Hetero-geneity ($I^2$) * | Magnitude of effect: Cohen's "d" ** |
|---|---|---|---|---|---|---|---|---|---|---|---|---|
| 2020 | Huang | REM | PRISMA, Cochrane | High | RCT<br>Observational | **Umifenovir**<br>Standard treatment | Negative PCR | 5 | 140 | 1.27 (1.04-1.55;0.02) | MED: 63% | S: 0.132 |
| 2021 | Wu | REM | Cochrane | High | RCT<br>Observational | **Lopinavir/ritonavir**<br>Standard treatment | Mortality<br>Viral elimination | 12<br>5 | 542<br>288 | 0.43 (0.25-0.73;0.002)<br>2.39 (1.68-3.39;<0.00001) | LOW: 0%<br>HIGH: 83% | M: 0.465<br>M: 0.480 |
| 2021 | Yu | FEM | PRISMA, Cochrane | High | RCT | **Umifenovir**<br>**Lopinavir/ritonavir** | Mortality | 3 | 84 | 0.32 (0.10-1.09;0.07) | LOW: 0% | No association |
| 2022 | Chen | REM<br>FEM | PRISMA, Cochrane | High | RCT | **INF-b**<br>Standard treatment | Mortality | 8 | 2488 | 0.74 (0.44-1.24;0.25) | MED: 51% | No association |
| 2022 | Okoli<br>*** | NMA | PRISMA, Cochrane | High | RCT | **Darunavir/cobicistat**<br>Standard treatment | Mortality<br>Clinical improvement<br>Viral elimination | NA | NA | 1.00 (0.02-5.10; NA)<br>0.94 (0.66-1.26; NA)<br>1.40 (0.25-4.57; NA) | NA | No association<br>No association<br>No association |
| | | | | | | **IFN-alpha**<br>Standard treatment | Mortality<br>Clinical improvement<br>Viral elimination | NA | NA | 1.12 (0.94-1.33; NA)<br>NA<br>NA | NA | No association<br>-<br>- |
| | | | | | | **INF-beta**<br>Standard treatment | Mortality<br>Clinical improvement<br>Viral elimination | NA | NA | 0.43 (0.08-1.18; NA)<br>1.16 (0.80-1.63; NA)<br>NA | NA | No association<br>No association<br>- |
| | | | | | | **Lopinavir/ritonavir**<br>Standard treatment | Mortality<br>Clinical improvement<br>Viral elimination | NA | NA | 0.95 (0.78-1.15; NA)<br>1.07 (0.81-1.40; NA)<br>1.06 (0.74-1.50; NA) | NA | No association<br>No association<br>No association |
| | | | | | | **Ribavirin**<br>Standard treatment | Mortality<br>Clinical improvement<br>Viral elimination | NA | NA | 0.73 (0.06-2.71; NA)<br>NA<br>0.72 (0.40-1.14; NA) | NA | No association<br>-<br>No association |
| 2023 | Buchynskyi | REM<br>FEM | PRISMA, Cochrane | High | RCT<br>Observational | **IFN-alpha**<br>Standard treatment | Mortality | 9 | NA | 0.25 (0.05-1.19; 0.082) | NA | No association |

*The following criteria were used: (i) up to 25%, low heterogeneity (LOW); (ii) between > 25% and < 75%, moderate heterogeneity (MED); and, finally, (iii) if ≥ 75%, high heterogeneity (HIGH) (*Higgins, 2003*); ** By convention, for Cohen's "d" of 0-0.4; 0.5-0.7 and ≥ 0.8 are considered small (S), medium (M) and large (L) effect sizes respectively (*Chen, 2010*). Cohen's d was calculated using the tool available at the following URL: https://www.escal.site/. § References: PRISMA: *Liberati, 2009; Moher, 2009; Page, 2021; Cochrane: Cochrane, 2022; WHO: Shankar-Hari, 2021*. # Reference: *National, 2023*; ¶ Non randomized prospective cohort studies. Abbreviations: 95% CI: 95% confidence interval; FEM: fixed effects model; HIGH: high heterogeneity; HR: hazard ratio; L: large effect; LOW: low heterogeneity; M: medium effect; MED: average heterogeneity; MV: mechanical ventilation; NHLBI: National Heart, Lung and Blood Institute (*National, 2023*); NA: not available; NMA: network meta-analysis; OR: odds ratio; p: statistical significance; PBO: placebo; RCT: randomized clinical trials; REM: random effects model; RR: relative risk; S: small effect.

## SUPLEMENT 6. SUPPLEMENTARY FIGURES

**Figure S1.** Effects on mortality from COVID-19 by number of published meta-analyses.

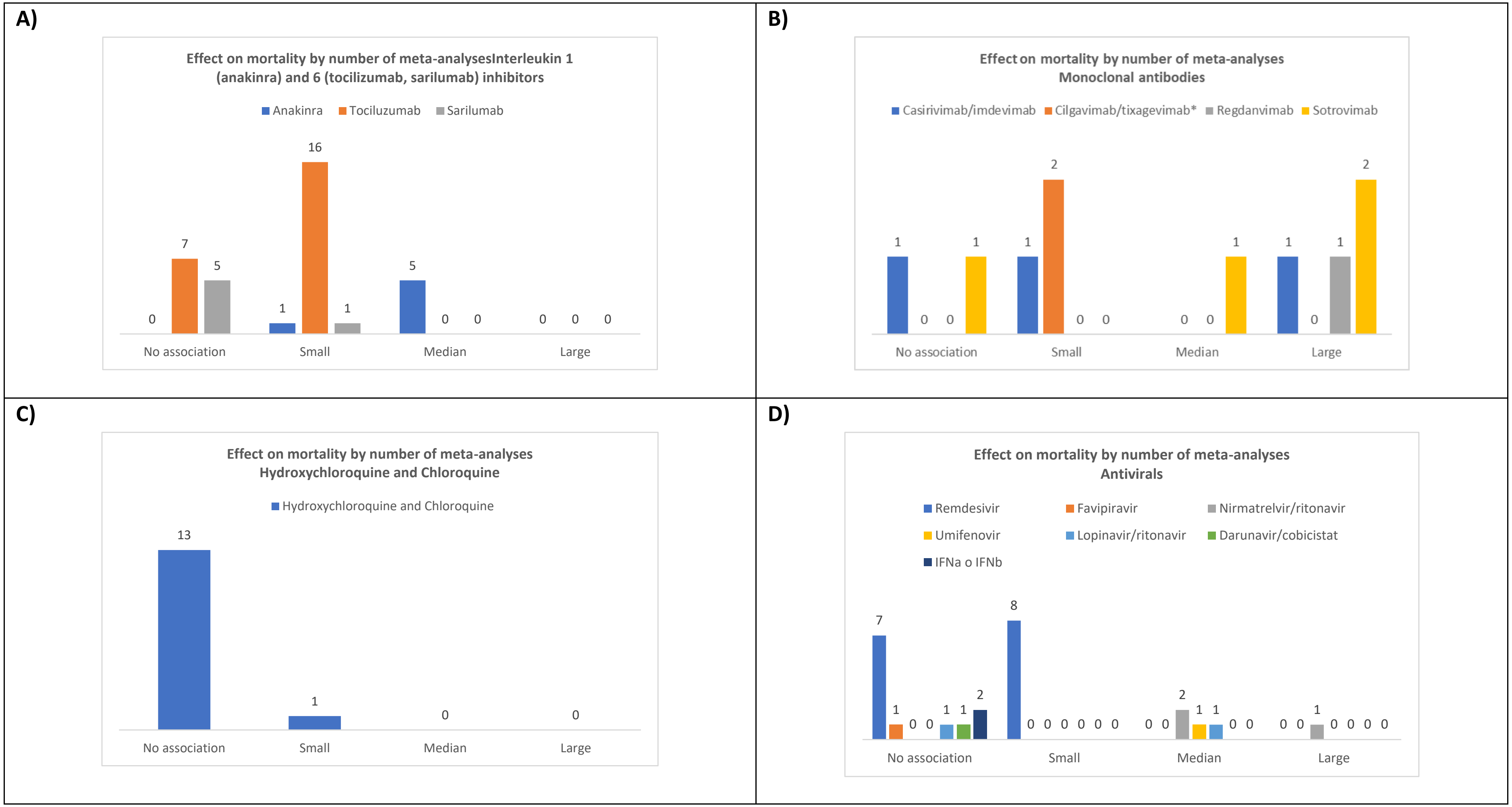

# SUPLEMENT 7.

**SUPPLEMENT 7. STUDIES EXCLUDED IN THE BASE CASE FOR BEING PUBLISHED IN JOURNALS WITH LOW IMPACT FACTOR (< 1)**

| Drug | Year | First author | Guidelines | Main efficacy endpoint | Studies design | N patients exp. Drug | OR/RR/HR (95% CI; p) | Magnitude of effect: Cohen's d* |
|---|---|---|---|---|---|---|---|---|
| ***Immunomodulators*** | | | | | | | | |
| Immunomodulators | 2020 | Talaie | PRISMA | Mortality | OBS, RCT | 271 | 0.22 (0.09;0.53) | L: 0.835 |
| Anakinra | 2022 | Malik | PRISMA | Mortality | OBS, RCT | 565 | 0.47 (0.37;0.59) | S: 0.416 |
| Anakinra | 2022 | Naveed | PRISMA | Mortality | OBS, RCT | 917 | 0.64 (0.49;0.83;<0.001) | S: 0.246 |
| Anakinra | 2023 | Dahms | - | Mortality | RCT | 888 | 0.96 (0.64;1.45) | No association |
| Anti-IL6 (tocilizumab, sarilumab) | 2022 | Alegre | PRISMA | Mortality | RCT | 6,349§ | 0.85 (0.74;0.99) | S: 0.090 |
| Sarilumab | 2023 | Godolphin | PRISMA | Mortality | RCT | 898 | 0.80 (0.61;1.04;0.09) | S: 0.123 |
| Tocilizumab | 2021 | Avni | PRISMA | Mortality | RCT | 6,481§ | 0.89 (0.82-0.96) | S: 0.064 |
| Tocilizumab | 2021 | Hariyanto | PRISMA | Mortality | OBS, RCT | 13,412§ | 0.54 (0.42;0.71;<0.00001) | S: 0.340 |
| Tocilizumab | 2021 | Juul | PRISMA | Mortality | RCT | NA | 1.03 (0.72;1.46;p=0.89) | No association |
| Tocilizumab | 2021 | Kow | - | Mortality | RCT | 2,057 | 0.83 (0.66–1.05) | No association |
| Tocilizumab | 2021 | Mahroum | PRISMA | Mortality | OBS | 3,657 | 0.74 (0.55;1.01;p=0.057) | No association |
| Tocilizumab | 2021 | Malgie | PRISMA | Mortality | OBS | 1,358 | 0.27 (0.12;0.59) | M: 0.722 |
| Tocilizumab | 2021 | Nugroho | PRISMA | Survival | OBS, RCT | 2,112 | 1.65 (1.37;2.00) | S: 0.276 |
| Tocilizumab | 2021 | Sarfraz | PRISMA | Mortality | OBS, RCT | 2,120 | 0.42 (0.26;0.69;P=0.0005) | S: 0.478 |
| Tocilizumab | 2021 | Selvaraj | PRISMA | Mortality | RCT | 3,358 | 0.89 (0.80;0.98;p=0.02) | S: 0.064 |
| Tocilizumab | 2022 | Boppana | PRISMA | Mortality | RCT | 3,358 | 0.87 (0.73;1.03;p=0.11) | No association |
| Tocilizumab | 2022 | Gupta | PRISMA | Mortality | RCT | 1,651 | 0.90 (0.76;1.07) | No association |
| Tocilizumab | 2022 | Lim | PRISMA | Mortality | OBS | 2,035 | 0.52 (0.36;0.75) | S: 0.361 |
| Tocilizumab | 2022 | Lim | PRISMA | Mortality | RCT | 5,696 | 0.71 (0.55;0.92) | S: 0.189 |
| Tocilizumab | 2022 | Mutua | PRISMA | Mortality | RCT | 3,358 | 0.87 (0.73-1.04) | No association |
| Tocilizumab | 2022 | Piscoya | PRISMA | Mortality | OBS, RCT | 4,689 | 0.89 (0.81;0.98;p=0.03) | S: 0.064 |
| Tocilizumab | 2022 | Zhang | - | Mortality | RCT | 3,358 | 0.89 (0.80;0.99) | S: 0.064 |
| Tocilizumab | 2023 | Godolphin | PRISMA | Mortality | RCT | 898 | 0.82 (0.71;0.95;0.008) | S: 0.109 |
| ***Antimalarials*** | | | | | | | | |
| Hydroxychloroquine & Chloroquine | 2021 | Qiu | PRISMA | Mortality | OBS, RCT | 4,054 | 1.21 (0.88;1.68) | No association |
| Hydroxychloroquine & Chloroquine | 2022 | Di Stefano | PRISMA | Mortality | RCT | 412 | RD: -0,01 (-0.06;0.04) | No association |
| Hydroxychloroquine | 2020 | Talaie | PRISMA | Mortality | OBS, RCT | 271 | 1.57 (1.03;2.39) | S: 0.249 |
| Hydroxychloroquine | 2020 | Wang | PRISMA | Mortality | OBS, RCT | NA | 1.08 (0.81;1.44) | No association |
| Hydroxychloroquine | 2020 | Das | PRISMA | TTVC | OBS | 415 | MD: 1.14 (-11.98;14.26;p=0.86) | No association |
| Hydroxychloroquine | 2020 | Das | PRISMA | TTVC | RCT | 415 | MD: 1.55 (-0.7;3.79;p=0.18) | No association |
| Hydroxychloroquine | 2020 | Mega | PRISMA | Mortality | OBS, RCT | 1,311 | 1.26 (0.66;2.39) | No association |
| Hydroxychloroquine | 2020 | Shamshirian | PRISMA | Mortality | OBS, RCT | NA | 0.86 (0.71;1.03) | No association |
| Hydroxychloroquine | 2021 | Zang | PRISMA | RT-PCR | OBS, RCT | 441 | 1.11 (0.77;1.59;P=0.591) | No association |
| Hydroxychloroquine | 2021 | Bignardi | PRISMA | Mortality | RCT | 7,629§ | 1.09 (0.98;1.20) | No association |
| Hydroxychloroquine | 2021 | Budhathoki | PRISMA | Mortality | OBS, RCT | 388 | 1.16 (0.92-1.46) | No association |
| Hydroxychloroquine | 2021 | Cheng | - | Mortality | OBS, RCT | NA | 1.01 (0.81;1.25) | No association |
| Hydroxychloroquine | 2021 | Choudhuri | PRISMA | Mortality | OBS, RCT | NA | 1.003 (0.98;1.02) | No association |
| Hydroxychloroquine | 2021 | Di Castelnuovo | PRISMA | Mortality | OBS, RCT | NA | 0.80 (0.69-0.93) | S: 0.123 |
| Hydroxychloroquine | 2021 | Juul | PRISMA | Mortality | RCT | NA | 1.09 (0.99;1.20;p=0.08) | No association |
| Hydroxychloroquine | 2021 | Kumar | PRISMA | Mortality | OBS, RCT | 4,215 | 1.10 (1.00;1.20) | No association |
| Hydroxychloroquine | 2021 | Tanni | PRISMA | Mortality | RCT | 714 | RD: 0.00 (-0.01;0.02;p=0.98) | No association |
| Hydroxychloroquine | 2022 | De Moraes | PRISMA | Mortality | RCT | 3,377 | 1.09 (0.99;1.19) | No association |
| ***Antivirals*** | | | | | | | | |
| Antivirals | 2020 | Talaie | PRISMA | Mortality | OBS, RCT | NA | 0.83 (0.55;1.24) | No association |
| Antivirals | 2021 | Qiu | PRISMA | Mortality | OBS, RCT | 9,488 | 0.91 (0.80;1.04) | No association |
| Favipiravir | 2020 | Prakash | PRISMA | Clin. improvement | OBS, RCT | 200 | 1.63 (1.07;2.48) | S: 0.269 |
| Favipiravir | 2022 | Lan | PRISMA | Mortality | RCT | NA | 0.99 (0.60;1.63) | No association |
| IFN-beta | 2021 | Kumar | PRISMA | Mortality | RCT | 2,343 | 0.59 (0.91;1.12) | No association |
| IFN-beta | 2021 | Juul | PRISMA | Mortality | RCT | NA | 0.75 (0.30;1.88;p=0.54) | No association |
| IFN-beta | 2021 | Nakhlband | PRISMA | Discharge rate | OBS, RCT | NA | 3.05 (1.09;5.01) | M: 0.615 |
| IFN | 2022 | Xue | - | Mortality | RCT | NA | 0.416 (0.217;0.797) | S: 0.484 |
| Lopinavir/ritonavir | 2020 | Bhattacharyya | PRISMA | Mortality | OBS, RCT | 104 | 1.208 (0.563–2.592) | No association |
| Lopinavir/ritonavir | 2020 | Verdugo | PRISMA | Mortality | RCT | 133 | 0.77 (0.45;1.30) | No association |
| Lopinavir/ritonavir | 2020 | Wang | PRISMA | Mortality | OBS, RCT | NA | 0.68 (0.39;1.19) | No association |
| Lopinavir/ritonavir | 2021 | Juul | PRISMA | Mortality | RCT | NA | 1.01 (0.92;1.12;p=0.77) | No association |
| Remdesivir | 2020 | Prasad | PRISMA | Mortality | OBS, RCT | 696 | 0.74 (0.40;1.37) | No association |
| Remdesivir | 2020 | Piscoya | PRISMA | Mortality | RCT | 691 | 0.71 (0.39;1.28) | No association |
| Remdesivir | 2020 | Verdugo | PRISMA | Mortality | RCT | 1,080 | 0.70 (0.46;1.05) | No association |
| Remdesivir | 2020 | Wang | PRISMA | Mortality | OBS, RCT | NA | 0.64 (0.44;0.94) | S: 0.246 |
| Remdesivir | 2021 | Al-Abdouh | PRISMA | Mortality | RCT | 3,818 | RD: -0.01 (-0.03;0.01) | No association |
| Remdesivir | 2021 | Enoki | - | Mortality | RCT | 1,080 | 0.67 (0.37;1.19) | No association |
| Remdesivir | 2021 | Gholamhoseini | PRISMA | Mortality | OBS, RCT | NA | 0.769 (0.563;1.050;p=0.098) | No association |
| Remdesivir | 2021 | Juul | PRISMA | Mortality | RCT | 3812 | 0.93 (0.82;1.07;p=0.31) | No association |
| Remdesivir | 2021 | Okoli | PRISMA | Mortality | RCT | 7,143 | 0.94 (0.82;1.07) | No association |
| Remdesivir | 2021 | Rezagholizadeh | PRISMA | 28-day recovery | RCT | NA | 1.09 (1.04;1,15) | S: 0.048 |
| Remdesivir | 2022 | Tanni | PRISMA | Mortality | RCT | 4,083 | RD: -0.01 (-0.02;0.01) | No association |
| Remdesivir | 2022 | Tanni | PRISMA | Mortality | OBS | 1,639 | RD: -0.02 (-0.07;0.03) | No association |
| Umifenovir | 2021 | Amani | PRISMA | RT-PCR | OBS, RCT | 108 | 1.10 (0.96;1.25) | No association |

* By convention, for Cohen's "d" of 0-0.4; 0.5-0.7 and ≥ 0.8 are considered small (S), medium (M) and large (L) effect sizes respectively (Chen, 2010). Cohen's d was calculated using the tool available at the following URL: https://www.escal.site.

§Total number of patients (experimental and control groups).

IFN: interferon; MD: mean difference; NA: not available; OBS: observational studies; RCT: ramdomized clinical trials; RD: risk difference; RT-PCR: RT-PCR negative conversion; TTVC: time to virological cure (days).
